# Multicomponent synthesis of substituted pyridines *via* a catalytic intermolecular aza-Wittig/Diels–Alder sequence†

**DOI:** 10.1039/d2ra05018h

**Published:** 2022-09-15

**Authors:** Mary E. Bayana, J. Steven Wailes, Stephen P. Marsden

**Affiliations:** School of Chemistry, University of Leeds Leeds LS2 9JT UK s.p.marsden@leeds.ac.uk; Institute of Process Research, University of Leeds Leeds LS2 9JT UK; Syngenta, Jealott's Hill International Research Centre Bracknell Berkshire RG42 6EY UK

## Abstract

A three-component synthesis of polysubstituted pyridines has been developed, based upon the synthesis of 2-azadienes by a redox-neutral catalytic intermolecular aza-Wittig reaction and their subsequent Diels–Alder reactions. The two-pot process has been demonstrated using a range of aryl and heteroaromatic aldehydes, substituted α,β-unsaturated acids and push–pull enamines, to give rapid access to diverse tri- and tetrasubstituted pyridines.

## Abstract 2

A three-component synthesis of polysubstituted pyridines, based upon the Diels–Alder reactions of 2-azadienes formed by a novel redox-neutral catalytic intermolecular aza-Wittig reaction.

## Graphical abstract

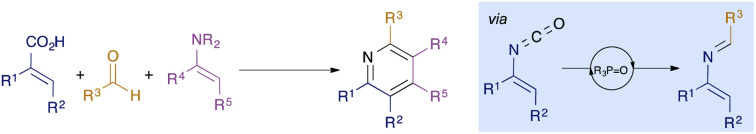

## Introduction

**Scheme 1:**
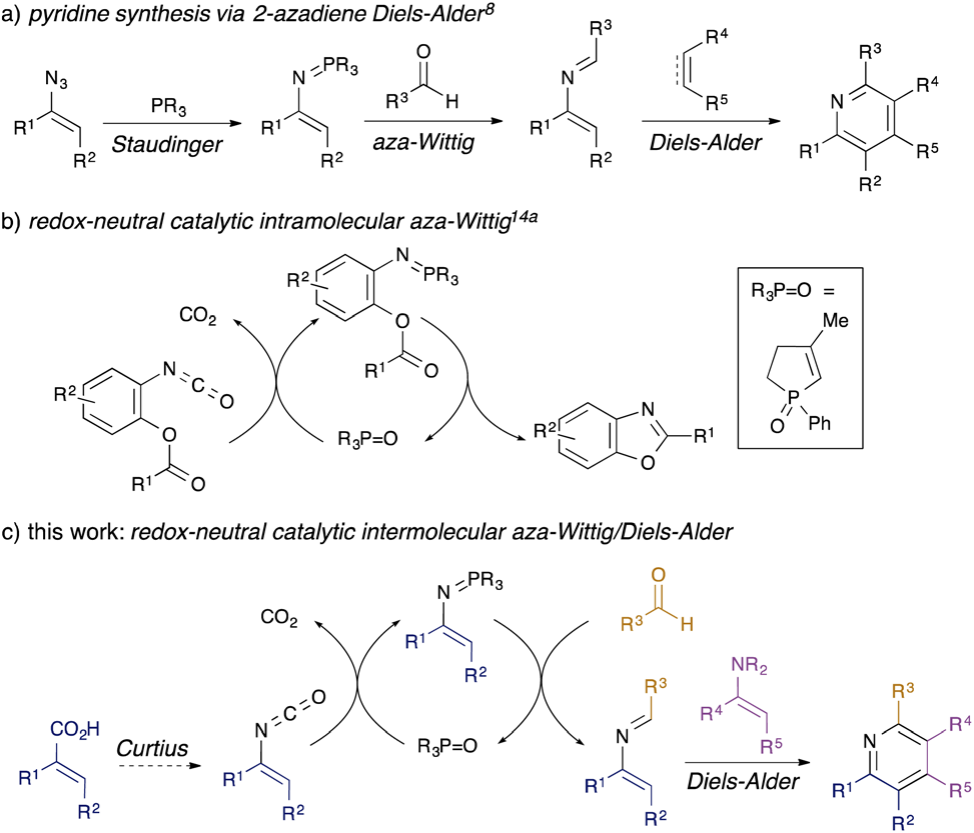
Background to the proposed work.

Substituted pyridines are commonly found motifs in agrochemical^1^ and pharmaceutical products, where they are the most prevalent heterocyclic structure.^2^ Amongst methods for *de novo* synthesis of pyridines,^3^ classical reactions such as the Hantzsch^4^ and Bohlmann-Rahtz^5^ syntheses are often limited in scope by the requirement for multiple electron-withdrawing groups in the reacting components. By contrast, [4 + 2] cycloadditions have become a key approach to the synthesis of pyridines^6^ since the nitrogen atom can be incorporated either in the diene (as 1-azadienes^7^ or 2-azadienes^8,9^) or in the dienophile,^10^ leading to a range of possible substitution patterns. In the case of 2-azadienes, however, the lack of general, sustainable methods towards their synthesis has limited their use. They are generally prepared by aza-Wittig condensation of carbonyl compounds with vinylic phosphazenes,^8^ themselves prepared by Staudinger reaction of phosphines with alkenyl azides (Scheme 1, panel a).^11^ The limited availability of alkenyl azides, safety issues associated with their preparation and handling, and the generation of stoichiometric phosphine oxide waste all conspire to limit the utility of this overall approach.

Recently, a number of organocatalytic processes have been developed for reactions that typically generate stoichiometric phosphine oxide waste,^12^ including Staudinger^13^ and aza-Wittig reactions.^14,15^ These take two different approaches: in one variant, a redox-cycling method is used, wherein the P(v) phosphane oxide is reduced *in situ* by a silane to generate a P(iii) species which goes on to perform Staudinger/aza-Wittig chemistry, regenerating the P(v) oxide.^13,15^ We developed the first examples of a redox-neutral approach to catalytic aza-Wittig cyclisations (Scheme 1, panel b),^14*a*^ wherein the reactive P(v) iminophosphorane is generated by metathesis of the P(v) phosphane oxide with an isocyanate; intramolecular aza-Wittig cyclisation produces useful heterocycles^14^ and reforms the P(v) oxide.

We were attracted to the idea of using redox-neutral catalytic aza-Wittig chemistry to generate 2-azadienes: in contrast to alkenyl azides required for stoichiometric or redox-cycling catalytic variants, the requisite vinyl isocyanates are readily available through Curtius rearrangement of unsaturated acyl azides prepared from simple α,β-unsaturated carboxylic acids, which would greatly improve synthetic access. Herein, we describe the successful development of a flexible three-component synthesis of pyridines *via* a redox-neutral catalytic intermolecular aza-Wittig/[4 + 2] cycloaddition sequence (Scheme 1, panel c).

## Results and discussion

Whilst the intramolecular variant of the redox-neutral catalytic aza-Wittig reaction has been established,^14^ the intermolecular variant is conceptually more difficult, since the desired reaction of iminophosphorane with aldehyde is in direct competition with the known and facile catalytic condensation with isocyanate to generate carbodiimide.^16^ Indeed, in their original work on isocyanate dimerisation, Monagle and Campbell reported that carrying out the reaction in neat benzaldehyde as solvent gave only a low (20%) isolated yield of the imine aza-Wittig product.^16*a*^ Nevertheless, we reasoned that we might exert control over the two competing intermolecular processes by minimising the concentration of isocyanate present in the reaction mixture through slow addition. Initial scoping experiments using phenyl isocyanate and benzaldehyde encouraged us that this might indeed be possible (see ESI†), and so we proceeded to examine the behaviour of substituted alkenyl isocyanates. It has been reported that such isocyanates readily polymerise, even at room temperature, at concentrations above 0.2 M,^17^ so we used this concentration as a starting point for alkenyl isocyanate preparation. Styryl isocyanate 3a was prepared *via* Curtius rearrangement of the corresponding cinnamic acid (1a)-derived acyl azide 2a (Table 1). This solution was then added dropwise over 2 hours to a solution of the phosphine oxide catalyst 4 and aldehyde 5a at 110 °C in toluene. We were pleased to find that the 2-azadiene 6a was formed in 50% conversion as determined by ^1^H NMR integration of the aldehyde 5a and imine 6a signals. Addition of 2.2 equivalents of enamine 7a to the resulting solution^18^ and heating at 110 °C for 18 hours gave pyridine 8a in 16% isolated yield (Table 1, entry 1). We also isolated 22% of pyridine 9, formed by the [4 + 2] cycloaddition of two azadiene molecules, as reported by Palacios *et al.*^8*e*^ The use of excess enamine 7 was therefore maintained throughout the subsequent optimisation to minimise competition from the undesired self-dimerisation of azadiene. In order to optimise the yield of the desired pyridine 8a, a number of Lewis acid additives were investigated and shown to promote the cyclocondensation at room temperature. In nearly all cases, this led to the predominant formation of the cross-coupled pyridine 8a, with MgBr_2_ providing the highest yield (entry 2), in 38% overall yield from cinnamic acid 1a. ^1^H NMR studies showed this to represent an 83% conversion from the 2-azadiene intermediate 6a, and the overall yield represents a *ca.* 80% average yield per step over the four-step sequence. The reactions generally required stoichiometric Lewis acid, although NbCl_5_ could be used at 10 mol% without loss of efficiency (entry 12).

**Table 1:** Optimisation of the Diels–Alder cyclocondensation^a^

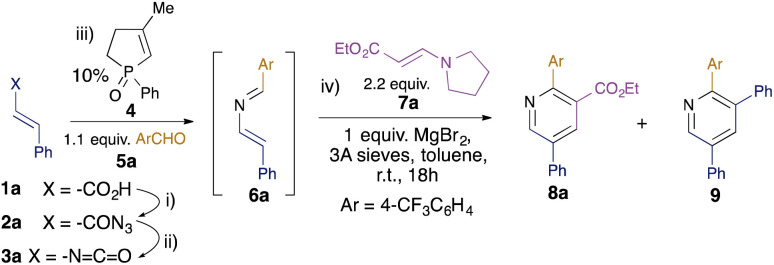			
Entry	Variation from standard cond'ns^b^	Yield 8a^c^^,^^d^ (%)	Yield 9^c^ (%)
1	No MgBr_2_, 110 °C	16 (n/d)	22
2	None	38 (83)	3
3	BF_3_·OEt_2_	27 (57)	9
4	Sc(OTf)_3_	25 (58)	6
5	Yb(OTf)_3_	8 (20)	2
6	AlCl_3_	17 (31)	10
7	SnCl_4_	11 (20)	8
8	FeCl_3_	26 (45)	12
9	Ti(O^i^Pr)_4_	12 (19)	15
10	Y(OTf)_3_	17 (26)	7
11	NbCl_5_	29 (55)	10
12	10% NbCl_5_	29 (55)	10

a Reaction conditions: (i) 1a, DPPA, NEt_3_, PhMe, rt, 1 h; (ii) PhMe, 110 °C, 30 min; (iii) 10 mol% catalyst 4, 4-CF_3_C_6_H_4_CHO 5a, 110 °C, 2 h; (iv) enamine 7a, 1 eq. MgBr_2_, 3 Å MS, rt, 16 h.

b Variation from standard conditions for step (iv).

c Yields calculated from cinnamic acid 1a.

d Values in parentheses are conversions calculated from azadiene 6a.

With standard reaction conditions in hand, the substrate scope of the aldehyde was explored (Table 2). A range of substituted benzaldehydes were tolerated, including the presence of synthetically-useful electron-withdrawing functionality (cyano in 8d, acetyl in 8f, nitro in 8h) and halides (chloro in 8e, bromo in 8g); however, electron-rich aldehydes such as 4-methoxybenzaldehyde were not well tolerated (failure of 8c). Various heteroaryl-substituted aldehydes were also tolerated (*e.g.*8i, 8j, 8n–8w), and a carboxyalkyl substituent could also be introduced using ethyl glyoxalate as the aldehyde (8k). In reactions using the *m*-(trifluoromethyl)cinnamic acid 1c, we found that the intermediate dihydropyridine was somewhat resistant to oxidation to the products 8m–8w, and so the reactions were treated with 5% Pd/C at reflux for 6 hours to promote the final dehydrogenation.

**Table 2:** Substrate scope: variation in aldehyde^a^

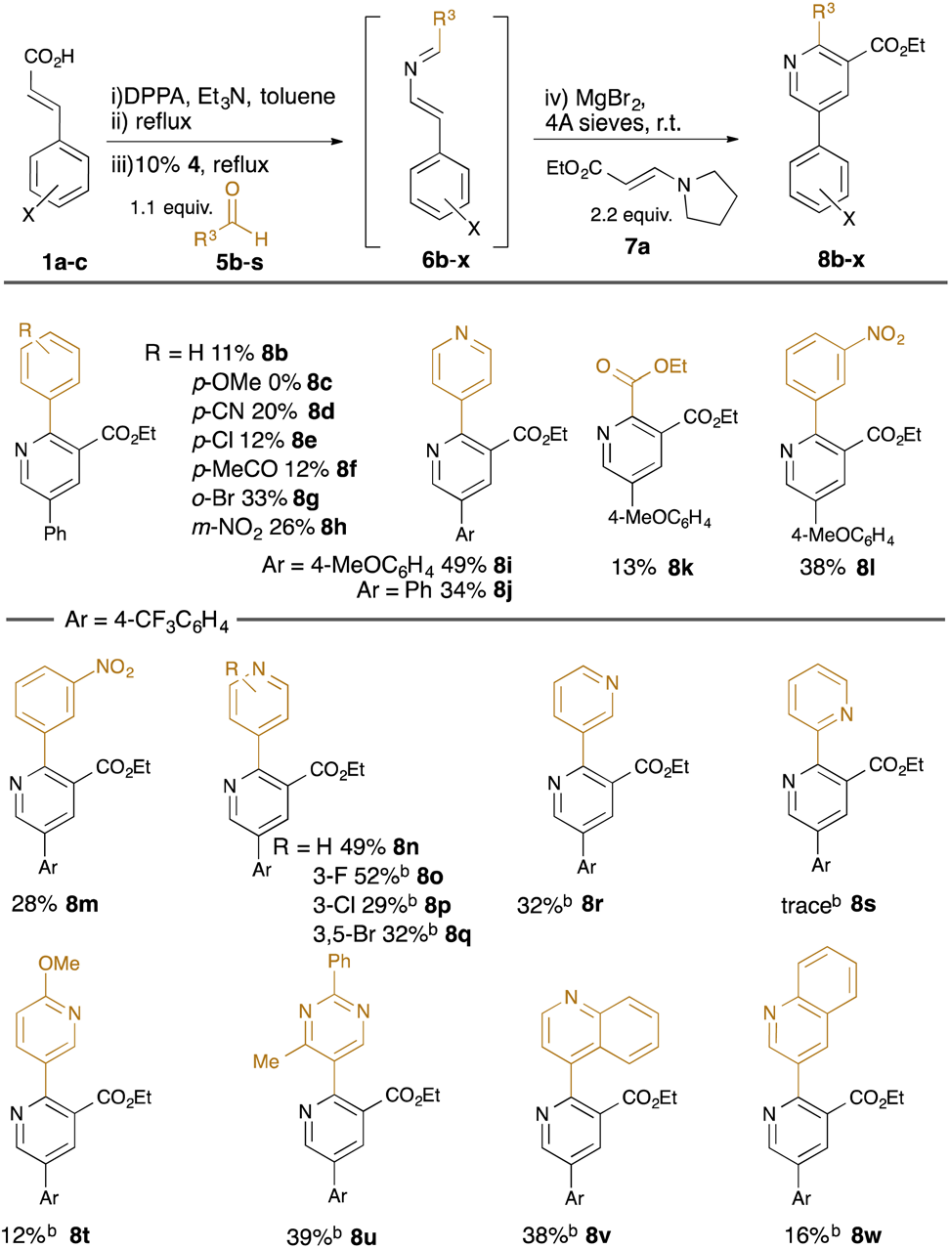

a Isolated yields calculated from cinnamic acids 1.

b After addition of 5% Pd/C and heating (110 °C, 6 h).

Further variation can be achieved using alternative α,β-unsaturated acids (Table 3). A β-heteroaryl substituent was found to be well tolerated (8x), and 2,3,5,6-tetrasubstituted pyridines 8y, 8z, and 8aa could be accessed using disubstituted acid substrates, albeit in moderate yield compared with analogous monosubstituted precursors (*e.g.*8n–8q).

**Table 3:** Substrate scope: variation in unsaturated acid^a^

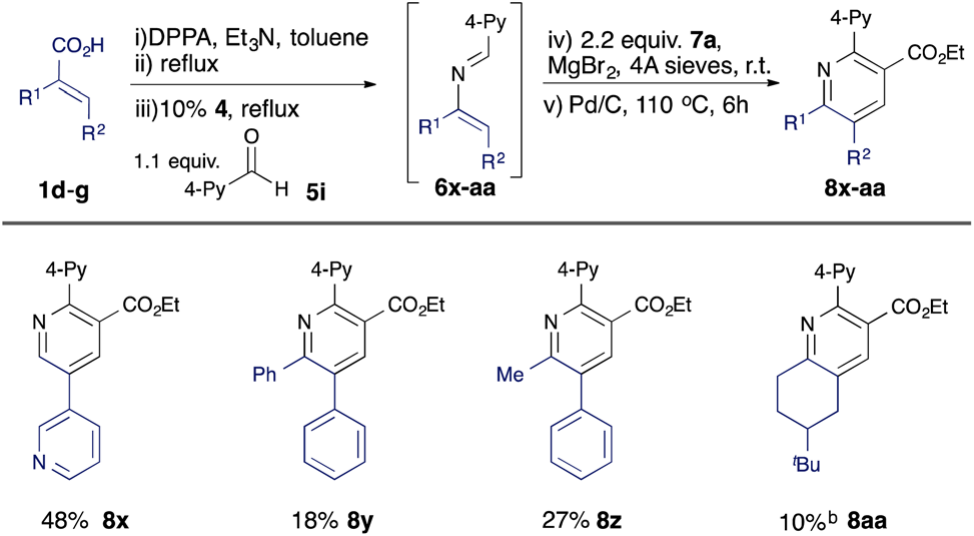

a Isolated yield from unsaturated acids 1.

b Step (v) not required.

Finally, we explored the use of alternative enamine dienophiles 7b–e to vary substitution in the 3- and 4-positions (Table 4). Incorporation of alternative electron-withdrawing groups into the push–pull enamine was tolerated (*e.g.* toluenesulfonyl in 8ab, acetyl in 8ac), while a contiguously-tetrasubstituted pyridine 8ad could be constructed from a ketoester-derived enamine 7d. The importance of the electron-withdrawing substituent on the enamine was, however, highlighted by the poor yield of pyridine 8af derived from cyclopentenyl enamine 7e.

**Table 4:** Substrate scope: variation in enamine^a^

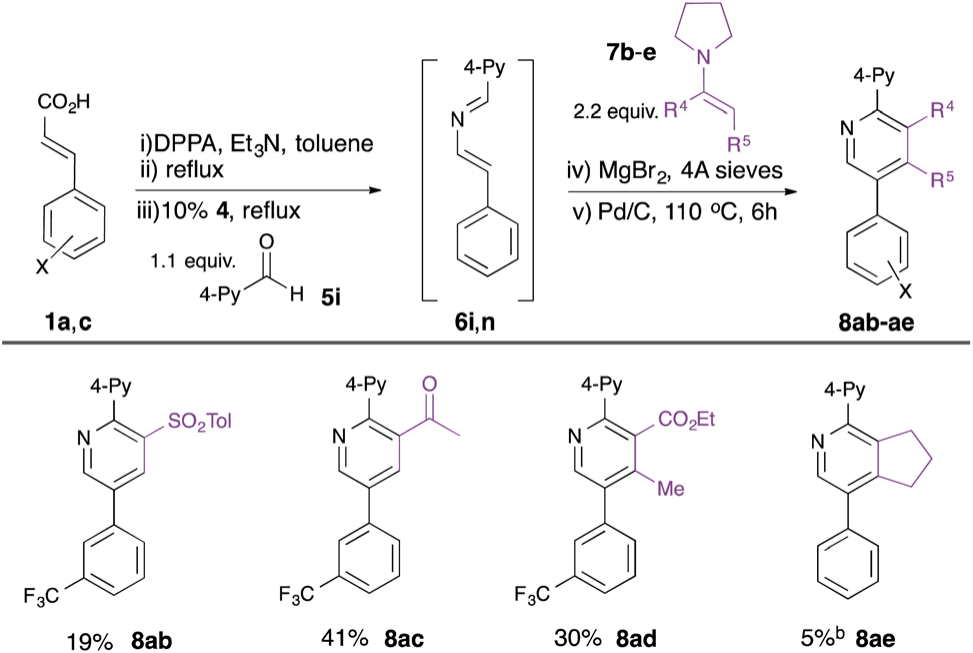

a Isolated yield from unsaturated acids 1.

b Step (v) not required.

## Conclusions

In conclusion, a two-pot, three-component procedure for the preparation of substituted pyridines has been established, exploiting the first synthetic demonstrations of redox-neutral intermolecular catalytic aza-Wittig to generate various 2-azadienes for subsequent Diels–Alder reactions. Specifically, the ready availability and stability of α,β-unsaturated carboxylic acids 1 as replacements for vinyl azide precursors to the azadiene constitutes a practical convenience. The overall yields for the 4–5 step sequence (average 28%) equate to a *ca.* 75% efficiency per step. Moreover, the three-component protocol facilitates ready access to a range of diverse substituents and substitution patterns on the pyridines, exemplified by 30 successful examples. Further synthetic applications of the intermolecular catalytic aza-Wittig reaction are readily anticipated.

## Experimental

For general experimental protocols, see ESI.†**CAUTION**: All azides should be treated as potentially explosive: the acyl azides were routinely prepared and handled behind a blast shield.

### General procedure A for the preparation of pyridines 8

A solution of the cinnamic acid 1 (1.0 mmol), diphenylphosphoryl azide (200 μl, 0.9 mmol) and triethylamine (150 μl, 1.0 mmol) in toluene (2.0 ml) was stirred at room temperature for 90 minutes then added to saturated NaHCO_3_ solution (20 ml). The organic phase was diluted with EtOAc (20 ml), the phases separated and the organic phase was washed with water (2 × 20 ml) then brine (20 ml), dried (MgSO_4_) and evaporated *in vacuo* at room temperature to give the acyl azide which was identified by crude ^1^H NMR and IR and used without purification (isolated yields calculated from cinnamic acid since acyl azides were not evaporated to complete dryness for safety). A solution of the acyl azide in toluene (5.0 ml) was heated under reflux. The reaction was monitored by IR for the disappearance of the azide signal (2142 cm^−1^) and appearance of the isocyanate signal at (2259 cm^−1^). Once formation of the isocyanate was complete (∼30 min) the solution was cooled to room temperature and added dropwise over 2 hours to a stirred solution of the aldehyde 5 (1.1 mmol) and 3-methyl-1-phenyl-2-phospholene-1-oxide 4 (19 mg, 10 mol%) in toluene (1.0 ml) heated under reflux. The reaction mixture was cooled to room temperature and the enamine 7 (2.0 mmol), magnesium bromide (0.18 g, 1.0 mmol) and 4 Å molecular sieves added and stirred at room temperature overnight then filtered through cotton wool and saturated NaHCO_3_ solution (20 ml) and EtOAc (20 ml) added. The phases were separated and aqueous phase extracted with EtOAc (2 × 20 ml). The combined organic extracts were washed with brine (40 ml), dried (MgSO_4_) and evaporated *in vacuo*. The residue was subsequently purified by flash silica column chromatography.

### General procedure B for the preparation of pyridines 8

A solution of the cinnamic acid 1 (1.0 mmol), diphenylphosphoryl azide (200 μl, 0.9 mmol) and triethylamine (150 μl, 1.0 mmol) in toluene (2.0 ml) was stirred at room temperature for 90 minutes then added to saturated NaHCO_3_ solution (20 ml). The organic phase was diluted with EtOAc (20 ml), the phases separated and the organic phase was washed with water (2 × 20 ml) then brine (20 ml), dried (MgSO_4_) and evaporated *in vacuo* at room temperature to give the acyl azide which was identified by crude ^1^H NMR and IR and used without purification (isolated yields calculated from cinnamic acid since acyl azides were not evaporated to complete dryness for safety). A solution of the acyl azide in toluene (5.0 ml) was heated under reflux. The reaction was monitored by IR for the disappearance of the azide signal (2142 cm^−1^) and appearance of the isocyanate signal at (2259 cm^−1^). Once formation of the isocyanate was complete (∼30 min) the solution was cooled to room temperature and added dropwise over 2 hours to a stirred solution of the aldehyde 5 (1.1 mmol) and 3-methyl-1-phenyl-2-phospholene-1-oxide 4 (19 mg, 10 mol%) in toluene (1.0 ml) heated under reflux. The reaction mixture was cooled to room temperature and the enamine 7 (2.0 mmol), magnesium bromide (0.18 g, 1.0 mmol) and 4 Å molecular sieves added and stirred at room temperature overnight then 5% Pd/C (50 mg) added and the reaction mixture heated under reflux for 6 hours then filtered through Celite and washed with saturated NaHCO_3_ solution (50 ml) and EtOAc (100 ml). The phases were separated and aqueous phase extracted with EtOAc (2 × 50 ml). The combined organic extracts were washed with brine (100 ml), dried (MgSO_4_) and evaporated *in vacuo*. The residue was subsequently purified by flash silica column chromatography.

## Conflicts of interest

There are no conflicts to declare.

## Supplementary Material

RA-012-D2RA05018H-s001
