# Supplementary material for: Multicomponent synthesis of substituted pyridines via a catalytic intermolecular aza-Wittig/Diels–Alder sequence
Source: RSC Adv. 2022 Sep 15;12(40):26233–7. doi: 10.1039/d2ra05018h (PMC9477066; doi:10.1039/d2ra05018h)

Electronic Supplementary Material (ESI) for RSC Advances.
This journal is © The Royal Society of Chemistry 2022

Multicomponent synthesis of substituted pyridines *via* a catalytic aza-Wittig, Diels–Alder sequence

Mary E. Bayana,[†] J. Steven Wailes,[‡] Stephen P. Marsden*,[†]

†Institute of Process Research and Development, School of Chemistry, University of Leeds, Leeds, LS2 9JT, United Kingdom

‡Syngenta, Jealott's Hill International Research Centre, Bracknell, Berkshire, RG42 6EY, United Kingdom

Supporting Information

Page Content

S2 General experimental

S3 Optimisation study of aza-Wittig reaction

S4-16 General procedures for the synthesis of pyridines **8a-ae** and compound data

S16 References

S18-47 ^{1}H and ^{13}C NMRs for compound **8a-ae**

General Experimental Techniques

Instrumentation

Proton (^{1}H) and carbon (^{13}C) magnetic resonance spectra were recorded using a Bruker DPX 300, a Bruker DRX 500 or a Bruker Advance 500 spectrometer using an internal deuterium lock. ^{1}H NMR chemical shifts (δ) are quoted in ppm downfield of trimethylsilane. ^{13}C NMR spectra were recorded with broadband proton decoupling at 75 MHz or 125 MHz. Assignments were made on the basis of chemical shift and coupling data, using ^{1}H-^{13}C HMQC, DEPT, HMBC and nOe experiments where necessary. Infra-red spectra were recorded on a Perkin Elmer Spectrum One FT-IR spectrometer, with absorption reported in wavenumbers (cm^{-1}). Mass spectra were recorded on a Bruker HCT Ultra LCMS instrument or a Bruker MicroTOF spectrometer using electrospray ionisation (ESI). Melting points were determined on a Reichert hot stage apparatus and are uncorrected.

Experimental Procedures

All reactions were carried out under an inert atmosphere of nitrogen using oven-dried glassware, unless stated. Toluene was dried prior to use using a Pure Solv MD solvent purification system. Benzaldehydes were distilled before use. Diphenylphosphoryl azide was purchased from Apollo Scientific. 3-Methyl-1-phenyl-2-phospholene 1-oxide **4** was purchased from Sigma Aldrich (85% technical grade) and was distilled shortly before use (N.B. **4** is extremely hygroscopic and poorer results are obtained with old/unpurified samples). All other solvents and reagents were purchased from commercial sources and were used without purification. Flash column chromatography was performed using Fischer Matrix silica gel (35-70 μm) or using pre-packed Biotage or Redisep silica cartridges running using Biotage Isolera or Redisep Flashmaster machines. Thin-layer chromatography was conducted using pre-coated silica plates (Merck silica Kieselgel 60F_{254}). Spots were visualised using UV florescence (λ_{max} = 254 nm) and chemical staining with potassium permanganate, bromocresol green or iodine. All chromatography eluents were BDH GPR grade and used without purification. Petrol refers to light petroleum (b.p. 40-60 °C). Enamines **7a-e** were prepared by the procedure of Pandit *et al.*.[1-4] 4-*tert*-Butylcyclohexenyl-1-carboxylic acid was prepared according to the method of Vitnik *et al.*[5]

CAUTION**:** All azides should be treated as potentially explosive and were routinely prepared and handled behind a blast shield.

Optimisation study of intermolecular aza-Wittig reaction

3-Methyl-1-phenyl-2-phospholene-1-oxide (10 mol%) and the benzaldehyde in toluene (0.2 mL/mmol) were heated under reflux with stirring. A solution of phenyl isocyanate (1.1 equivalents) in toluene (0.80 mL/mmol) was added dropwise by syringe pump over 5 hours. The reaction mixture was stirred under reflux until isocyanate consumption was complete (monitored by IR for disappearance of phenyl isocyanate signal at 2261 cm^{-1}). An aliquot was taken and solvents removed *in vacuo* to determine the ratio of imine to benzaldehyde by ^{1}H NMR. Spectroscopic data was consistent with literature values.[6]

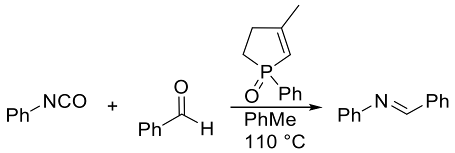

entry	conc./mol L^{-1}	mol% cat.	addition time[a]	% conversion[b]
1	1	0	-	0
2	0.2	10	-	0.5
3	1	10	-	71
4	1	10	5 h	86
5	1	10	-[c]	86[d]
6	1	1	5 h	>99[d]
7	1	5	5 h	>99[d]

a: reactions were carried out for 15 minutes until IR showed disappearance of the isocyanate absorbance (2259 cm^{-1}); b: calculated from the ratio of benzaldehyde to imine in crude ^{1}H NMR; c: reaction time 5 hours after phenyl isocyanate addition.; d: benzaldehyde distilled prior to reaction.

General Procedure A for the preparation of pyridines

A solution of the cinnamic acid (1.0 mmol), diphenylphosphoryl azide (200 μl, 0.9 mmol) and triethylamine (150 μl, 1.0 mmol) in toluene (2.0 ml) was stirred at room temperature for 90 minutes then added to saturated $NaHCO_3$ solution (20 ml). The organic phase was diluted with EtOAc (20 ml), the phases separated and the organic phase was washed with water (2 × 20 ml) then brine (20 ml), dried ($MgSO_4$) and evaporated *in vacuo* at room temperature to give the acyl azide which was identified by crude ^1H NMR and IR and used without purification (isolated yields calulated from cinnamic acid since acyl azides were not evaporated to dryness for safety). A solution of the acyl azide in toluene (5.0 ml) was heated under reflux. The reaction was monitored by IR for the disappearance of the azide signal (2142 cm^{-1}) and appearance of the isocyanate signal at (2259 cm^{-1}). Once formation of the isocyanate was complete (~30 min) the solution was cooled to room temperature and added dropwise over 2 hours to a stirred solution of the aldehyde (1.1 mmol) and 3-methyl-1-phenyl-2-phospholene-1-oxide (19 mg, 10 mol%) in toluene (1.0 ml) heated under reflux. The reaction mixture was cooled to room temperature and the enamine (2.0 mmol), magnesium bromide (0.18 g, 1.0 mmol) and 4 Å molecular sieves added and stirred at room temperature overnight then filtered through cotton wool and saturated $NaHCO_3$ solution (20 ml) and EtOAc (20 ml) added. The phases were separated and aqueous phase extracted with EtOAc (2 × 20 ml). The combined organic extracts were washed with brine (40 ml), dried ($MgSO_4$) and evaporated *in vacuo*. The residue was subsequently purified by flash silica column chromatography.

General Procedure B for the preparation of pyridines

A solution of the cinnamic acid (1.0 mmol), diphenylphosphoryl azide (200 μl, 0.9 mmol) and triethylamine (150 μl, 1.0 mmol) in toluene (2.0 ml) was stirred at room temperature for 90 minutes then added to saturated $NaHCO_3$ solution (20 ml). The organic phase was diluted with EtOAc (20 ml), the phases separated and the organic phase was washed with water (2 × 20 ml) then brine (20 ml), dried ($MgSO_4$) and evaporated *in vacuo* at room temperature to give the acyl azide which was identified by crude ^1H NMR and IR and used without purification (isolated yields calulated from cinnamic acid since acyl azides were not evaporated to dryness). A solution of the acyl azide in toluene (5.0 ml) was heated under reflux. The reaction was monitored by IR for the disappearance of the azide signal (2142 cm^{-1}) and appearance of the isocyanate signal at (2259 cm^{-1}). Once formation of the isocyanate was complete (~30 min) the solution was cooled to room temperature and added dropwise over 2 hours to a stirred solution of the aldehyde (1.1 mmol) and 3-methyl-1-phenyl-2-phospholene-1-oxide (19 mg, 10 mol%) in toluene (1.0 ml) heated under reflux. The reaction mixture was cooled to room temperature and the enamine (2.0 mmol), magnesium bromide (0.18 g, 1.0 mmol) and 4 Å molecular sieves added and stirred at room temperature overnight then 5% Pd/C (50 mg) added and the reaction mixture heated under reflux for 6 hours then filtered through celite and washed with saturated $NaHCO_3$ solution (50 ml) and EtOAc (100 ml). The phases were separated and aqueous phase extracted with EtOAc (2 × 50 ml). The combined organic extracts were washed with brine (100 ml), dried ($MgSO_4$) and evaporated *in vacuo*. The residue was subsequently purified by flash silica column chromatography.

5-Phenyl-2-(4-trifluoromethylphenyl)nicotinic acid ethyl ester (8a)

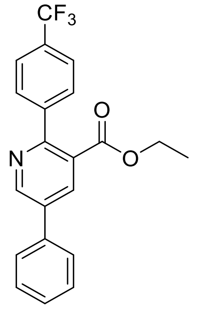

Prepared according to general procedure A using cinnamic acid (0.15 g, 1.0 mmol) and 4-trifluoromethylbenzaldehyde (0.15 ml, 1.1 mmol) and ethyl (*E*)-3-(pyrrolidin-1-yl)-2-propenoate **7** (0.34 g, 2.0 mmol). Purification by column chromatography (SiO_2, 0-10% EtOAc in hexane) gave the pyridine as yellow needles (0.12 g, 38%). M.p.= 116-120 °C ($CHCl_3$); δ_H (300 MHz, $CDCl_3$); 9.01 (1H, d, *J* 2.3 Hz, pyr-H), 8.36 (1H, d, *J* 2.3 Hz, pyr-H), 7.76-7.68 (4H, m, ArH), 7.68-7.64 (2H, m, ArH), 7.55-7.49 (2H, m, ArH), 7.48-7.43 (1H, m, ArH), 4.20 (2H, q, *J* 7.1 Hz, CH_2), 1.09 (3H, t, *J* 7.1 Hz, CH_3); δ_C (75 MHz, $CDCl_3$); 167.4 (C=O), 156.1 (ArCH), 149.6 (ArCH), 143.5 (quat.), 136.3 (ArCH), 136.2 (quat.), 135.4 (quat.), 130.6 (quat., q, *J* 32.2 Hz, $ArCCF_3$), 129.3 (ArCH), 129.0 (ArCH), 128.7 (ArCH), 127.6 (quat., q, *J* 272.3Hz, CF_3), 127.1 (ArCH), 125.0 (q, *J* 3.5 Hz, ArCH), 122.8 (quat.), 61.7 (CH_2), 13.6 (CH_2CH_3); ν_{max}/cm^{-1} (solid); 2986, 1722, 1618, 1581, 1549, 1450, 1406, 1368, 1323, 1246, 1160, 1096, 1065, 1033, 1014; HRMS (ES^+) *m/z*; $[M+H]^+$ requires 372.1211 for $C_{21}H_{17}F_3NO_2$, found: 372.1212.

3,5-Diphenyl-2-(4-trifluoromethylphenyl)pyridine (9; Table 1, entry 1)

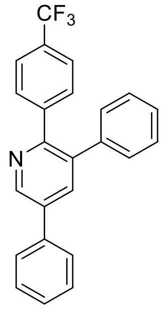

Yellow needles (0.041g, 22%). M.p.= 163-166 °C ($CHCl_3$); δ_H (300 MHz, $CDCl_3$); 8.94 (1H, d, *J* 2.4 Hz, pyr-H), 7.96 (1H, d, *J* 2.4 Hz, pyr-H), 7.68 (2H, d, *J* 6.7 Hz, ArH), 7.56-7.41 (7H, m, ArH)7.37-7.30 (3H, m, ArH), 7.25-7.20 (2H, m, ArH); δ_C (75 MHz, $CDCl_3$) 154. 2 (quat.), 146.9 (ArCH), 143.4 (q, *J* 1.1 Hz, quat.), 139.2 (quat.), 137.1 (ArCH), 136.2 (quat.), 135.7 (quat.), 130.2 (ArCH), 129.9 (quat.), 129.5 (ArCH), 129.2 (ArCH), 128.6 (ArCH), 128.4 (ArCH), 127.7 (ArCH), 127.2 (ArCH), 125.9 (quat.), 124.9 (q, *J* 4.4 Hz, ArCH), 124.0 (q, *J* 270.2 Hz, CF_3); ν_{max}/cm^{-1} (solid, diamond); 3030, 2642, 1953, 1814, 1614, 1578, 1541, 1493, 1432, 1325, 1171, 1108, 1010; HRMS (ES^+) *m/z* $[M+H]^+$ requires 376.1308 for $C_{24}H_{17}F_3N$, found: 376.1310.

2,5-Diphenylnicotinic acid ethyl ester (8b)

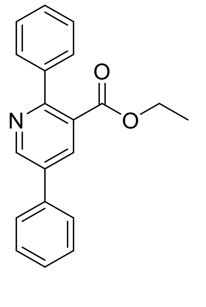

Prepared according to general procedure A using cinnamic acid (0.15 g, 1.0 mmol), benzaldehyde (0.12 ml, 1.1 mmol) and ethyl (*E*)-3-(pyrrolidin-1-yl)-2-propenoate (0.34 g, 2.0 mmol). Purification by column chromatography (SiO_2, 0-40% EtOAc in hexane) gave the pyridine **8b** as pale yellow needles (0.034 g, 11%). M.p. = 138-140 °C (EtOH, Lit.[211] 138-139 °C); δ_H (300 MHz, $CDCl_3$); 8.92 (1H, d, *J* 2.2 Hz, pyr-H), 8.22 (1H, d, *J* 2.2 Hz, pyr-H), 7.62-7.56 (2H, m, ArH), 7.56-7.54 (2H, m, ArH), 7.53-7.49 (2H, m, ArH), 7.40-7.33 (4H, m, ArH), 4.11 (2H, q, *J* 7.1 Hz, CH_2), 0.99 (3H, t, *J* 7.1 Hz, CH_3); δ_C (75 MHz, $CDCl_3$); 167.2 (C=O), 156.3 (quat.), 148.4 (ArCH), 138.8 (quat.), 135.5 (quat.), 135.0 (ArCH), 133.6 (quat.), 128.2 (ArCH), 127.7 (ArCH), 127.6 (ArCH), 127.5 (ArCH), 127.1 (ArCH), 126.3 (quat.), 126.1 (ArCH), 60.6 (CH_2), 12.6 (CH_3); ν_{max}/cm^{-1} (thin film); 3060, 2979, 2935, 1709, 1594, 1541, 1443, 1384, 1363, 1325, 1249, 1103, 1060, 1013; HRMS (ES^+) *m/z*; $[M+H]^+$ requires 304.1332 for $C_{20}H_{18}NO_2$, found: 304.1342. Spectroscopic data consistent with literature values.[7]

2-(4-Cyanophenyl)-5-phenylnicotinic acid ethyl ester (8d)

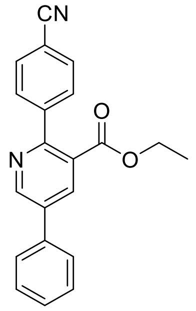

Prepared according to general procedure A using cinnamic acid (0.15 g, 1.0 mmol) 4-cyanobenzaldehyde (0.14 g, 1.1 mmol) and ethyl (*E*)-3-(pyrrolidin-1-yl)-2-propenoate (0.34 g, 2.0 mmol). Purification by column chromatography (SiO_2, 0-10% EtOAc in hexane) gave the pyridine **8d** as pale orange needles (0.066 g, 20%). M.p.= 125-128 °C (EtOAc–petrol); δ_H (300 MHz, $CDCl_3$); 9.03 (1H, d, *J* 2.2 Hz, pyr-H), 8.41 (1H, d, *J* 2.2 Hz, pyr-H), 7.80-7.75 (2H, m, ArH), 7.72-7.66 (4H, m, ArH), 7.58-7.45 (3H, m, ArH), 4.24 (2H, q, *J* 7.1 Hz, CH_2), 1.15 (3H, t, *J* 7.1 Hz, CH_3); δ_C (75 MHz, $CDCl_3$); 167.1 (C=O), 155.6 (quat.), 149.8 (ArCH), 144.5 (quat.), 136.6 (ArCH), 136.0 (quat.), 135.8 (quat.) 131.9 (ArCH), 129.5 (ArCH), 129.4 (ArCH), 128.9 (ArCH), 127.2 (ArCH), 127.1 (quat.), 118.7 (quat.), 112.3 (CN), 61.9 (CH_2), 13.8 (CH_3) ; ν_{max}/cm^{-1} (solid); 3004, 2222, 1725, 1449, 1360, 1247, 1095, 1023; HRMS (ES^+) *m/z*; $[M+H]^+$ requires 329.1285 for $C_{21}H_{17}N_2O_2$, found: 329.1276.

2-(4-Chlorophenyl)-5-phenylnicotinic acid ethyl ester (8e)

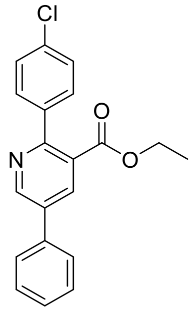

Prepared according to general procedure A using cinnamic acid (0.15 g, 1.0 mmol), 4-chlorobenzaldehyde (0.15 g, 1.1 mmol) and ethyl (*E*)-3-(pyrrolidin-1-yl)-2-propenoate (0.34 g, 2.0 mmol). Purification by column chromatography (SiO_2, 0-35% EtOAc in hexane) gave the pyridine **8e** as yellow plates (0.041 g, 12%). M.p.= 90-97 °C (EtOH); δ_H (300 MHz, $CDCl_3$); 9.01 (1H, d, *J* 2.2 Hz, pyr-H), 8.33 (1H, d, *J* 2.2 Hz, pyr-H), 7.70-7.64 (2H, d, *J* 7.4 Hz, ArH), 7.58-7.51 (4H, m, ArH), 7.50-7.42 (3H, m, ArH), 4.24 (2H, q, *J* 7.1 Hz, CH_2), 1.16 (3H, t, *J* 7.1 Hz, CH_3); δ_C (75 MHz, $CDCl_3$); 167.9 (C=O), 156.2 (quat.), 149.6 (ArCH), 138.3 (quat.), 136.4 (quat.), 136.3 (ArCH), 135.0 (quat.) 134.9 (quat.), 130.0 (ArCH), 129.3 (ArCH), 128.7 (ArCH), 128.4 (ArCH), 127.2 (ArCH), 127.1 (quat.), 61.2 (CH_2), 13.8 (CH_3); ν_{max}/cm^{-1} (solid); 3057, 2980, 2936,1915, 1727, 1594, 1541, 1486, 1445, 1402, 1384, 1365, 1323, 1249, 1212, 1095, 1060, 1011; HRMS (ES^+) *m/z*; $[M+H]^+$ requires 338.0942 for $C_{20}H_{17}{}^{35}ClNO_2$, found: 338.0957.

2-(4-Acetylphenyl)-5-phenylnicotinic acid ethyl ester (8f)

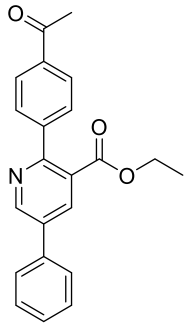

Prepared according to general procedure A using cinnamic acid (0.15 g, 1.0 mmol) and 4-acetylcarboxaldehyde (0.16 g, 1.1 mmol) and ethyl (*E*)-3-(pyrrolidin-1-yl)-2-propenoate (0.34 g, 2.0 mmol). Purification by column chromatography (SiO_2, 0-35% EtOAc in hexane) gave the pyridine **8f** as yellow needles (0.042 g, 12%). M.p.= 109-112 °C (EtOH); δ_H (300 MHz, $CDCl_3$); 9.03 (1H, d, *J* 2.2 Hz, pyr-H), 8.37 (1H, d, *J* 2.2 Hz, pyr-H), 8.05 (2H, d, *J* 8.2 Hz, H3'), 7.72-7.64 (4H, m, ArH), 7.56-7.43 (3H, m, ArH), 4.22 (2H, q, *J* 7.1 Hz, CH_2), 2.66 (3H, s, $O{=}CCH_3$), 1.11 (3H, t, *J* 7.1 Hz, CH_2CH_3); δ_C (75 MHz, $CDCl_3$); 197.8 (O=C), 167.4 (O=C), 156.2 (quat.), 149.4 (ArCH), 144.2 (quat.), 136.9 (quat.), 136.5 (ArCH), 136.1 (quat.), 135.5 (quat.), 129.3 (ArCH), 129.0 (ArCH), 128.8 (ArCH), 128.2 (ArCH), 127.4 (quat.), 127.1 (ArCH), 61.8 (CH_2), 26.8 (CH_3), 13.8 (CH_3); ν_{max}/cm^{-1} (solid); 2980, 1714, 1682, 1607, 1541, 1510, 1454, 1407, 1365, 1325, 1298, 1247,

1209, 1103, 1058, 1012; HRMS (ES$^+$) *m/z*; [M+H]$^+$ requires 346.1438 for $C_{22}H_{20}NO_3$, found: 346.1441.

2-(2-Bromophenyl)-5-phenylnicotinic acid ethyl ester (8g)

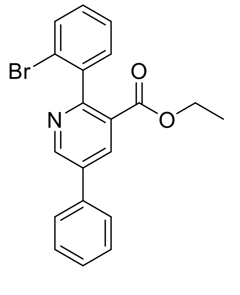

Prepared according to general procedure A using cinnamic acid (0.15 g, 1.0 mmol) 2-bromobenzaldehyde (0.13 ml, 1.1 mmol) and ethyl (*E*)-3-(pyrrolidin-1-yl)-2-propenoate (0.34 g, 2.0 mmol). Purification by column chromatography (SiO_2, 0-50% EtOAc–petrol) gave the pyridine **8g** as yellow crystals (0.12 g, 33%). M.p.= 99-100 °C ($CHCl_3$); δ_H (300 MHz, $CDCl_3$); 8.97 (1H, d, *J* 2.5 Hz, pyr-H), 8.46 (1H, d, *J* 2.5 Hz, pyr-H), 7.63 (2H, m, ArH), 7.56 (1H, m, ArH), 7.49-7.33 (5H, m, C_6H_5), 7.24-7.21 (1H, m, ArH), 4.10 (2H, q, *J* 7.2 Hz, CH_2), 0.97 (3H, t, *J* 7.2 Hz, CH_3); δ_C (75 MHz, $CDCl_3$); 166.0 (C=O), 157.5 (quat.), 149.9 (ArCH), 141.8 (quat.), 136.5 (ArCH), 136.4 (quat.), 135.7 (quat.) 132.2 (ArCH), 130.1 (ArCH), 129.5 (ArCH), 129.3 (ArCH), 128.7 (ArCH), 127.3 (ArCH), 127.2 (ArCH), 127.0 (quat.), 122.3 (quat.), 61.6 (CH_2), 13.6 (CH_3); ν_{max}/cm^{-1} (solid); 2985, 2939, 1973, 1933, 1885, 1813, 1709, 1595, 1479, 1439, 1363, 1316, 1250, 1207, 1107, 1055, 1013; HRMS (ES$^+$) *m/z*; [M+H]$^+$ requires 382.0437 for $C_{20}H_{17}{}^{79}BrNO_2$, found: 382.0440.

2-(3-Nitrophenyl)-5-phenylnicotinic acid ethyl ester (8h)

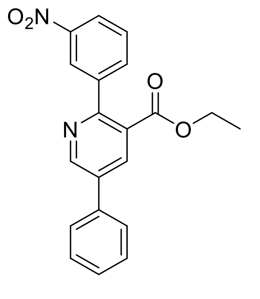

Prepared according to general procedure A using cinnamic acid (0.15 g, 1.0 mmol), 3-nitrobenzaldehyde (0.17 g, 1.1 mmol) and and ethyl (*E*)-3-(pyrrolidin-1-yl)-2-propenoate (0.34 g, 2.0 mmol). Purification by column chromatography (SiO_2, 0-30% EtOAc in petrol) gave the pyridine **8h** as yellow needles (0.10 g, 26%). M.p.= 74-78 °C (EtOAc–Petrol); δ_H (300 MHz, $CDCl_3$); 8.95 (1H, d, *J* 2.2 Hz, pyr-H), 8.40 (1H, dd, *J* 2.2, 1.6 Hz, ArH), 8.34 (1H, d, *J* 2.2 Hz, pyr-H), 8.23 (1H, ddd, *J* 8.2, 2.2, 1.1 Hz, ArH), 7.84 (1H, ddd, *J* 8.2, 1.6, 1.1 Hz, ArH), 7.63-7.52 (3H, m, ArH), 7.50-7.36 (3H, m, ArH), 4.18 (2H, q, *J* 7.1 Hz, CH_2), 1.08 (3H, t, *J* 7.1 Hz, CH_2CH_3); δ_C (75 MHz, $CDCl_3$); 165.9 (C=O), 154.0 (quat.), 148.8 (ArCH), 146.9 (quat.), 140.5 (quat.), 135.6 (ArCH), 135.0 (quat.), 134.8 (quat.), 133.8 (ArCH), 128.3 (ArCH), 128.0 (ArCH), 127.8 (ArCH), 126.1 (ArCH), 125.8 (quat.), 122.9 (quat.), 122.3 (ArCH) 60.9 (CH_2), 12.8 (CH_3); ν_{max}/cm^{-1} (solid); 2990, 1729, 1583, 1526, 1448, 1352, 1241, 1079, 1040; HRMS (ES$^+$) *m/z*; [M+H]$^+$ requires 349.1183 for $C_{22}H_{17}N_2O_4$, found: 349.1192.

5-(4-Methoxyphenyl)-[2,4']bipyridinyl-3-carboxylic acid ethyl ester (8i)

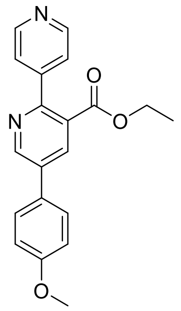

Prepared according to general procedure A using 4-methoxycinnamic acid (0.18 g, 1.0 mmol), 4-pyridinecarboxaldehyde (0.10 ml, 1.1 mmol) and ethyl (*E*)-3-(pyrrolidin-1-yl)-2-propenoate (0.34 g, 2.0 mmol). Purification by column chromatography (SiO_2, 30-100% EtOAc in petrol) gave the pyridine **8i** as yellow needles (0.16 g, 49%). M.p. = 88-94 °C (EtOAc–petrol); δ_H (300 MHz, $CDCl_3$); 8.99 (1H, d, *J* 2.2 Hz, pyr-H), 8.70 (2H, d, *J* 4.4 Hz, pyr-H), 8.34 (1H, d, *J* 2.2 Hz, pyr-H), 7.64-7.58 (2H, m, ArH), 7.50 (2H, d, *J* 4.4 Hz, pyr-H), 7.08-7.02 (2H, m, ArH), 4.22 (2H, q, *J* 7.1 Hz, CH_2), 3.88 (3H, s, OCH_3), 1.12 (3H, t, *J* 7.1 Hz, CH_3); δ_C (75 MHz, $CDCl_3$); 167.1 (C=O), 160.4 (quat.), 154.1 (quat.), 149.4 (ArCH), 149.3 (quat.), 147.9 (quat.), 135.8 (ArCH), 135.6 (quat.), 128.4 (ArCH), 128.3 (quat.), 127.1 (ArCH), 123.4 (ArCH), 114.8 (ArCH), 61.9 (CH_2), 55.4 (OCH_3), 13.6 (CH_3); ν_{max}/cm^{-1} (solid); 2978, 2836, 1714, 1597, 1519, 1441, 1406, 1365, 1322, 1295, 1249, 1184, 1120, 1107, 1061, 1017; HRMS (ES^+) *m/z*; $[M+H]^+$ requires 335.1390 for $C_{20}H_{19}N_2O_3$, found: 335.1400.

5-Phenyl-[2,4']bipyridinyl-3-carboxylic acid ethyl ester (8j)

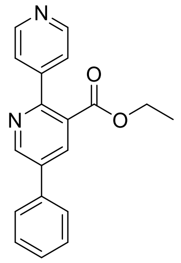

Prepared according to general procedure A using cinnamic acid (0.15 g, 1.0 mmol) and 4-pyridinecarboxaldehyde (0.10 ml, 1.1 mmol) and ethyl (*E*)-3-(pyrrolidin-1-yl)-2-propenoate (0.34 g, 2.0 mmol). Purification by column chromatography (SiO_2, 0-80% EtOAc in petrol) gave the pyridine **8i** as colourless needles (0.10 g, 34%). M.p.= 113-114 °C ($CHCl_3$); δ_H (300 MHz, $CDCl_3$); 9.03 (1H, d, *J* 2.2 Hz, pyr-H), 8.72 (2H, d, *J* 6.0 Hz, pyr-H), 8.39 (1H, d, *J* 2.2 Hz, pyr-H), 7.70-7.65 (2H, d, *J* 6.0 Hz, pyr-H), 7.58-7.47 (5H, m, ArH), 4.23 (2H, q, *J* 7.1 Hz, CH_2), 1.12 (3H, t, *J* 7.1 Hz, CH_3); δ_C (75 MHz, $CDCl_3$); 167.1 (C=O), 149.9 (ArCH), 149.7 (quat.), 147.7 (quat.), 136.5 (ArCH), 136.1 (quat.), 136.0 (quat.), 130.1 (quat.), 129.4 (ArCH), 129.1 (quat.), 128.9 (ArCH), 127.2 (ArCH), 123.3 (ArCH), 61.9 (CH_2), 13.6 (CH_3); ν_{max}/cm^{-1} (solid); 2982, 1722, 1600, 1448, 1320, 1254, 1100, 1068, 1028; HRMS (ES+) *m/z*; $[M+H]^+$ requires 305.1285 for $C_{19}H_{17}N_2O_2$, found: 305.1270.

5-(4-Methoxyphenyl)pyridine-2,3-diethyl carboxylate (8k)

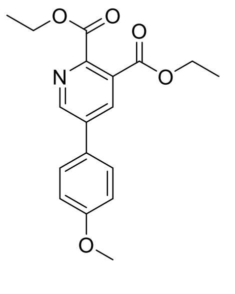

Prepared according to general procedure A using 4-methoxycinnamic acid (0.18 g, 1.0 mmol), ethyl glyoxylate (0.15 ml, 1.5 mmol) and ethyl (*E*)-3-(pyrrolidin-1-yl)-2-propenoate (0.25 g, 1.5 mmol). Purification by column chromatography (SiO_2, 0-80% EtOAc in petrol) gave the pyridine **8k** as orange oil (0.043 g, 13%). δ_H (300 MHz, $CDCl_3$); 8.94 (1H, d, *J* 2.2 Hz, pyr-H), 8.25 (1H, d, *J* 2.2 Hz, pyr-H), 7.58 (2H, d, *J* 9.0 Hz, ArH), 7.04 (2H, d, *J* 9.0 Hz, ArH), 4.48 (2H, q, *J* 7.1 Hz, CH_2CH_3), 4.42 (2H, q, *J* 7.1 Hz, CH_2CH_3), 3.88 (3H, s, OCH_3), 1.44 (3H, t, *J* 7.1 Hz, CH_2CH_3), 1.40 (3H, t, *J* 7.1 Hz, CH_2CH_3); δ_C (75 MHz, $CDCl_3$); 166.1 (C=O), 165.9 (C=O), 160.5 (quat.), 149.3 (ArCH), 147.9 (quat.), 137.7 (quat.), 134.8 (ArCH), 128.5 (ArCH), 128.1 (quat.), 127.4 (quat.), 114.8 (ArCH), 62.3 (CH_2), 62.2 (CH_2), 55.5 (OCH_3), 14.2 (CH_2CH_3), 14.1 (CH_2CH_3); ν_{max}/cm^{-1} (solid); 2983, 2938, 1728, 1609, 1518, 1457, 1366, 1303, 1256, 1183, 1143, 1078, 1020; HRMS (ES+) *m/z*; $[M+Na]^+$ requires 330.1336 for $C_{18}H_{20}NO_5$, found: 330.1328.

5-(4-Methoxyphenyl)-2-(3-nitrophenyl)nicotinic acid ethyl ester (8l)

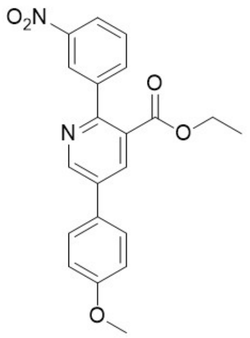

Prepared according to general procedure A using 4-methoxycinnamic acid (0.18 g, 1.0 mmol), 3-nitrobenzaldehyde (0.17 g, 1.1 mmol) and and ethyl (*E*)-3-(pyrrolidin-1-yl)-2-propenoate (0.34 g, 2.0 mmol). Crude acyl azide (0.18 g, 0.89 mmol). Purification by column chromatography (SiO_2, 0-35% EtOAc in petrol then 10-20% EtOAc in petrol) gave the pyridine **8l** as yellow needles (0.14 g, 38%). M.p.= 45-46 °C (EtOAc–Petrol); δ_H (300 MHz, $CDCl_3$); 8.99 (1H, d, *J* 2.2 Hz, pyr-H), 8.47-8.45 (1H, m, ArH), 8.36 (1H, d, *J* 2.2 Hz, pyr-H), 8.29 (1H, ddd, *J* 8.2, 2.2, 1.1 Hz, ArH), 7.93-7.88 (1H, m, ArH), 7.66-7.59 (3H, m, ArH), 7.08-7.02 (2H, m, ArH), 4.25 (2H, q, *J* 7.1 Hz, CH_2), 3.88 (3H, s, OCH_3), 1.16 (3H, t, *J* 7.1 Hz, CH_2CH_3); δ_C (75 MHz, $CDCl_3$); 167.1 (*C*=O), 160.4 (quat.), 154.3 (quat.), 149.4 (C6), 147.9 (quat.), 141.6 (quat.), 136.0 (ArCH), 135.8 (quat.), 135.4 (ArCH), 134.8 (ArCH), 130.6 (ArCH), 128.3 (ArCH), 126.8 (quat.), 123.9 (ArCH), 123.2 (ArCH), 114.6 (ArCH), 61.9 (CH_2), 55.4 (OCH_3), 13.8 (CH_3); ν_{max}/cm^{-1} (solid); 3419, 3092, 2984, 2939, 2908, 1715, 1634, 1538, 1446, 1348, 1286, 1184, 1101, 1029; HRMS (ES^+) *m/z*; $[M+H]^+$ requires 379.1288 for $C_{21}H_{19}N_2O_5$, found: 379.1285.

5-(3-Trifluoromethylphenyl)-2-(3-nitrophenyl)nicotinic acid ethyl ester (8m)

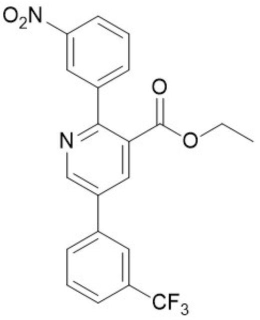

Prepared according to general procedure B using 3-trifluoromethylcinnamic acid (0.21 g, 1.0 mmol), 3-nitrobenzaldehyde (0.17 g, 1.1 mmol) and and ethyl (*E*)-3-(pyrrolidin-1-yl)-2-propenoate (0.34 g, 2.0 mmol). Crude acyl azide (0.21 g, 0.86 mmol). Purification by column chromatography (SiO_2, 0-35% EtOAc in petrol) gave the pyridine **8m** as yellow needles (0.12 g, 28%). M.p.= 127-129 °C (EtOAc–Petrol); δ_H (300 MHz, $CDCl_3$); 9.04 (1H, d, *J* 2.2 Hz, pyr-H), 8.49 (1H, s, ArH), 8.43 (1H, d, *J* 2.2 Hz, pyr-H), 8.33 (1H, dd, *J* 8.2, 1.6 Hz, ArH), 7.94-7.90 (2H, m, ArH), 7.88 (1H, d, *J* 8.2 Hz, Ar*H*), 7.77-7.63 (3H, m, ArH), 4.28 (2H, q, *J* 7.1 Hz, C*H*$_2$), 1.17 (3H, t, *J* 7.1 Hz, CH_2CH_3); δ_C (75 MHz, $CDCl_3$); 166.7 (*C*=O), 155.8 (quat.), 149.8 (C6), 148.0 (quat.), 141.3 (quat.), 137.0 (quat.), 136.9 (C4), 134.8 (ArCH), 134.5 (quat.), 132.1 (q, *J* 3.9 Hz, CCF_3), 130.5 (ArCH), 129.9 (ArCH), 129.1 (ArCH), 127.1 (quat.), 125.6 (ArCH), 124.1 (ArCH), 124.0 (ArCH), 123.5 (ArCH), 122.1 (q, *J* 119.5, CF_3), 62.1 (CH_2), 13.8 (CH_3); ν_{max}/cm^{-1} (solid); 3439, 3086, 2988, 1959, 1854, 1726, 1615, 1580, 1528, 1482, 1445, 1397, 1300, 1256, 1211, 1166, 1115, 1041; HRMS (ES^+) *m/z*; $[M+H]^+$ requires 417.1057 for $C_{21}H_{16}F_3N_2O_4$, found: 417.1055.

5-(3-Trifluoromethylphenyl)-[2,4']bipyridinyl-3-carboxylic acid ethyl ester (8n)

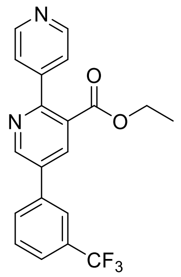

Prepared according to general procedure B using 3-trifluoromethylcinnamic acid (0.21 g, 1.0 mmol), 4-pyridinecarboxaldehyde (0.10 ml, 1.1 mmol) and ethyl (*E*)-3-(pyrrolidin-1-yl)-2-propenoate (0.34 g, 2.0 mmol). Purification by column chromatography (SiO_2, 30-100% EtOAc in petrol) gave the pyridine **8n** as yellow needles (0.16 g, 49%). M.p. = 80-82 °C (EtOH); δ_H (300 MHz, $CDCl_3$); 8.96 (1H, d, *J* 2.2 Hz, pyr-H), 8.66 (2H, dd, *J* 4.8, 1.6 Hz, pyr-H), 8.32 (1H, d, *J* 2.2 Hz, pyr-H), 7.83 (1H, s, ArH), 7.78 (1H, d, *J* 7.7 Hz, ArH), 7.67 (1H, d, *J* 7.7 Hz, ArH), 7.60 (1H, t, *J* 7.7 Hz, ArH), 7.42 (2H, dd, *J* 4.4, 1.6 Hz, pyr-H), 4.16 (2H, q, *J* 7.1 Hz, CH_2), 1.05 (3H, t, *J* 7.1 Hz, CH_3); δ_C (75 MHz, $CDCl_3$); 167.8 (C=O), 154.7 (quat.), 148.7 (ArCH), 148.6 (ArCH), 146.3 (quat.), 135.8 (quat.), 135.6 (ArCH), 133.5 (quat.), 130.6 (quat., q, *J* 32.0 Hz, CCF_3), 129.4 (ArCH), 128.9 (ArCH), 126.4 (quat.), 124.6 (ArCH), 123.1 (q, *J* 263.5 Hz, CF_3), 123.0 (ArCH), 122.9 (ArCH), 61.1 (CH_2), 12.6 (CH_3); ν_{max}/cm^{-1} (solid); 3416, 3073, 3040, 2910, 2446, 1971, 1944, 1712, 1598, 1558, 1538, 1435, 1106, 1035, 1014; HRMS (ES^+) *m/z*; $[M+H]^+$ requires 373.1158 for $C_{20}H_{16}F_3N_2O_2$, found: 373.1157.

3'-Fluoro-5-(3-trifluoromethylphenyl)-[2,4']bipyridinyl-3-carboxylic acid ethyl ester (8o)

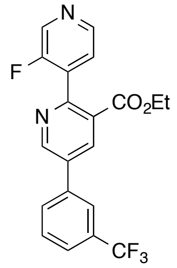

Prepared according to general procedure B using 3-trifluoromethylcinnamic acid (0.21 g, 1.0 mmol), 3-fluoroisonicotinaldehyde (0.16 ml, 1.1 mmol) and ethyl (*E*)-3-(pyrrolidin-1-yl)-2-propenoate (0.34 g, 2.0 mmol). Purification by column chromatography (SiO_2, 10-100% EtOAc in petrol) gave the pyridine **8k** as yellow oil (0.21 g, 52%). δ_H (300 MHz, $CDCl_3$); 9.08 (1H, d, *J* 2.2 Hz, pyr-H), 8.59 (1H, br d, *J* 3.8 Hz, pyr-H), 8.56-8.50 (2H, m, pyr-H), 7.92 (1H, s, ArH), 7.88 (1H, d, *J* 7.7 Hz, ArH), 7.75 (1H, d, *J* 7.7 Hz, ArH), 7.69 (1H, d, *J* 7.7 Hz, ArH), 7.67-7.58 (1H, m, pyr-H), 4.30 (2H, q, *J* 7.1 Hz, CH_2), 1.19 (3H, t, *J* 7.1 Hz, CH_2CH_3); δ_C (75 MHz, $CDCl_3$); 165.5 (C=O), 154.8 (d, *J* 257.6 Hz, CF, quat.), 150.6 (quat.), 150.3 (ArCH), 146.0 (d, *J* 146.0 Hz, pyr-CH), 137.7 (d, *J* 24.9 Hz, pyr-CH), 136.8 (quat.), 136.7 (ArCH), 135.6 (d, *J* 12.7 Hz, quat.), 135.2 (quat.), 131.8 (q, *J* 33.2 Hz, CCF_3), 130.6 (d, *J* 1.1 Hz, ArCH), 129.9 (ArCH), 128.0 (d, *J* 1.1 Hz, quat.), 125.7 (q, *J* 3.7 Hz, ArCH), 124.6 (ArCH), 124.1 (q, *J* 3.9 Hz, ArCH), 123.2 (q, *J* 273.7 Hz, CF_3), 62.04 (CH_2), 13.7 (CH_3); ν_{max}/cm^{-1} (solid); 3047, 2985, 1725, 1611, 1564, 1491, 1438, 1414, 1366, 1340,1300, 1277, 1250, 1168, 1128, 1078, 1063, 1017; HRMS (ES^+) *m/z*; $[M+H]^+$ requires 391.1064 for $C_{20}H_{15}F_4N_2O_2$, found: 391.1079.

Ethyl 3'-chloro-5-[3-(trifluoromethyl)phenyl]-2,4'-bipyridine-3-carboxylate (8p)

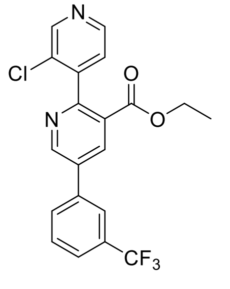

Prepared according to general procedure B using 3-trifluoromethylcinnamic acid (0.21 g, 1.0 mmol), 3-chloro-4-pyridinecarboxaldehyde (0.16 g, 1.1 mmol) and ethyl (*E*)-3-(pyrrolidin-1-yl)-2-propenoate (0.34 g, 2.0 mmol). Purification by column chromatography (SiO_2, 0-50% EtOAc in petrol) gave the pyridine **8p** as yellow needles (0.12 g, 29%). M.p.= 101-102 °C (EtOH); δ_H (300 MHz, $CDCl_3$); 8.98 (1H, d, *J* 2.5 Hz, pyr-H), 8.59 (1H, s, pyr-H), 8.55 (1H, d, *J* 4.9 Hz, pyr-H), 8.51 (1H, d, *J* 2.5 Hz, pyr-H), 7.85 (1H, s, ArH'), 7.80 (1H, d, *J* 7.6 Hz, ArH), 7.69-7.58 (2H, m, ArH), 7.33 (1H, d, *J* 4.9 Hz,

pyr-H), 4.15 (2H, q, *J* 7.1 Hz, CH_2), 1.02 (3H, t, *J* 7.1 Hz, CH_3); δ_C (75 MHz, $CDCl_3$); 165.1 (C=O), 154.2 (quat.), 150.4 (ArCH), 149.0 (ArCH), 147.9 (ArCH), 147.2 (quat.), 136.9 (quat.), 136.8 (ArCH), 135.3 (quat.), 131.8 (q, *J* 33.2 Hz, CCF_3), 130.6 (q, *J* 1.1 Hz, ArCH), 130.3 (quat.), 129.9 (ArCH), 127.2 (quat.), 125.7 (q, *J* 3.3 Hz, ArCH), 124.3 (ArCH), 124.1 (q, *J* 3.3 Hz, ArCH), 120.2 (q, *J* 273.1 Hz, CF_3), 62.1 (CH_2), 13.5 (CH_3); ν_{max}/cm^{-1} (solid); 2970, 1738, 1590, 1554, 1365, 1217, 1116, 1025; HRMS (ES^+) *m/z*; $[M+H]^+$ requires 407.0769 for $C_{20}H_{15}{}^{35}ClF_3N_2O_2$, found: 407.0783.

Ethyl 3',5'-dibromo-5-[3-(trifluoromethyl)phenyl]-2,4'-bipyridine-3-carboxylate (8q)

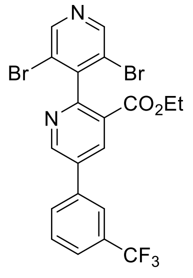

Prepared according to general procedure B using 3-trifluoromethylcinnamic acid (0.21 g, 1.0 mmol), 3,5-dibromo-4-pyridinecarboxaldehyde (0.29 g, 1.1 mmol) and ethyl (*E*)-3-(pyrrolidin-1-yl)-2-propenoate (0.34 g, 2.0 mmol). Purification by column chromatography (SiO_2, 0-30% EtOAc in petrol) gave the pyridine **8q** as yellow needles (0.17 g, 32%). M.p.= 87-88°C (EtOH); δ_H (300 MHz, $CDCl_3$); 9.04 (1H, d, *J* 2.2 Hz, pyr-H), 8.68 (2H, s, pyr-H), 8.61 (1H, d, *J* 2.2 Hz, pyr-H), 7.89 (1H, s, ArH), 7.84 (1H, d, *J* 7.7 Hz, ArH), 7.70-7.59 (2H, m, ArH), 4.16 (2H, q, *J* 7.1 Hz, CH_2), 1.03 (3H, t, *J* 7.1 Hz, CH_3); δ_C (75 MHz, $CDCl_3$); 164.1 (C=O), 155.5 (quat.), 151.0 (ArCH), 150.0 ArCH), 149.7 (quat.), 137.3 (ArCH), 136.8 (quat.), 135.6 (quat.), 131.9 (q, *J* 33.7 Hz, CCF_3), 130.6 (ArCH), 129.9 (ArCH), 125.9 (quat.), 125.7 (ArCH), 124.2 (ArCH), 122.0 (q, *J* 274.2 Hz, CF_3), 120.8 (quat.), 62.1 (CH_2), 13.6 (CH_3) ; ν_{max}/cm^{-1} (solid); 3005, 1716, 1425, 1365, 1225, 1093; HRMS (ES^+) *m/z*; $[M+H]^+$ requires 528.9369 for $C_{20}H_{14}{}^{79}Br_2F_3N_2O_2$, found: 528.9360.

5-(3-Trifluoromethylphenyl)-[2,4']bipyridinyl-3-carboxylic acid ethyl ester (8r)

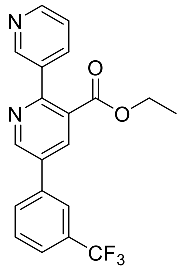

Prepared according to general procedure B using 3-trifluoromethylcinnamic acid (0.21 g, 1.0 mmol), 3-pyridinecarboxaldehyde (0.1 ml, 1.1 mmol) and ethyl (*E*)-3-(pyrrolidin-1-yl)-2-propenoate (0.34 g, 2.0 mmol). Purification by column chromatography (SiO_2, 20-100% EtOAc in petrol) gave the pyridine **8r** as yellow needles (0.081 g, 32%). M.p.= 87-88 °C (EtOAc–Petrol); δ_H (300 MHz, $CDCl_3$); 9.04 (1H, d, *J* 2.7 Hz, pyr-H), 8.79 (1H, d, *J* 1.6 Hz, pyr-H), 8.68 (1H, d, *J* 4.9, 1.6 Hz, pyr-H), 8.42 (1H, d, *J* 2.7 Hz, pyr-H), 7.96 (1H, dt, *J* 8.0, 1.6 Hz, pyr-H), 7.91 (1H, s, ArH), 7.86 (1H, d, *J* 7.7 Hz, ArH), 7.74 (1H, d, *J* 7.7 Hz, ArH), 7.68 (1H, t, *J* 7.7 Hz, ArH), 7.43 (1H, ddd, *J* 7.7, 4.9, 1.6 Hz, pyr-H), 4.25 (2H, q, *J* 7.1 Hz, CH_2), 1.14 (3H, t, *J* 7.1 Hz, CH_2CH_3); δ_C (75 MHz, $CDCl_3$); 167.0 (C=O), 155.4 (quat.), 149.8 (ArCH), 149.6 (ArCH), 149.5 (ArCH), 137.1 (quat.), 136.8 (ArCH), 136.1 (ArCH), 135.6 (quat.), 134.1 (quat.), 131.8 (q, *J* 32.6 Hz, CCF_3), 130.5 (ArCH), 129.9 (ArCH), 127.4 (q, *J* 271.5 Hz, CF_3), 127.2 (quat.), 125.5 (q, *J* 3.9 Hz, ArCH), 124.0 (q, *J* 3.9 Hz, ArCH), 123.0 (ArCH), 62.0 (CH_2), 13.8 (CH_3); ν_{max}/cm^{-1} (solid); 2981, 1709, 1590, 1445, 1416, 1253, 1174, 1116, 1012; HRMS (ES^+) *m/z*; $[M+H]^+$ requires 373.1158 for $C_{20}H_{16}F_3N_2O_2$, found: 373.1167.

Ethyl 6'-methoxy-5-[3-(trifluoromethyl)phenyl]-2,3'-bipyridine-3-carboxylate (8t)

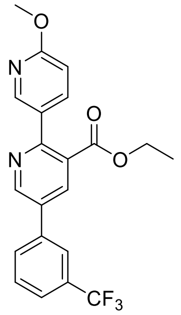

Prepared according to general procedure B using 3-trifluoromethylcinnamic acid (0.21 g, 1.0 mmol), 6-methoxy-3-pyridinecarboxaldehyde (0.15 g, 1.1 mmol) and ethyl (*E*)-3-(pyrrolidin-1-yl)-2-propenoate (0.34 g, 2.0 mmol). Purification by column chromatography (SiO_2, 0-100% EtOAc in petrol) gave the pyridine **8t** as yellow needles (0.045 g, 12%). M.p.= 112-113°C (EtOH); δ_H (300 MHz, $CDCl_3$); 8.92 (1H, d, *J* 2.5 Hz, pyr-H), 8.30 (1H, d, *J* 2.5 Hz, pyr-H), 8.26 (1H, d, *J* 2.5 Hz, pyr-H), 7.82-7.75 (3H, m, pyr-H + ArH), 7.65-7.55 (2H, m, ArH), 6.77 (1H, d, *J* 8.8 Hz, pyr-H), 4.21 (2H, q, *J* 7.0 Hz, CH_2), 3.93 (3H, s, OCH_3), 1.13 (3H, t, *J* 7.0 Hz, CH_3); δ_C (75 MHz, $CDCl_3$); 167.5 (C=O), 164.4 (quat.) 155.2 (quat.), 149.6 (ArCH), 147.1 (ArCH), 139.1 (ArCH), 137.3 (quat.), 136.7 (ArCH), 133.4 (quat.), 131.8 (q, *J* 32.1 Hz, CCF_3), 130.4 (q, *J* 1.1 Hz, ArCH), 129.8 (ArCH), 128.6 (quat.), 126.9 (quat.), 125.3 (q, *J* 3.9 Hz, ArCH), 124.4 (q, *J* 272.6 Hz, CF_3), 123.9 (q, *J* 3.9 Hz, ArCH), 110.4 (ArCH), 61.9 (CH_2), 53.8 (OCH_3), 13.9 (CH_3) ; ν_{max}/cm^{-1} (solid); 3604, 3415, 2929, 2576, 2441, 2263, 2145, 2000, 1764, 1216, 1092; HRMS (ES^+) *m/z*; $[M+H]^+$ requires 403.1264 for $C_{21}H_{18}F_3N_2O_3$, found: 403.1260.

2-(4-Methyl-2-phenylpyrimidin-5-yl)-5-(3-trifluoromethylphenyl)nicotinic acid ethyl ester (8u)

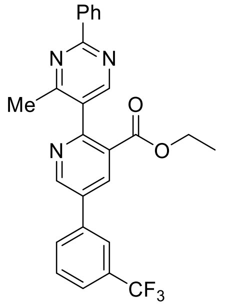

Prepared according to general procedure B using 3-trifluoromethylcinnamic acid (0.21 g, 1.0 mmol), 4-methyl-2-phenyl-5-pyrimidylcarboxaldehyde (0.22 g, 1.1 mmol) and ethyl (*E*)-3-(pyrrolidin-1-yl)-2-propenoate (0.34 g, 2.0 mmol). Purification by column chromatography (SiO_2, 10-100% EtOAc in petrol) gave the pyridine **8u** as yellow needles (0.18 g, 39%). M.p.= 99-100 °C (EtOH); δ_H (300 MHz, $CDCl_3$); 9.09 (1H, d, *J* 2.2 Hz, pyr-H), 8.61 (1H, s, ArH), 8.59 (1H, d, *J* 2.2 Hz, pyr-H), 8.54-8.49 (2H, m, ArH), 7.94 (1H, s, ArH), 7.88 (1H, d, *J* 7.7 Hz, ArH), 7.75 (1H, d, *J* 7.7 Hz, ArH), 7.69 (1H, t, *J* 7.7 Hz, ArH), 7.53-7.48 (3H, m, ArH), 4.21 (2H, q, *J* 7.1 Hz, CH_2), 2.48 (3H, s, CH_3), 1.16 (3H, t, *J* 7.1 Hz, CH_2CH_3); δ_C (75 MHz, $CDCl_3$); 165.6 (quat.), 164.7 (quat.), 163.7 (quat.), 155.8 (ArCH), 155.0 (quat.), 150.4 (ArCH), 137.6 (quat.), 137.1 (ArCH), 137.0 (quat.), 134.7 (quat.), 131.8 (q, *J* 33.1 Hz, CCF_3), 131.5 (quat.), 130.7 (ArCH), 130.6 (ArCH), 130.0 (ArCH), 128.6 (ArCH), 128.3 (ArCH), 127.4 (quat.), 125.6 (q, *J* 3.3 Hz, ArCH), 124.5 (q, *J* 272.0 Hz, CF_3), 124.1 (q, *J* 3.3 Hz, ArCH), 62.1 (CH_2), 22.9 (CH_3), 13.9 (CH_2CH_3); ν_{max}/cm^{-1} (solid); 2965, 1723, 1572, 1532, 1422, 1338, 1252, 1168, 1120, 1019; HRMS (ES^+) *m/z*; $[M+H]^+$ requires 464.1580 for $C_{26}H_{21}F_3N_3O_2$, found: 464.1581.

Ethyl 2-(quinolin-4-yl)-5-[3-(trifluoromethyl)phenyl]pyridine-3-carboxylate (8v)

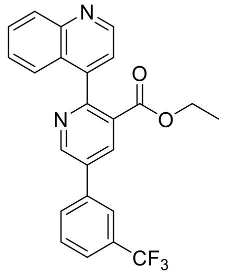

Prepared according to general procedure B using 3-trifluoromethylcinnamic acid (0.21 g, 1.0 mmol), 4-quinolinecarboxaldehyde (0.17 g, 1.1 mmol) and ethyl (*E*)-3-(pyrrolidin-1-yl)-2-propenoate (0.34 g, 2.0 mmol). Purification by column chromatography (SiO_2, 10-100% EtOAc in petrol) gave the pyridine **8v** as yellow needles (0.16 g, 38%). M.p.= 127-128°C (EtOH); δ_H (300 MHz, $CDCl_3$); 9.14 (1H, d, *J* 2.2 Hz, pyr-H), 9.04 (1H, d, *J* 4.4 Hz, ArH), 8.61 (1H, d, *J* 2.2 Hz, pyr-H), 8.21 (1H, d, *J* 8.2 Hz, ArH), 7.98 (1H, s, ArH), 7.92 (1H, d, *J* 7.7 Hz, ArH), 7.79-7.68 (3H, m, ArH), 7.57-7.47 (2H, m, ArH), 7.43 (1H, d, *J* 4.4 Hz, ArH), 3.90 (2H, q, *J* 7.1 Hz, CH_2), 0.61 (3H, t, *J* 7.1 Hz, CH_3); δ_C (75 MHz, $CDCl_3$); 165.7 (C=O), 155.7 (quat.) 150.3 (ArCH), 149.8 (ArCH), 148.1 (quat.), 146.7 (quat.), 137.0 (quat.), 136.8 (ArCH), 134.9 (quat.), 131.9 (q, *J* 33.2 Hz, *C*CF_3), 130.6 (d, *J* 1.7 Hz, ArCH), 130.1 (ArCH), 129.9 (ArCH), 129.4 (ArCH), 128.1 (quat.), 127.1 (ArCH), 126.6 (quat.), 125.5 (q, *J* 3.9 Hz, ArCH), 124.8 (ArCH), 124.1 (q, *J* 3.9 Hz, ArCH), 123.3 (q, *J* 274.2 Hz, CF_3), 120.6 (ArCH), 61.7 (CH_2), 12.9 (CH_3); ν_{max}/cm^{-1} (solid); 3063, 2984, 1715, 1591, 1509, 1385, 1123, 1018; HRMS (ES^+) *m/z*; $[M+H]^+$ requires 423.1315 for $C_{24}H_{18}F_3N_2O_2$, found: 423.1332.

Ethyl 2-(quinolin-3-yl)-5-[3-(trifluoromethyl)phenyl]pyridine-3-carboxylate (8w)

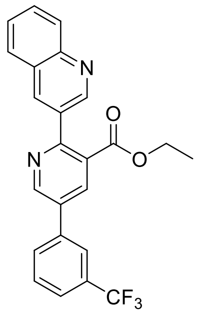

Prepared according to general procedure B using 3-trifluoromethylcinnamic acid (0.21 g, 1.0 mmol), 3-quinolinecarboxaldehyde (0.17 g, 1.1 mmol) and ethyl (*E*)-3-(pyrrolidin-1-yl)-2-propenoate (0.34 g, 2.0 mmol). Purification by column chromatography (SiO_2, 20-100% EtOAc in petrol) gave the pyridine **8w** as yellow needles (0.07 g, 16%). M.p.= 120-121 °C (EtOH); δ_H (300 MHz, $CDCl_3$); 9.09 (2H, m, pyr-H + ArH), 8.47 (2H, m, pyr-H + ArH), 8.18 (1H, d, *J* 8.5 Hz, ArH), 7.94-7.87 (3H, m, ArH), 7.82-7.59 (4H, m, ArH), 4.25 (2H, q, *J* 7.2 Hz, CH_2), 1.07 (3H, t, *J* 7.2 Hz, CH_3); δ_C (75 MHz, $CDCl_3$); 167.1 (C=O), 155.4 (quat.), 150.4 (ArCH), 150.0 (ArCH), 147.8 (quat.), 137.2 (quat.), 136.9 (ArCH), 135.8 (ArCH), 134.1 (quat.), 132.7 (quat.), 131.6 (q, *J* 33.2 Hz, CCF_3), 130.5 (q, *J* 1.1 Hz, ArCH), 130.2 (ArCH), 129.9 (ArCH), 129.3 (ArCH), 128.4 (ArCH), 127.4 (quat.), 127.3 (quat.), 127.1 (ArCH), 125.5 (q, *J* 3.9 Hz, ArCH), 125.4 (q, *J* 272.6 Hz, CF_3), 124.0 (q, *J* 3.9 Hz, ArCH), 62.1 (CH_2), 13.8 (CH_3); ν_{max}/cm^{-1} (solid); 2990, 1730, 1569, 1549, 1438, 1341, 1298, 1276, 1235, 1209, 1087; HRMS (ES^+) *m/z*; $[M+H]^+$ requires 423.1315 for $C_{24}H_{18}F_3N_2O_2$, found: 423.1320.

Ethyl 2,1':5',1''-terpyridine-3-carboxylate (8x)

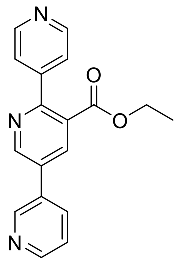

Prepared according to general procedure B using *trans*-3-(3-pyridyl)acrylic acid (0.30 g, 2.0 mmol), 4-pyridinecarboxaldehyde (0.2 ml, 2.0 mmol) and ethyl (*E*)-3-(pyrrolidin-1-yl)-2-propenoate (0.34 g, 2.0 mmol). Purification by column chromatography (SiO_2, 20-75% EtOAc in petrol) gave the pyridine **8x** as orange needles (0.29 g, 48%). M.p.= 119-120°C (EtOH); δ_H (300 MHz, $CDCl_3$); 8.97 (1H, d, *J* 2.5 Hz, pyr-H), 8.88 (1H, br s, pyr-H), 8.68 (3H, br s, pyr-H), 8.34 (1H, d, *J* 2.5 Hz, pyr-H), 7.92 (1H, m, pyr-H), 7.50-7.40 (3H, m, pyr-H), 4.16 (2H, q, *J* 7.1 Hz, CH_2), 1.06 (3H, t, *J* 7.1 Hz, CH_3); δ_C (75 MHz,

$CDCl_3$); 166.7 (C=O), 155.7 (quat.), 150.1 (quat.), 149.7 (ArCH), 149.2 (ArCH), 148.2 (ArCH), 147.8 (quat.), 136.6 (2xArCH), 134.5 (ArCH), 132.9 (quat.), 127.4 (quat.), 124.0 (ArCH), 123.4 (ArCH), 62.1 (CH_2), 13.6 (CH_3); ν_{max}/cm^{-1} (solid); 2995, 1736, 1422, 1364, 1217, 1101, 1025; HRMS (ES^+) *m/z*; $[M+H]^+$ requires 306.1237 for $C_{18}H_{16}N_3O_2$, found: 306.1249.

Ethyl 5,6-diphenyl-2,4'-bipyridine-3-carboxylate (8y)

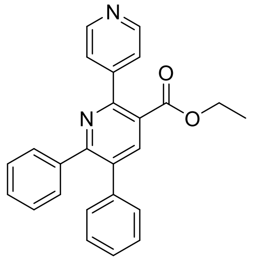

Prepared according to general procedure B using α-phenylcinnamic acid (0.22 g, 1.0 mmol), 4-pyridinecarboxaldehyde (0.1 ml, 1.1 mmol) and ethyl (*E*)-3-(pyrrolidin-1-yl)-2-propenoate (0.34 g, 2.0 mmol). Purification by column chromatography (SiO_2, 0-40% EtOAc in petrol) gave the pyridine **8y** as yellow needles (0.07 g, 18%). M.p.= 174-175 °C (EtOH); δ_H (300 MHz, $CDCl_3$); 8.65 (2H, br s, pyr-H), 8.17 (1H, s, pyr-H), 7.51 (2H, d, *J* 4.1 Hz, pyr-H), 7.38-7.35 (2H, m, ArH), 7.27-7.15 (8H, m, ArH), 4.16 (2H, q, *J* 7.1 Hz, CH_2), 1.06 (3H, t, *J* 7.1 Hz, CH_3); δ_C (75 MHz, $CDCl_3$); 166.9 (C=O), 158.8 (quat.), 154.7 (quat.), 149.2 (ArCH), 148.1 (quat.), 140.9 (ArCH), 138.8 (quat.), 138.4 (quat.), 135.3 (quat.), 130.1 (ArCH), 129.4 (ArCH), 128.7 (ArCH), 128.6 (ArCH), 128.0 (ArCH), 127.9 (ArCH), 125.2 (quat.), 123.7 (ArCH), 61.8 (CH_2), 13.7 (CH_3); ν_{max}/cm^{-1} (solid); 3004, 2970, 1737, 1436, 1365, 1228, 1217; HRMS (ES^+) *m/z*; $[M+H]^+$ requires 381.1598 for $C_{25}H_{21}N_2O_2$, found: 381.1594.

Ethyl 6-methyl-5-phenyl-2,4'-bipyridine-3-carboxylate (8z)

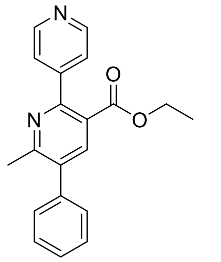

Prepared according to general procedure B using α-methylcinnamic acid (0.16 g, 1.0 mmol), 4-pyridinecarboxaldehyde (0.1 ml, 1.1 mmol) and ethyl (*E*)-3-(pyrrolidin-1-yl)-2-propenoate (0.34 g, 2.0 mmol). Purification by column chromatography (SiO_2, 50-100% EtOAc in hexane) gave the pyridine **8z** as yellow oil (0.088 g, 27%). δ_H (300 MHz, $CDCl_3$); 8.64 (2H, d, *J* 5.5 Hz, pyr-H), 8.01 (1H, s, pyr-H), 7.46-7.37 (5H, m, ArH), 7.33-7.30 (2H, m, ArH), 4.11 (2H, q, *J* 7.1 Hz, CH_2), 2.54 (3H, s, CH_3) 1.01 (3H, t, *J* 7.1 Hz, CH_2CH_3); δ_C (75 MHz, $CDCl_3$); 166.9 (C=O), 159.0 (quat.), 154.7 (quat.), 149.3 (ArCH), 148.2 (quat.), 139.4 (ArCH), 138.2 (quat.), 136.4 (quat.), 129.0 (ArCH), 128.7 (ArCH), 128.1 (ArCH), 124.4 (quat.), 123.5 (ArCH), 61.7 (CH_2), 23.7 (CH_3), 13.7 (CH_2CH_3); ν_{max}/cm^{-1} (solid); 3032, 2982, 1722, 1599, 1537, 1427, 1388, 1254, 1113, 1057, 1015; HRMS (ES^+) *m/z*; $[M+H]^+$ requires 319.1441 for $C_{20}H_{19}N_2O_2$, found: 319.1454.

Ethyl 6-(*tert*-butyl-2-pyridin-4-yl)-5,6,7,8-tetrahydroquinoline-3-carboxylate (8aa)

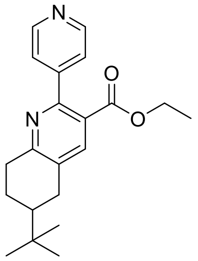

Prepared according to general procedure A using 1-cyclohexenyl carboxylic acid (0.18 g, 1.0 mmol), 4-pyridinecarboxaldehyde (0.1 ml, 1.1 mmol) and ethyl (*E*)-3-(pyrrolidin-1-yl)-2-propenoate (0.34 g, 2.0 mmol). Purification by column chromatography (SiO_2, 0-100% EtOAc in hexane) gave the pyridine **8aa** as yellow plates (0.067 g, 10%). M.p. = 89-90 °C ($CHCl_3$); δ_H (500 MHz, $CDCl_3$); 8.58 (2H, d, *J* 4.1 Hz, pyr-H), 7.86 (1H, s, pyr-H), 7.32 (2H, d, *J* 4.1 Hz, pyr-H), 4.07 (2H, q, *J* 7.4 Hz, CH_2CH_3), 3.08 (1H, ddd, *J* 17.6, 4.9, 1.6 Hz, CH_{eq}), 2.86 (2H, m, CH_{eq} + CH_{ax}), 2.56 (1H, dd, *J* 16.6, 11.1 Hz, CH_{ax}), 2.10-2.05 (1H, m, CH_{ax} or CH_{eq}), 1.52-1.37 (2H, m, CH + CH_{ax} or CH_{eq}), 1.00 (3H, t, *J* 7.4 Hz, CH_2CH_3), 0.92 (9H, s, ^{t}Bu); δ_C (125 MHz, $CDCl_3$); 167.1 (C=O), 160.7 (quat.), 153.8 (quat.), 149.4 (ArCH), 148.6 (quat.), 139.3 (ArCH), 132.3 (quat.), 138.4 (quat.), 124.0 (quat.), 123.4 (ArCH), 61.4 (CH_2CH_3), 44.2 (CH), 33.7 (CH_2), 29.9 (CH_2), 27.6 (CH_3), 24.2 (CH_2), 13.6 (CH_2CH_3); ν_{max}/cm^{-1} (solid); 2956, 2866, 1712, 1596, 1543, 1411, 1365, 1291, 1215, 1094, 1068, 1023; HRMS (ES^+) *m/z*; $[M+H]^+$ requires 339.2067 for $C_{21}H_{27}N_2O_2$, found: 339.2080.

3-Tosyl-5-(3-trifluoromethylphenyl)-2,4′-bipyridine (8ab)

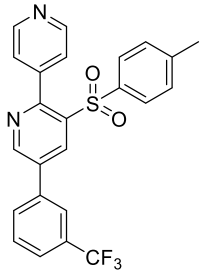

Prepared according to general procedure B using 3-trifluoromethylcinnamic acid (0.21 g, 1.0 mmol), 4-pyridinecarboxaldehyde (0.1 mL, 1.1 mmol) and 1-(*E*)-2-[(4-methylphenyl)sulfonyl]ethenyl pyrrolidine (0.50 g, 2.0 mmol). Purification by column chromatography (SiO_2, 30-100% EtOAc in hexane then 0-100% EtOAc in CH_2Cl_2) gave the pyridine **8ab** as brown plates (0.087 g, 19%). M.p. = 145-146 °C ($CHCl_3$); δ_H (300 MHz, $CDCl_3$); 8.98 (1H, d, *J* 2.2 Hz, pyr-H), 8.84 (1H, d, *J* 2.2 Hz, pyr-H), 8.52 (2H, br s, pyr-H), 7.88 (1H, s, ArH), 7.84 (1H, d, *J* 8.0 Hz, ArH), 7.73 (1H, d, *J* 8.0 Hz, ArH), 7.65 (1H, t, *J* 8.0 Hz, ArH), 7.17-7.14 (4H, m, $SO_2C_6H_4$), 7.03 (2H, d, *J* 8.2 Hz, pyr-H), 2.30 (3H, s, CH_3); δ_C (75 MHz, $CDCl_3$); 154.9 (quat.), 150.7 (ArCH), 148.7 (ArCH) 145.9 (quat.), 145.1 (quat.), 138.0 (quat.), 136.2 (d, *J* 4.2 Hz, quat.), 135.4 (quat.), 135.2 (ArCH), 132.2 (q, *J* 33.2 Hz, CCF_3), 130.7(ArCH), 130.1 (ArCH),129.6 (ArCH), 128.0 (ArCH), 126.1 (q, *J* 4.2 Hz, ArCH), 124.5 (ArCH), 124.2 (q, *J* 4.2 Hz, ArCH), 124.0 (q, *J* 274.0 Hz, CF_3), 122.8 (quat.), 21.6 (CH_3); ν_{max}/cm^{-1} (solid); 3047, 1596, 1532, 1438, 1320, 1219, 1149, 1116, 1080, 1049; HRMS (ES^+) *m/z*; $[M+H]^+$ requires 455.1040 for $C_{24}H_{18}F_3N_2O_2S$, found: 455.1036.

1-(5-(3-Trifluoromethylphenyl)-[2,4′-bipyridin]-3-yl) ethanone (8ac)

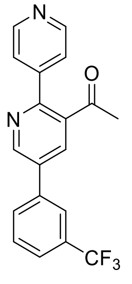

Prepared according to general procedure B using 3-trifluoromethylcinnamic acid (0.21 g, 1.0 mmol), 4-pyridinecarboxaldehyde (0.1 mL, 1.1 mmol) and ethyl (*E*)-3-(pyrrolidin-1-yl)-2-propenoate (0.34 g, 2.0 mmol). Purification by column chromatography (SiO_2, 50-100% EtOAc–hexane) gave the pyridine **8ac** as orange needles (0.14 g, 41%). M.p.= 113-115°C (EtOH); δ_H (300 MHz, $CDCl_3$); 8.96 (1H, d, *J* 2.2 Hz, pyr-H), 8.70 (2H, d, *J* 5.5 Hz, pyr-H), 8.02 (1H, d, *J* 2.2 Hz, pyr-H), 7.82 (1H, s, ArH), 7.77 (1H, d, *J* 7.7 Hz, ArH), 7.67 (1H, d, *J* 7.7 Hz, ArH), 7.60 (1H, m, ArH), 7.48 (2H, d, *J* 5.5 Hz, pyr-H), 2.21 (3H,

s, CH_3); δ_C (75 MHz, $CDCl_3$); 201.2 (C=O), 152.7 (quat.), 149.7 (ArCH), 148.3 (ArCH), 145.5 (quat.), 136.0 (quat.), 135.4 (quat.), 133.8 (quat.), 133.7 (ArCH), 131.1 (q, *J* 32.1 Hz, *C*CF_3), 129.5 (ArCH), 128.9 (ArCH), 124.6 (q, *J* 3.9 Hz, ArCH), 123.0 (q, *J* 3.9 Hz, ArCH), 122.9 (q, *J* 243.3 Hz, CF_3), 122.5 (ArCH), 29.6 (CH_3); ν_{max}/cm^{-1} (solid); 3054, 2961, 1692, 1596, 1533, 1433, 1342, 1296, 1161, 1099, 1076, 1052; HRMS (ES^+) *m/z*; $[M+H]^+$ requires 343.1053 for $C_{19}H_{14}F_3N_2O$, found: 343.1056.

Ethyl 4-methyl-5-(3-trifluoromethylphenyl)-[2,4′-bipyridine]3-carboxylate 159c and 3,5-di(3-trifluoromethylphenyl)-2-(4-pyridyl)pyridine (8ad)

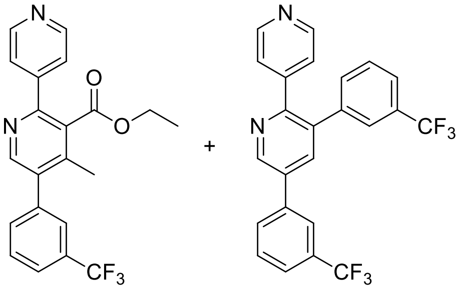

Prepared according to general procedure B using 3-trifluoromethylcinnamic acid (0.21 g, 1.0 mmol), 4-pyridinecarboxaldehyde (0.1 mL, 1.1 mmol) and ethyl trans-2-methyl-3-(1-pyrrolidinyl)acrylate (0.37 g, 2.0 mmol). Purification by column chromatography (SiO_2, 20-100% EtOAc in hexane then 0-100% EtOAc in CH_2Cl_2) gave a 5:1 inseparable mixture of pyridine **8ad** (major) and pyridine **9b** (minor) as yellow oil (0.15 g, 30% yield **8ad**). ν_{max}/cm^{-1} (solid); 3036, 2984, 1727, 1598, 1538, 1436, 1379, 1340, 1249, 1167, 1127, 1097, 1071, 1020.

Ethyl 4-methyl-5-(3-trifluoromethylphenyl)-[2,4′-bipyridine]3-carboxylate (8ad)

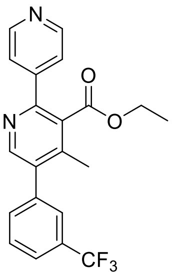

δ_H (300 MHz, $CDCl_3$); 8.66 (2H, d, *J* 4.7 Hz, pyr-H), 8.53 (1H, s, pyr-H), 7.66 (1H, d, *J* 7.7 Hz, ArH), 7.58-7.56 (2H, m, ArH), 7.52-7.50 (3H, m, pyr-H + ArH), 4.14 (2H, q, *J* 7.6 Hz, C*H*$_2$$CH_3$), 2.25 (3H, s, Ar$CH_3$), 1.03 (3H, t, *J* 7.6 Hz, CH_2C*H*$_3$); δ_C (125 MHz, $CDCl_3$) 18 of 19 signals observed; 168.0 (C=O), 153.1 (quat.), 150.4 (ArCH), 150.1 (ArCH), 147.2 (quat.), 143.3 (quat.), 136.8 (quat.), 132.7 (q, *J* 1.7 Hz, quat.), 131.3 (q, *J* 33.5 Hz, *C*CF_3), 130.4 (quat.), 129.7 (ArCH), 129.4 (ArCH), 126.1 (q, *J* 3.9 Hz, ArCH), 125.2 (q, *J* 3.9 Hz, ArCH), 122.8 (ArCH), 62.1 (CH_2), 17.3 (ArCH_3), 13.6 (CH_3); HRMS (ES^+) *m/z*; $[M+H]^+$ requires 387.1315 for $C_{21}H_{18}F_3N_2O_2$, found: 387.1320.

3,5-Di(3-trifluoromethylphenyl)-2-(4-pyridyl)pyridine (9b)

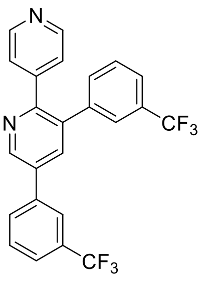

Characteristic signals: δ_H (300 MHz, $CDCl_3$); 8.93 (1H, d, *J* 2.2 Hz, pyr-H), 8.49 (2H, d, *J* 5.7 Hz, pyr-H), 7.90 (1H, d, *J* 2.2 Hz, pyr-H), 7.84 (1H, s, ArH), 7.81 (1H, d, *J* 7.2 Hz, ArH), 7.47 (2H, m, ArH), 7.40 (1H, t, *J* 8.5 Hz, ArH), 7.30 (1H, d, *J* 7.2 Hz, ArH), 7.24 (2H, d, *J* 5.7 Hz, pyr-H); δ_C (125 MHz, $CDCl_3$); 153.7 (quat.), 149.5 (ArCH), 147.9 (ArCH), 139.3 (quat.), 137.6 (quat.), 137.1 (ArCH), 135.2 (quat.), 133.2 (ArCH), 132.9 (q, *J* 1.7 Hz, ArCH), 131.5 (quat.), 131.0 (quat.), 130.6 (ArCH), 129.9 (ArCH), 129.3 (ArCH), 125.5 (q, *J* 3.1 Hz, ArCH), 124.7 (ArCH), 124.1 (q, *J* 3.1 Hz, ArCH); HRMS (ES^+) *m/z*; $[M+H]^+$ requires 445.1134 for $C_{24}H_{15}F_6N_2$, found: 445.1143.

4-Phenyl-1-(4-trifluoromethylphenyl)-6,7-dihydro-*5H*-[2]pyrindine (8ae)

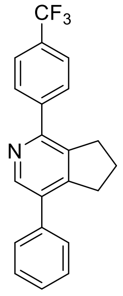

Prepared according to general procedure A using cinnamic acid (0.74 g, 5.0 mmol), DPPA (1.0 mL, 4.5 mmol), triethylamine (0.75 mL, 5.0 mmol), 4-trifluoromethylbenzaldehyde (0.67 mL, 4.95 mmol) and 1-cyclopent-1-enylpyrrolidine (3.3 mL, 22.5 mmol). Purification by column chromatography (SiO_2, 0-50% EtOAc in hexane then high grade SiO_2 with 0-20% EtOAc in hexane) gave the pyridine **8ad** as yellow needles (0.013 g, 5%). δ_H (500 MHz, $CDCl_3$); 8.58 (1H, s, pyr-H), 7.92 (2H, d, *J* 8.1 Hz, ArH), 7.73 (2H, d, *J* 8.1 Hz, ArH), 7.51-7.48 (4H, m, ArH), 7.43-7.40 (1H, m, ArH), 3.17 (2H, t, *J* 7.3 Hz, CH_2), 3.07 (2H, t, *J* 7.4 Hz, CH_2), 2.10 (2H, quin, *J* 7.3 Hz, CH_2); δ_C (100 MHz, $CDCl_3$); 152.8 (quat.), 151.3 (quat.), 147.3 (ArCH), 143.4 (quat.), 137.8 (quat.), 137.5 (quat.) 133.2 (quat.), 130.3 (q, *J* 34.9 Hz, *C*CF_3), 128.7 (ArCH), 128.6 (ArCH), 128.5 (ArCH), 127.8 (ArCH), 125.7 (q, *J* 271.4 Hz, *C*F_3), 125.2 (q, *J* 4.6 Hz, ArCH), 32.9 (CH_2), 32.8 (CH_2), 25.8 (CH_2); ν_{max}/cm^{-1} (solid); 2950, 1615, 1579, 1497, 1447, 1411, 1373, 1325, 1209, 1165, 1114, 1065, 1014; HRMS (ES^+) *m/z*; $[M+H]^+$ requires 340.1308 for $C_{21}H_{17}F_3N$, found: 340.1315.

References

1. Kanner, C. B.; Pandit, U. K. *Tetrahedron* **1982**, *38*, 3597-3604.

2. Clemo, N. G.; Pattenden, G. *J. Chem. Soc., Perkin Trans. 1* **1986**, 2133-2136.

3. Arjona, O.; Iradier, F.; Medel, R.; Plumet, J. *J. Org. Chem.* **1999**, *64*, 6090-6093.

4. Kozmin, S. A.; Janey, J. M.; Rawal, V. H. *J. Org. Chem.* **1999**, *64*, 3039-3052.

5. Vitnik, V. D.; Ivanovic, M. D.; Vitnik, Z. J.; Dordevic, J. B.; Zizak, Z. S.; Juranic, Z. D.; Juranic, I. O. *Synth. Commun.* **2009**, *39*, 1457-1471.

6. Vyas, D. J.; Frohlich, R.; Oestreich, M. *Org. Lett.* **2011**, *13*, 2094-2097.

7. Toh, K. K.; Wang, Y. F.; Jian Ng, E. P.; Chiba, S. *J. Am. Chem. Soc.* **2011**, *133*, 13942-13945.

^{1}H NMR of **8a** ($CDCl_3$, 300 MHz)

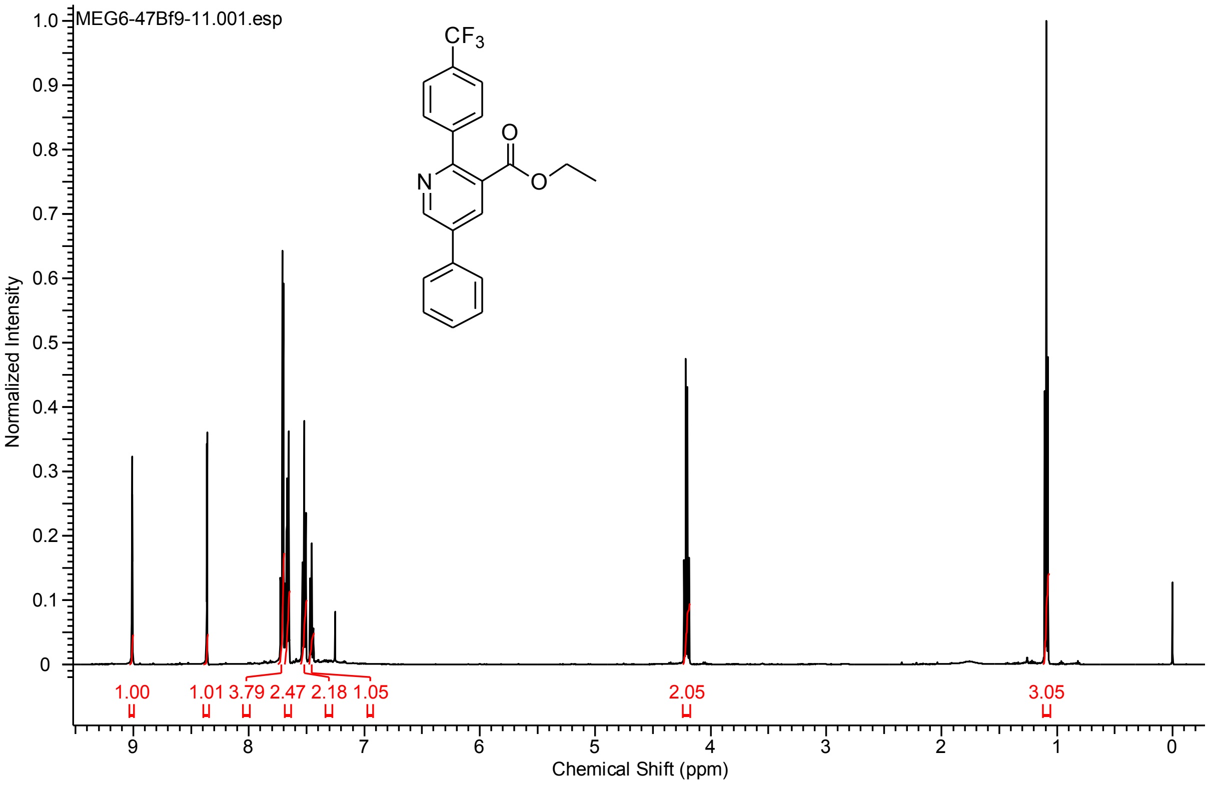

^{13}C NMR of **8a** ($CDCl_3$, 75 MHz)

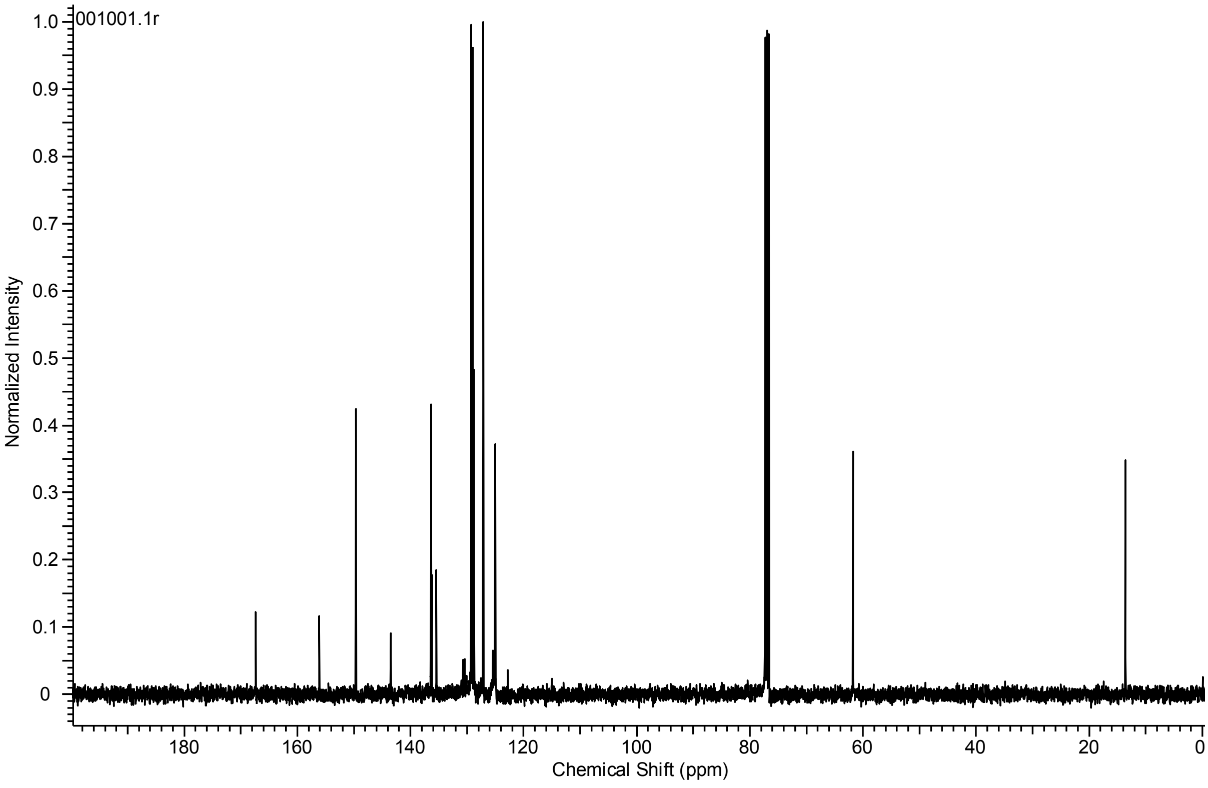

^{1}H NMR of **9** ($CDCl_3$, 300 MHz)

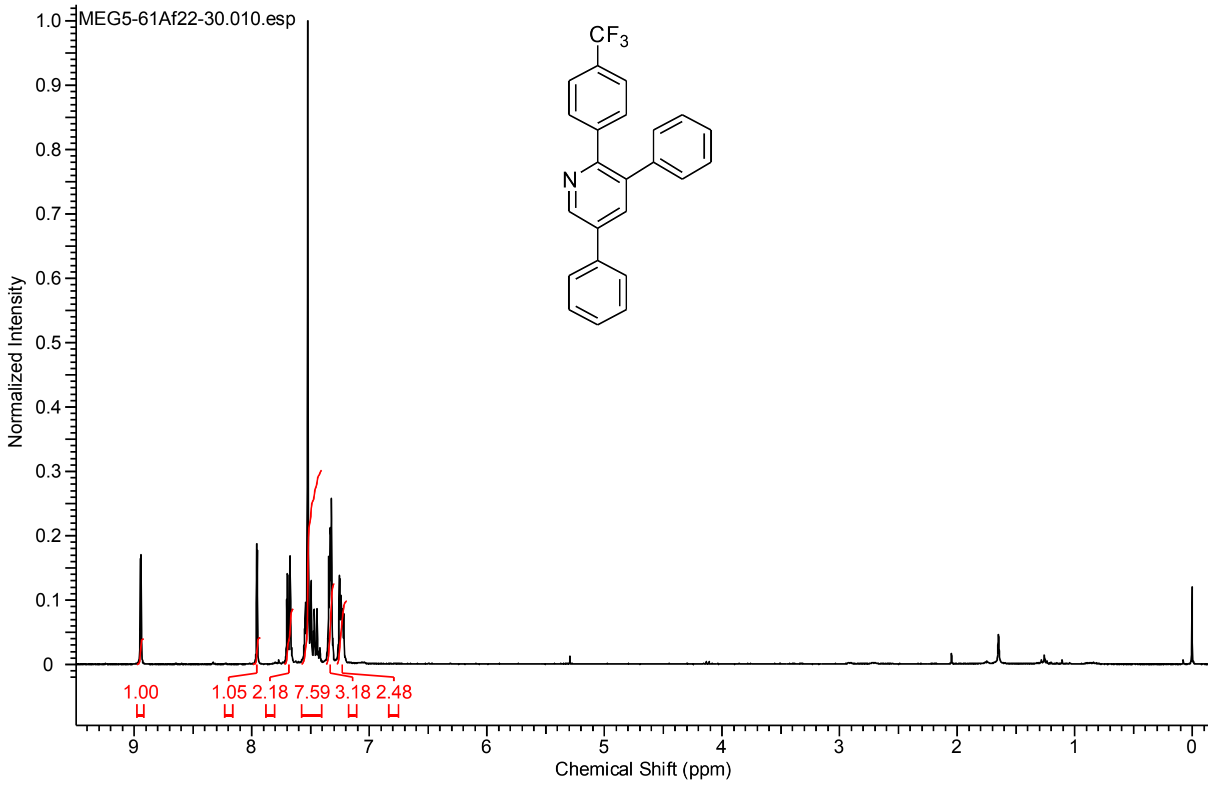

^{13}C NMR of **9** ($CDCl_3$, 75 MHz)

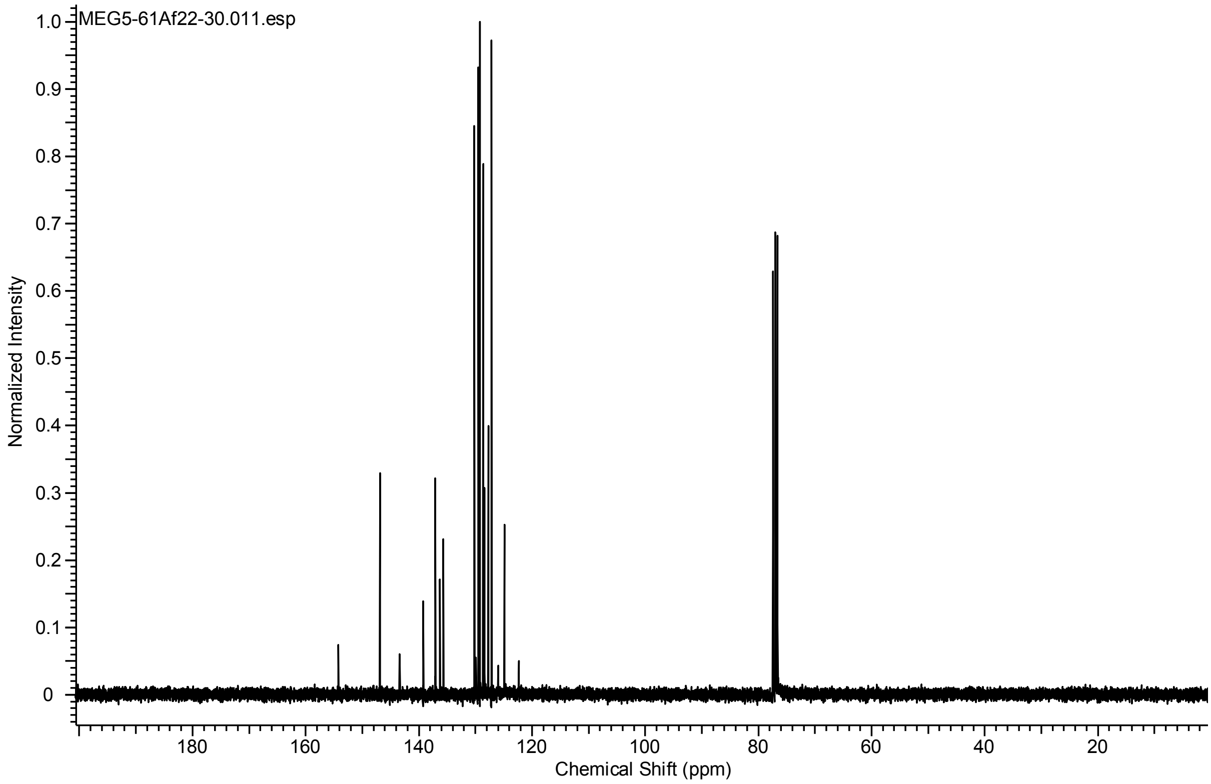

^{1}H NMR of **8b** ($CDCl_3$, 300 MHz)

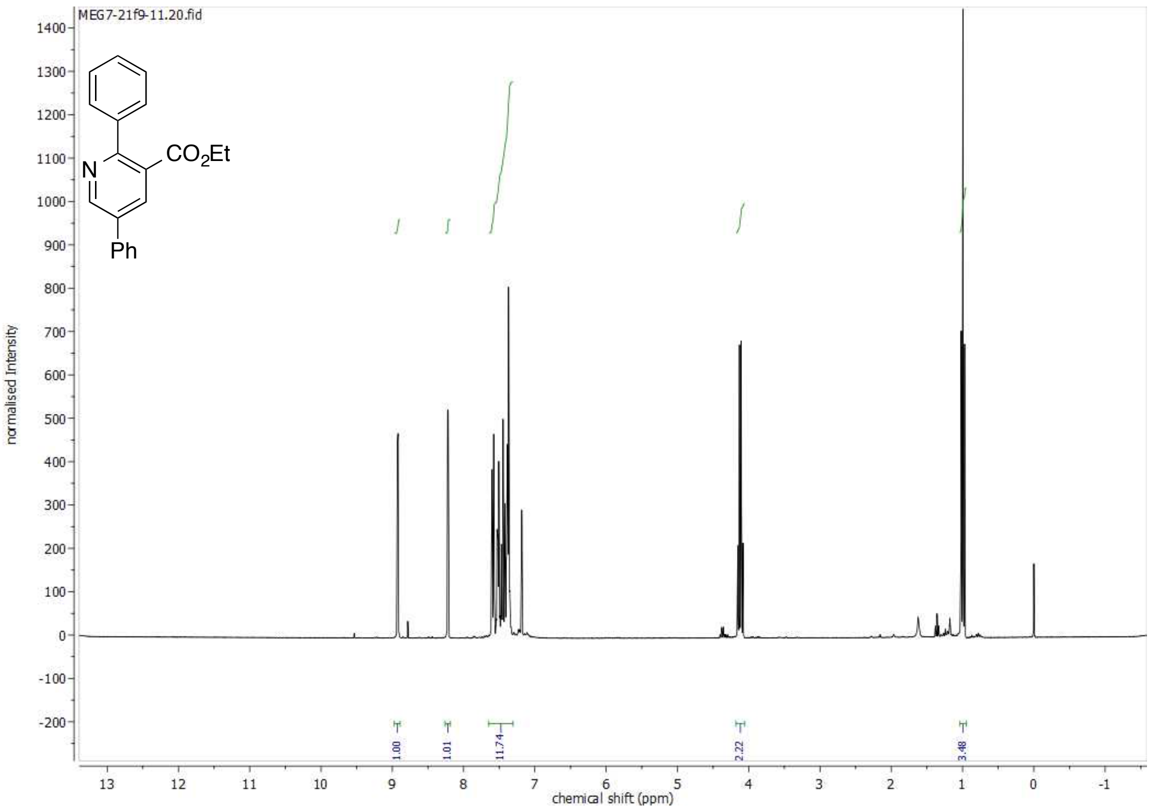

^{13}C NMR of **8b** ($CDCl_3$, 75 MHz)

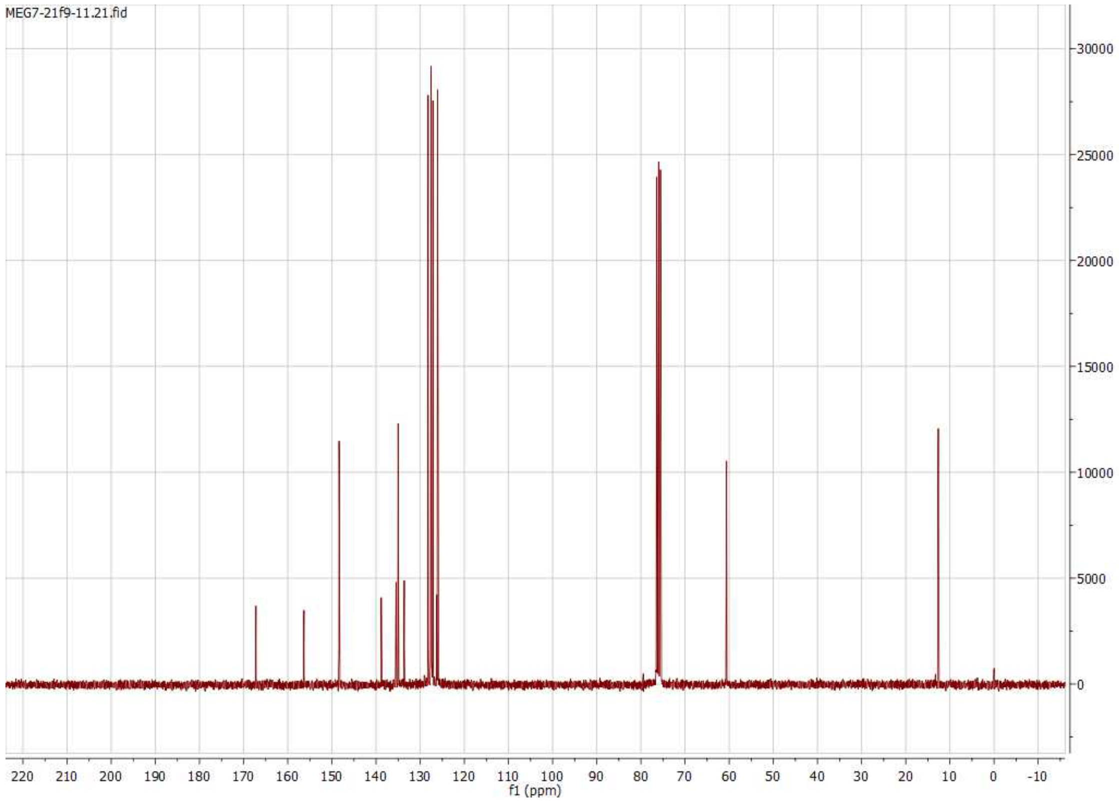

^{1}H NMR of **8d** ($CDCl_3$, 300 MHz)

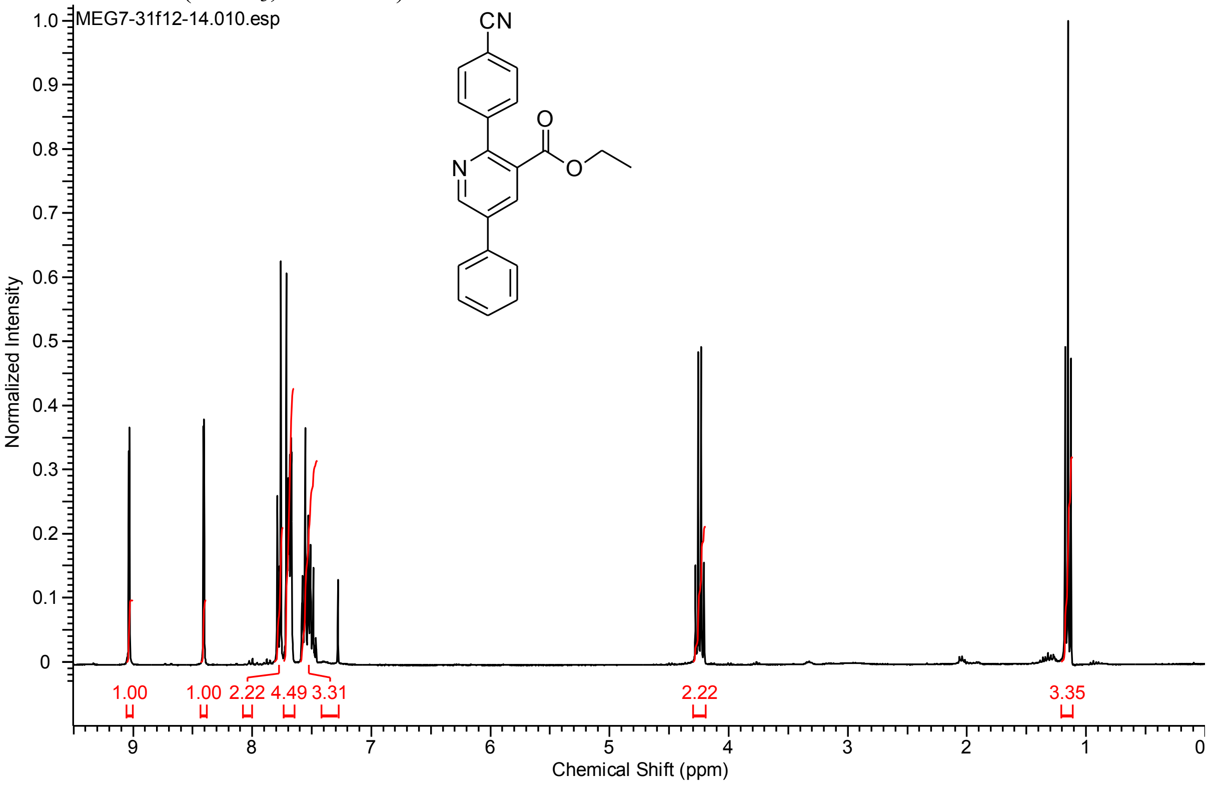

^{13}C NMR of **8d** ($CDCl_3$, 75 MHz)

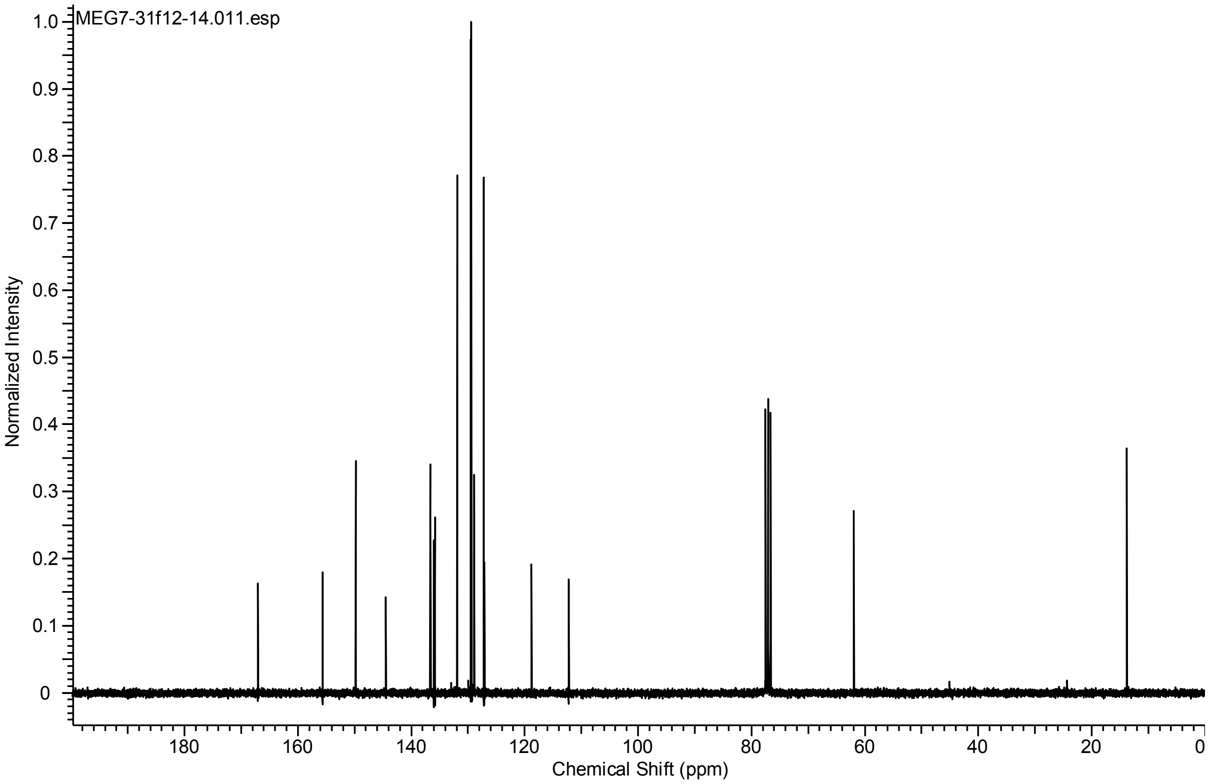

^{1}H NMR of **8e** ($CDCl_3$, 300 MHz)

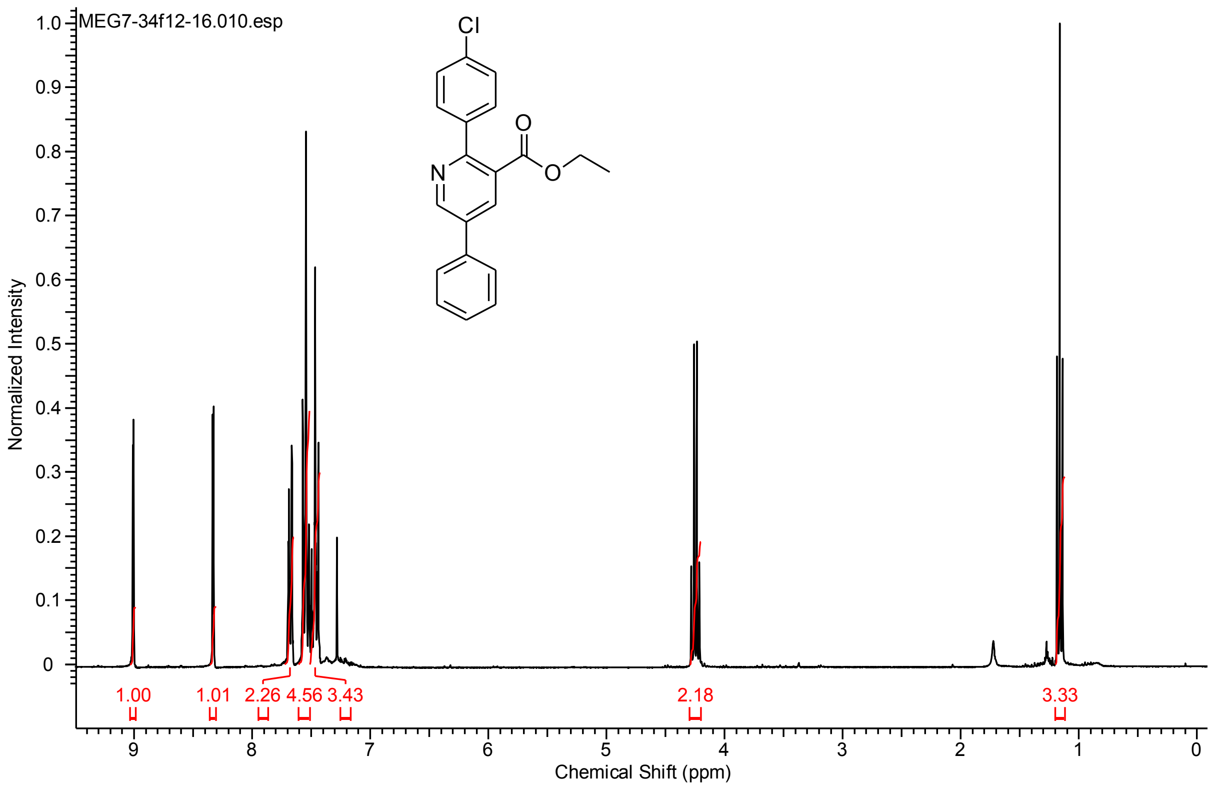

^{13}C NMR of **8e** ($CDCl_3$, 75 MHz)

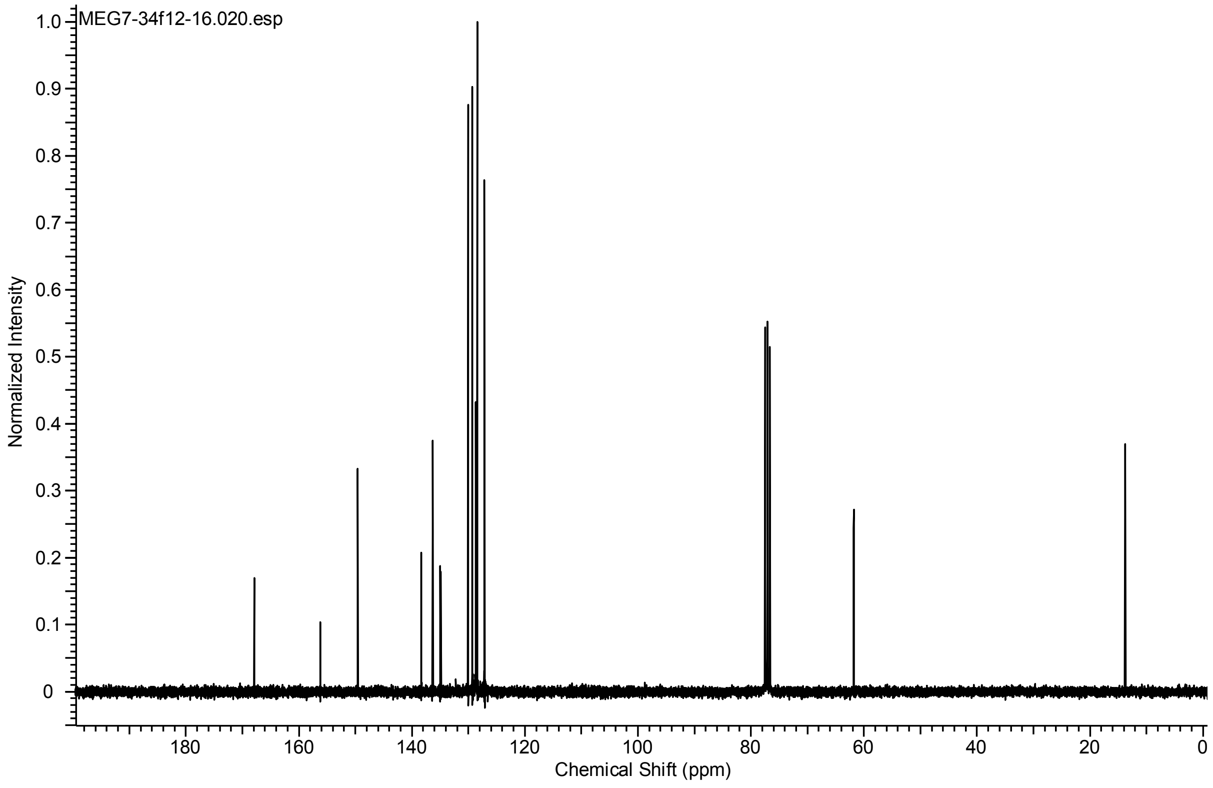

^{1}H NMR of **8f** ($CDCl_3$, 300 MHz)

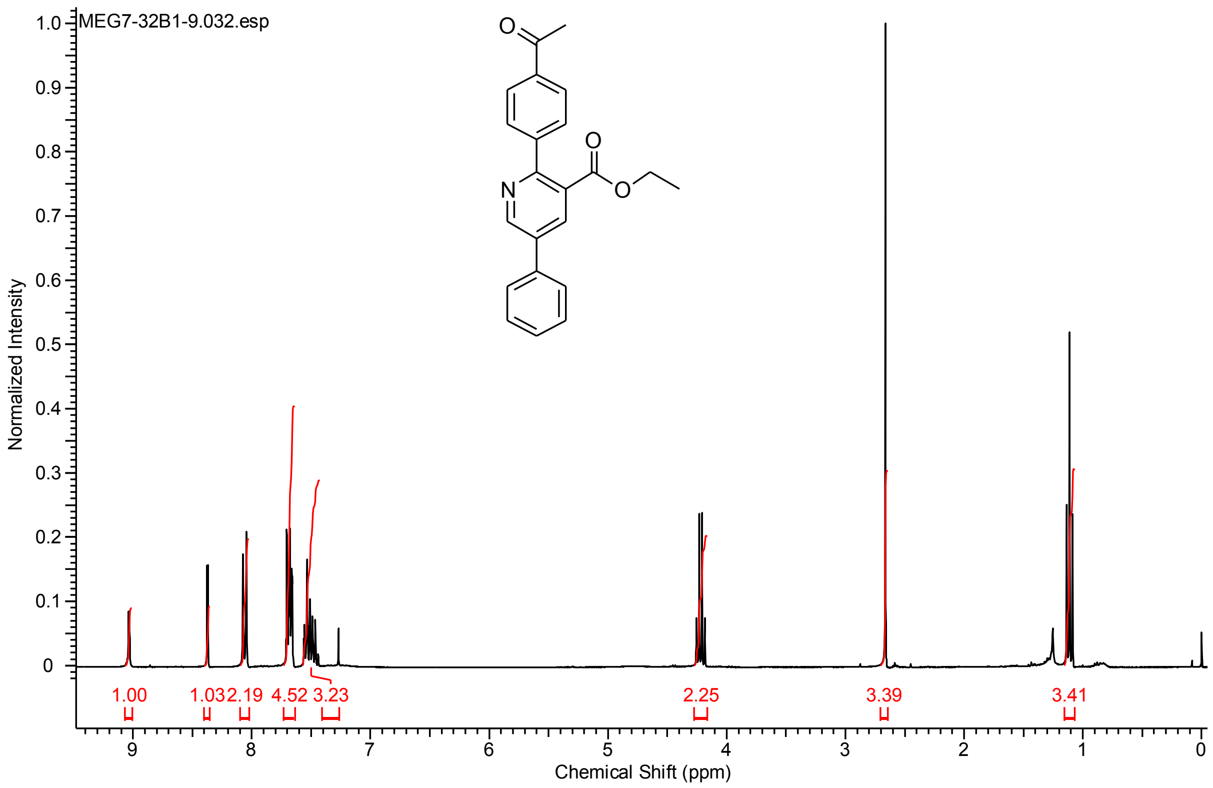

^{13}C NMR of **8f** ($CDCl_3$, 75 MHz)

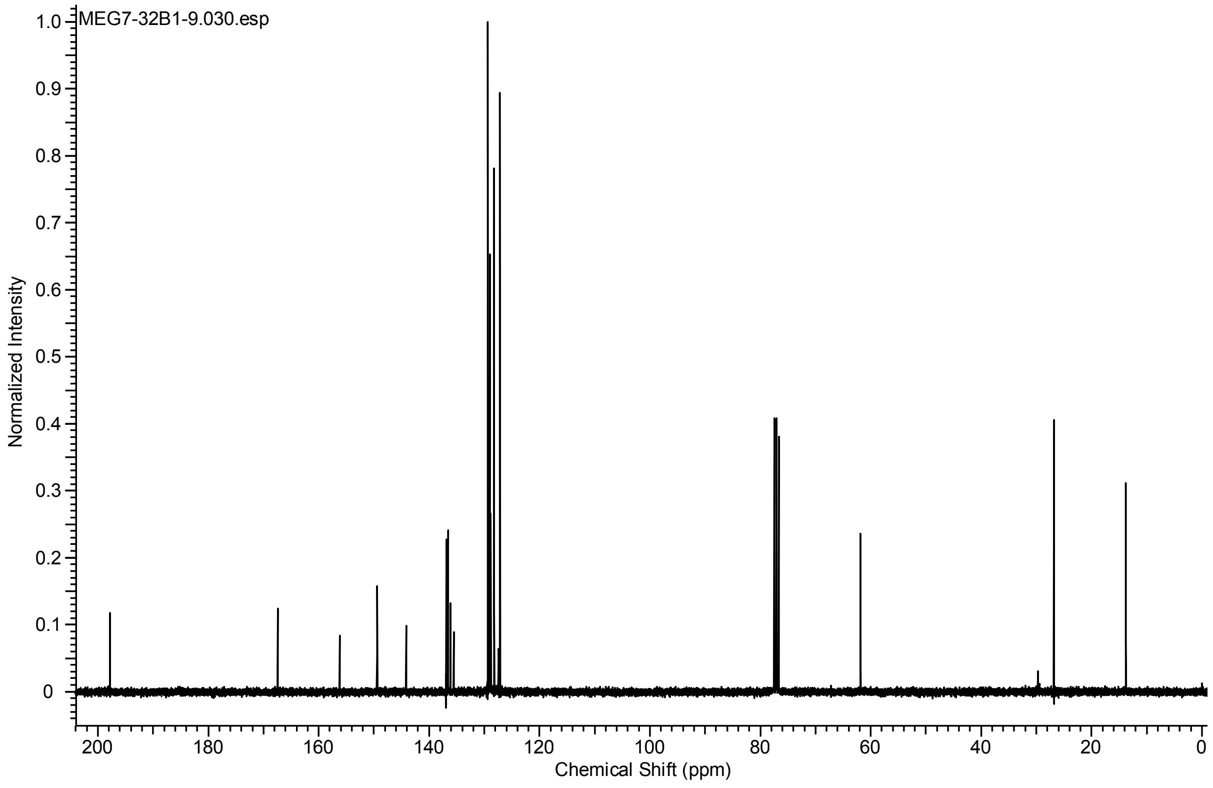

^{1}H NMR of **8g** ($CDCl_3$, 300 MHz)

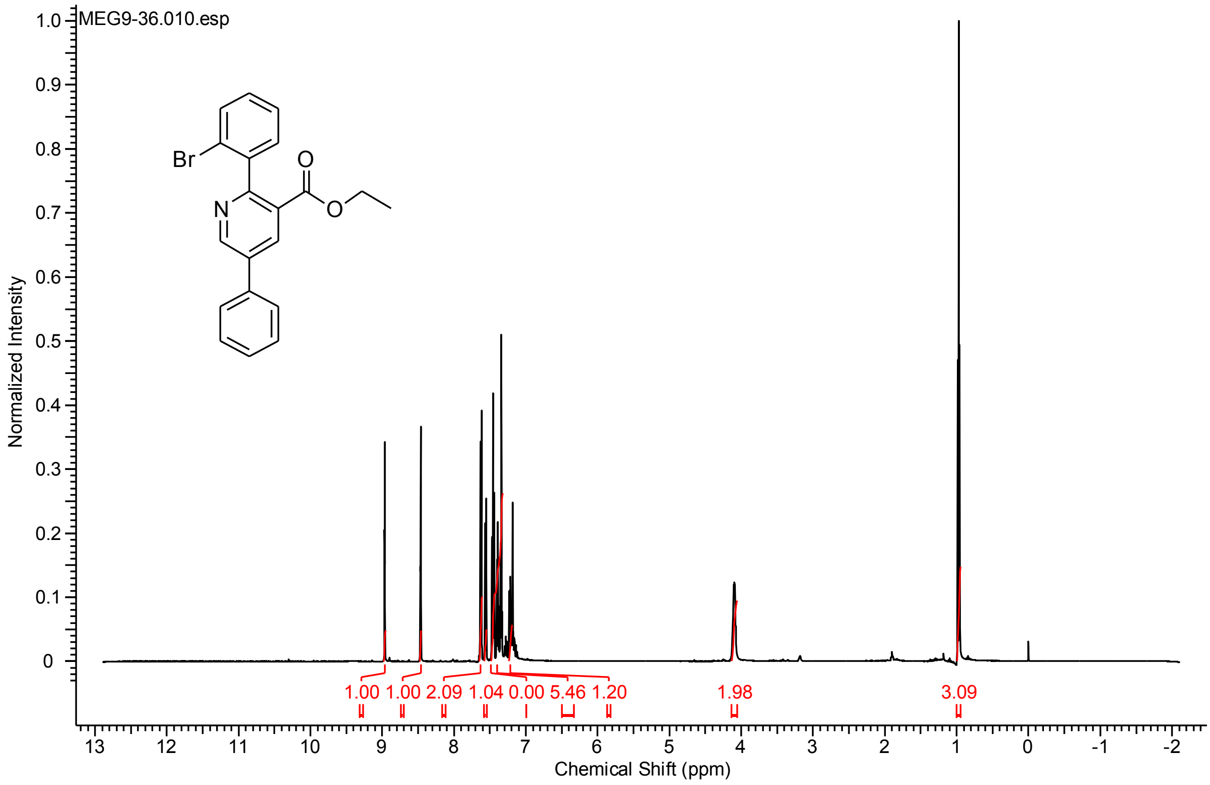

^{13}C NMR of **8g** ($CDCl_3$, 75 MHz)

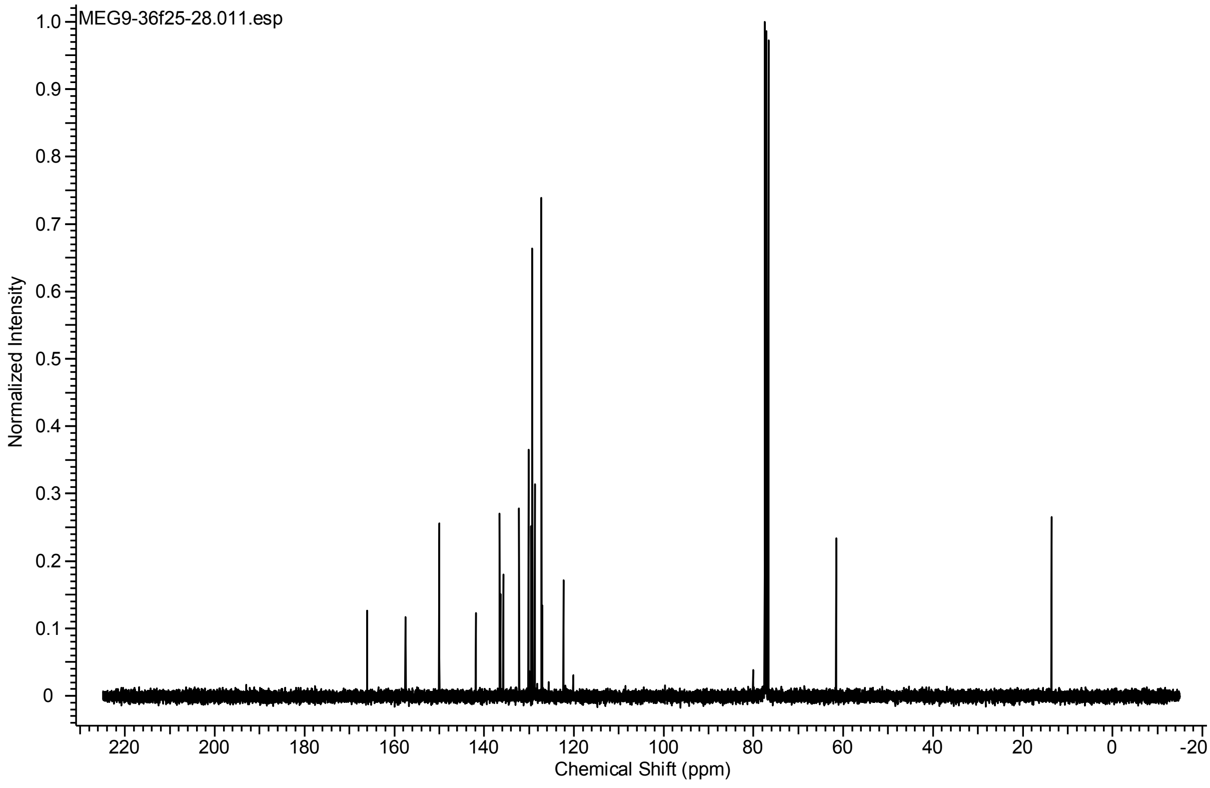

^{1}H NMR of **8h** ($CDCl_3$, 300 MHz)

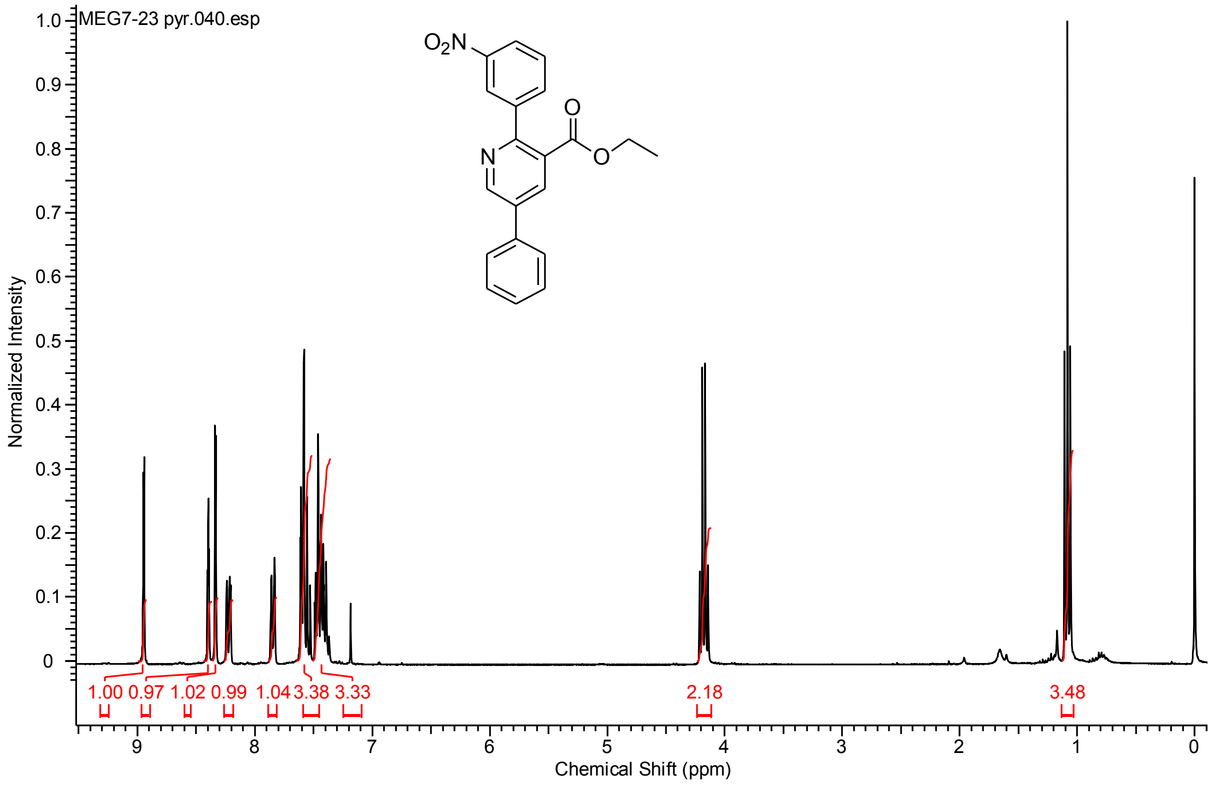

^{13}C NMR of **8h** ($CDCl_3$, 75 MHz)

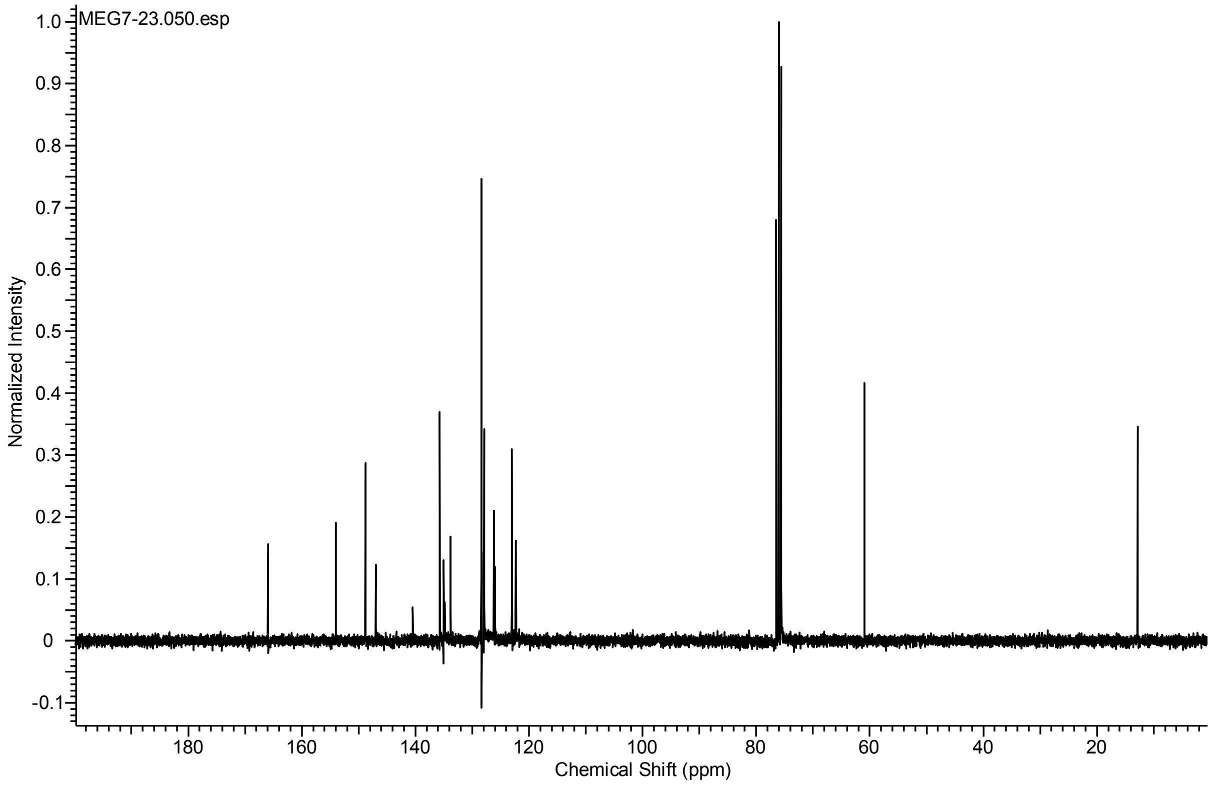

^{1}H NMR of **8i** ($CDCl_3$, 300 MHz)

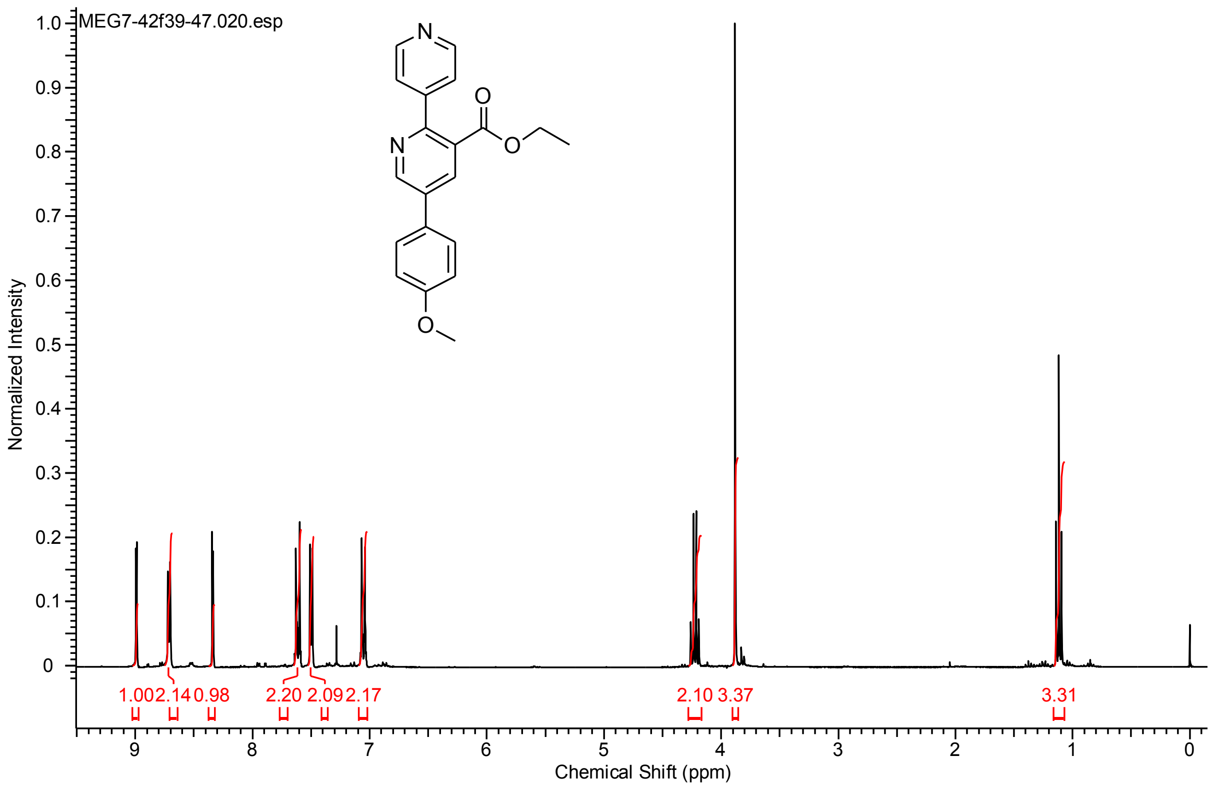

^{13}C NMR of **8i** ($CDCl_3$, 75 MHz)

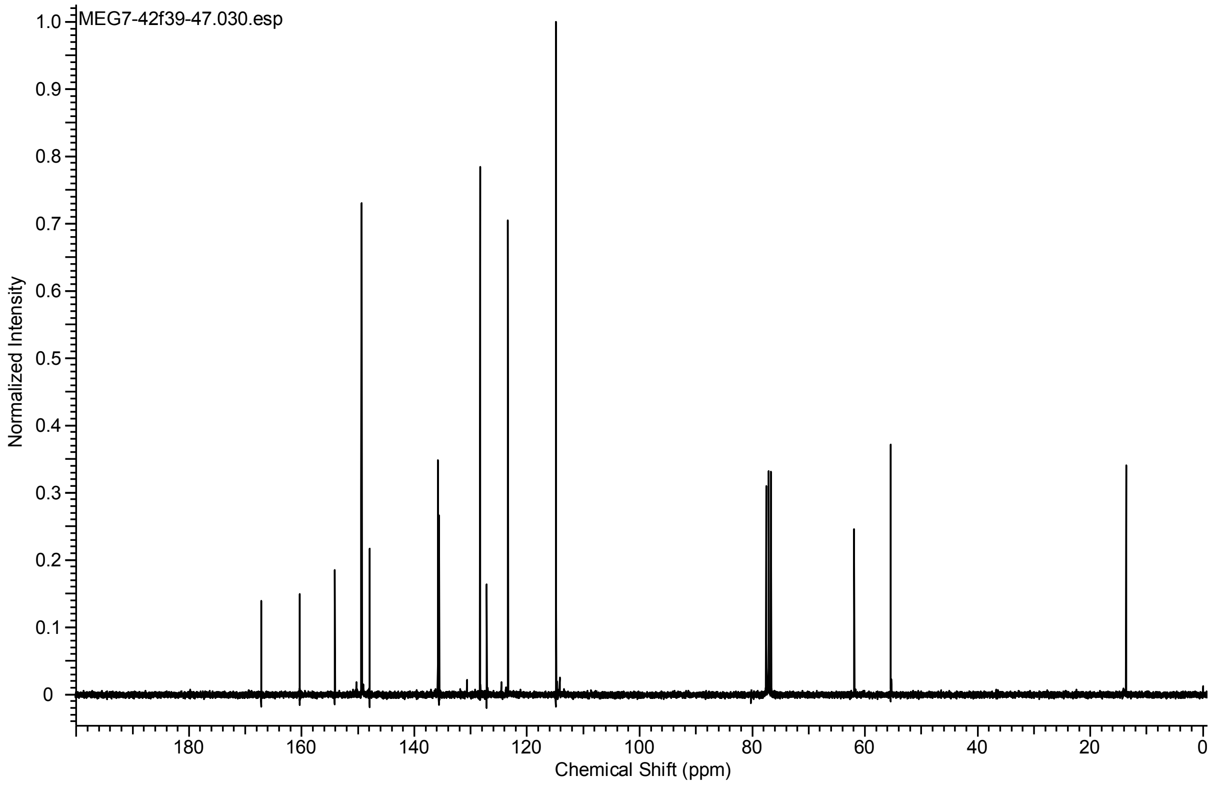

^{1}H NMR of **8j** ($CDCl_3$, 300 MHz)

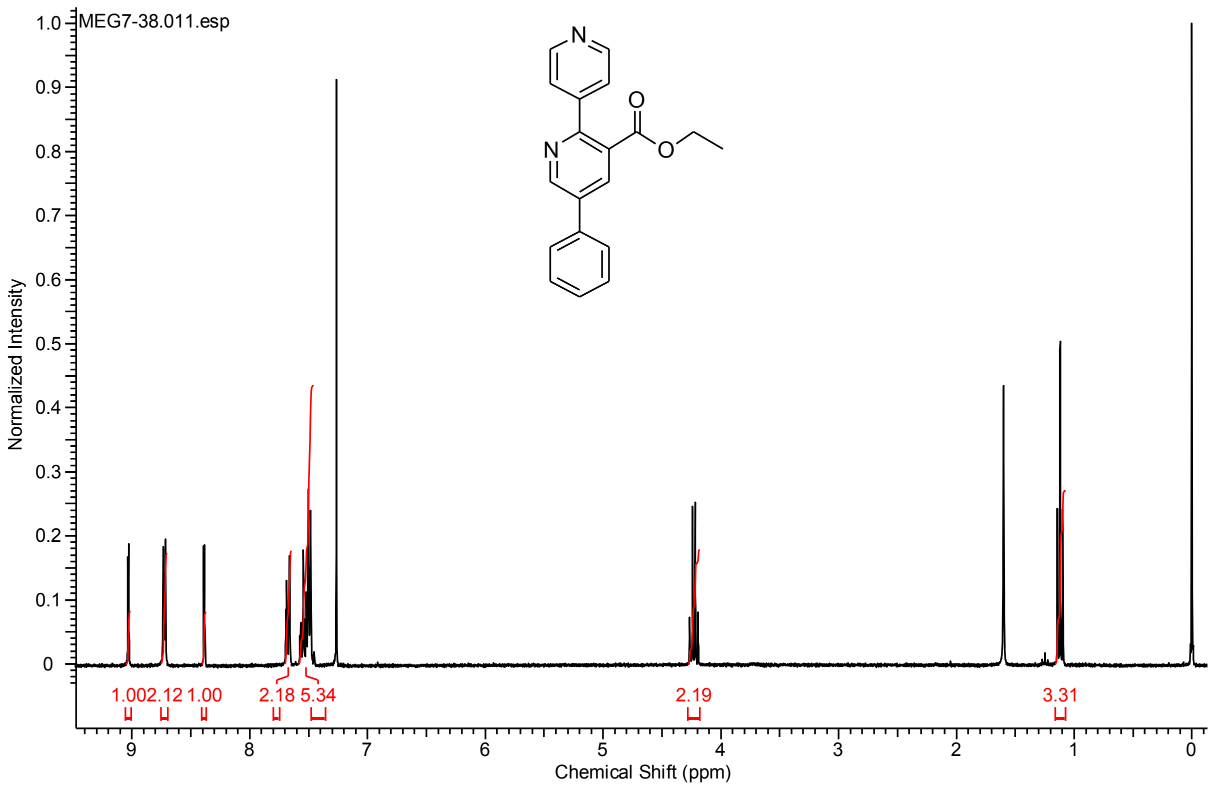

^{13}C NMR of **8j** ($CDCl_3$, 75 MHz)

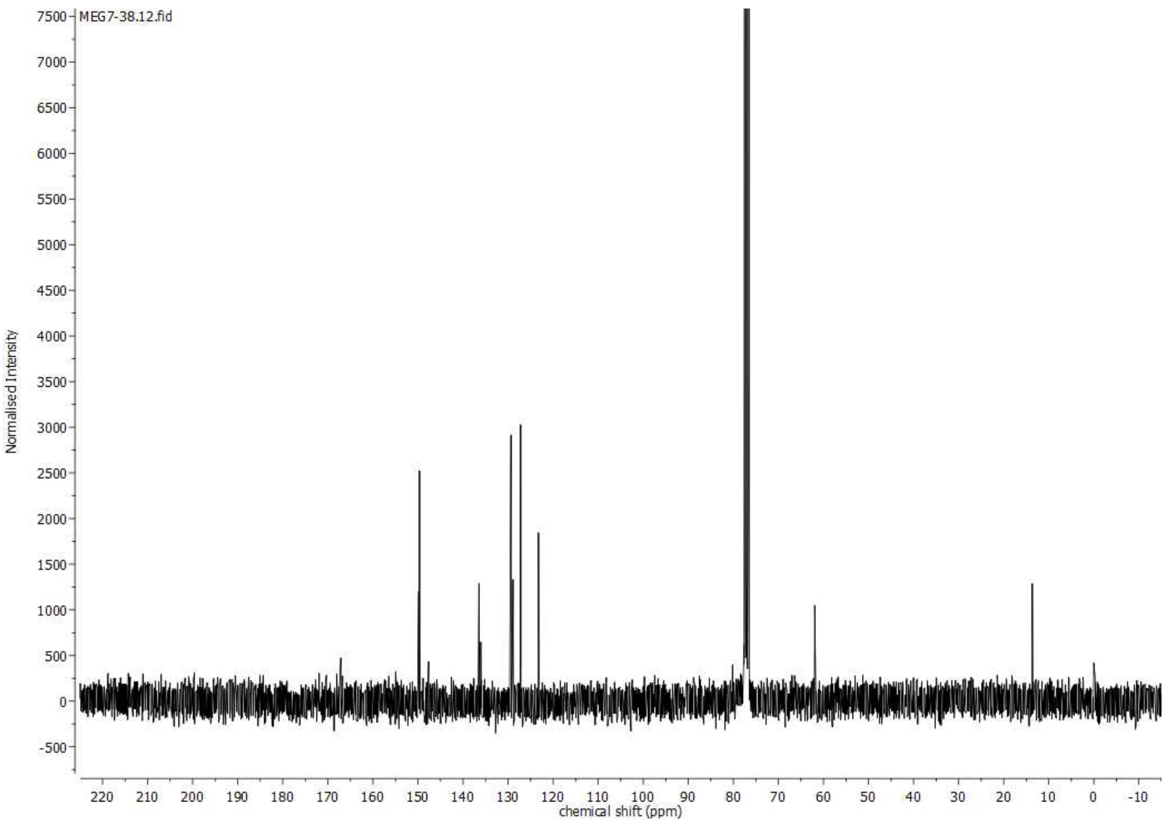

^{1}H NMR of **8k** ($CDCl_3$, 300 MHz)

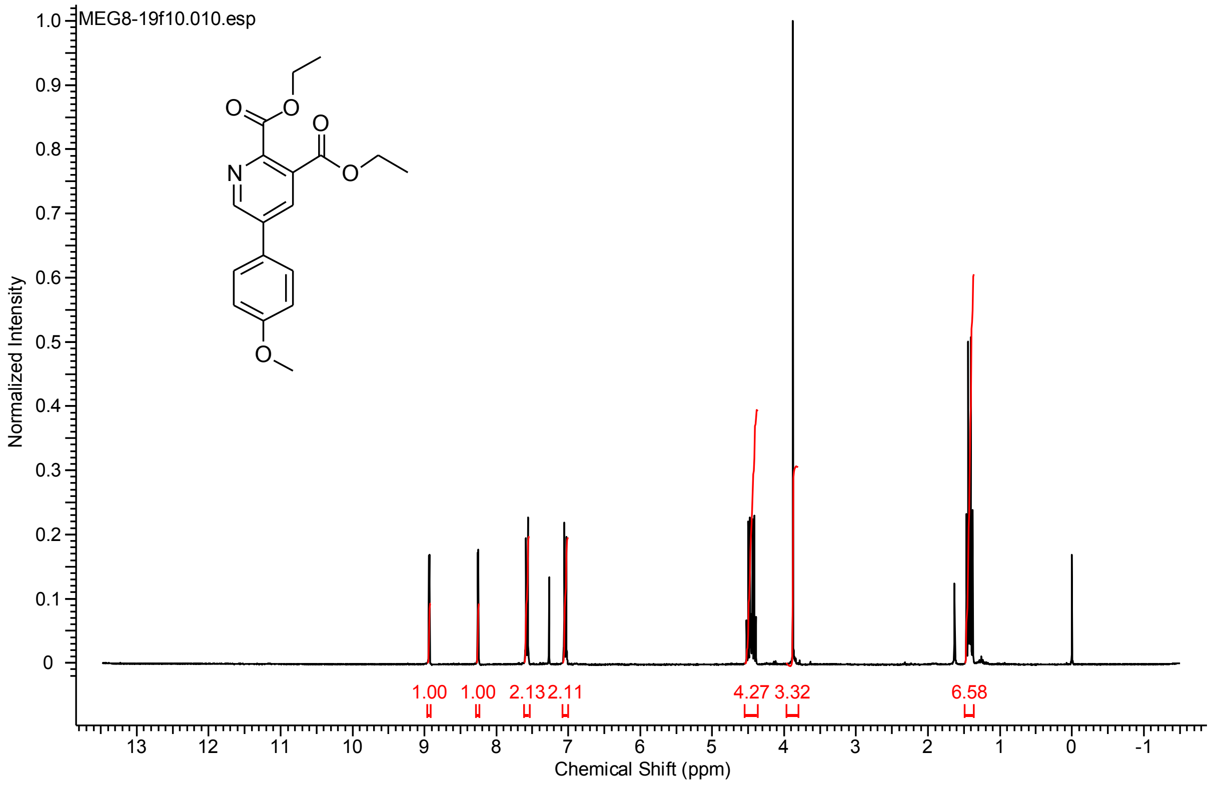

^{13}C NMR of **8k** ($CDCl_3$, 75 MHz)

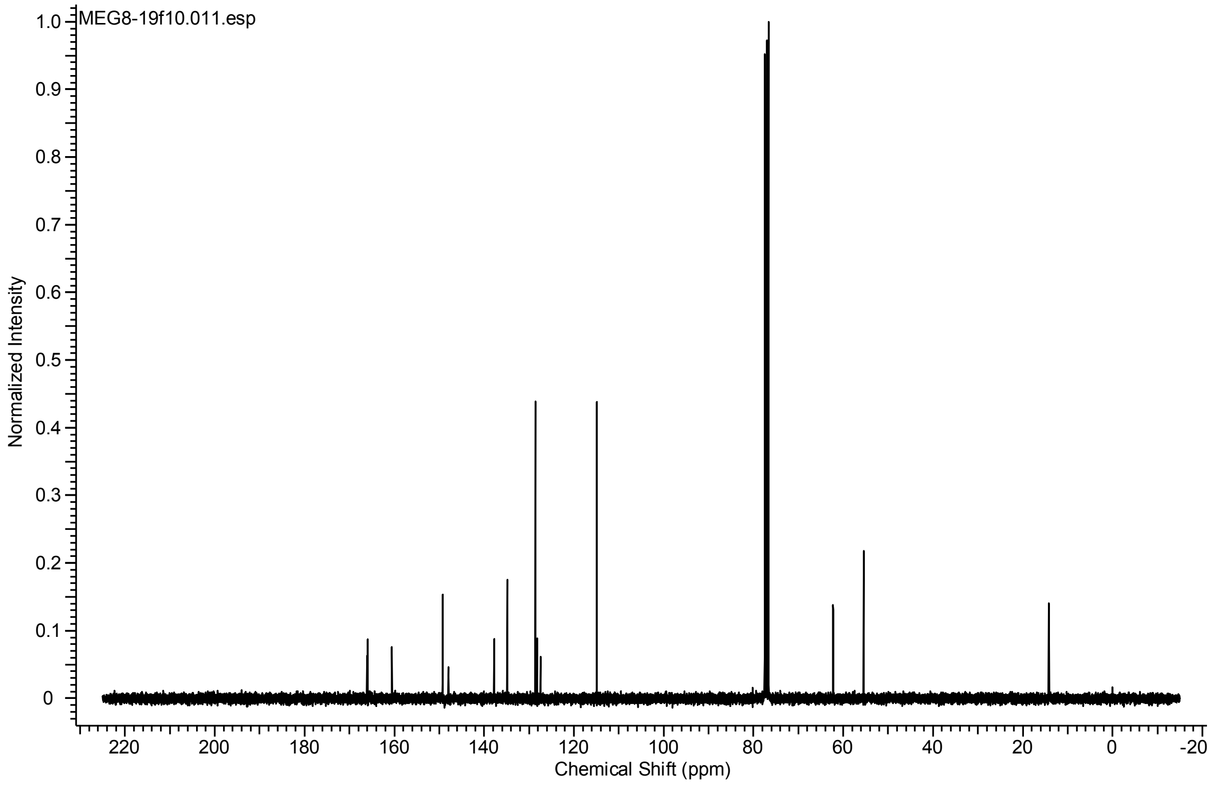

^{1}H NMR of **8l** ($CDCl_3$, 300 MHz)

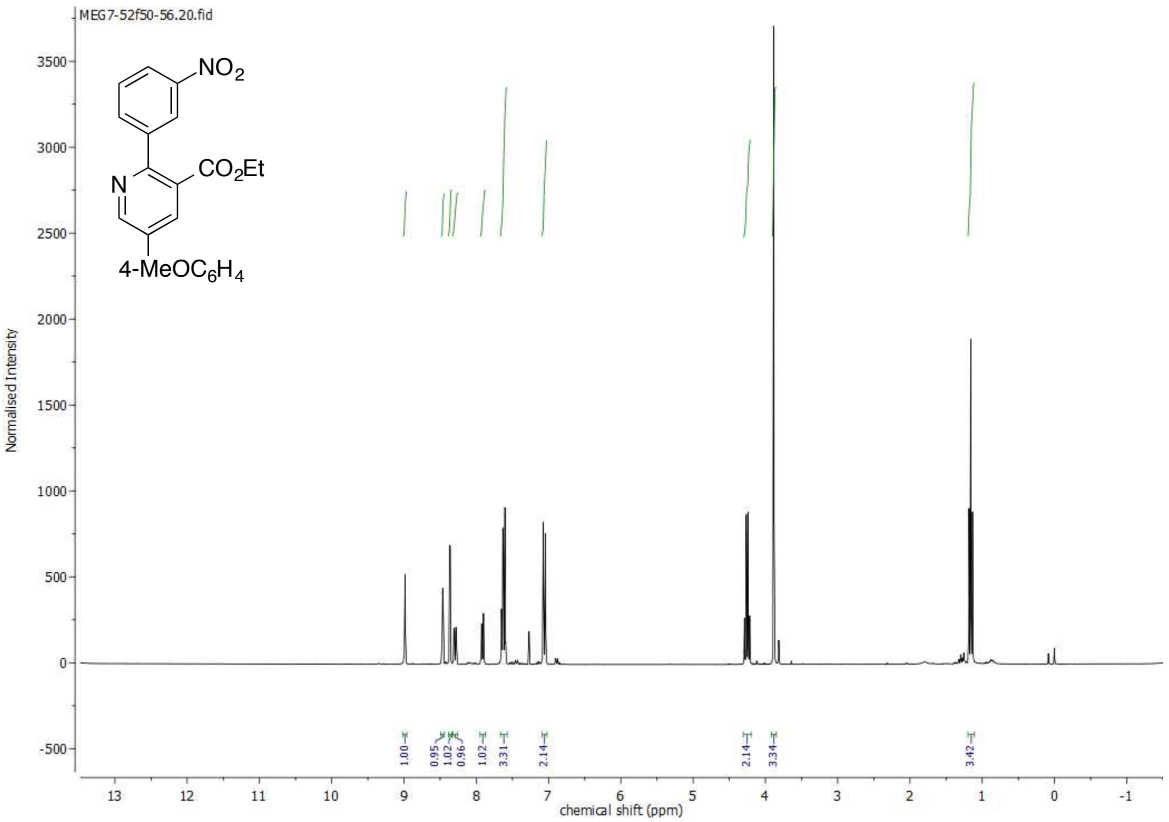

^{13}C NMR of **8l** ($CDCl_3$, 75 MHz)

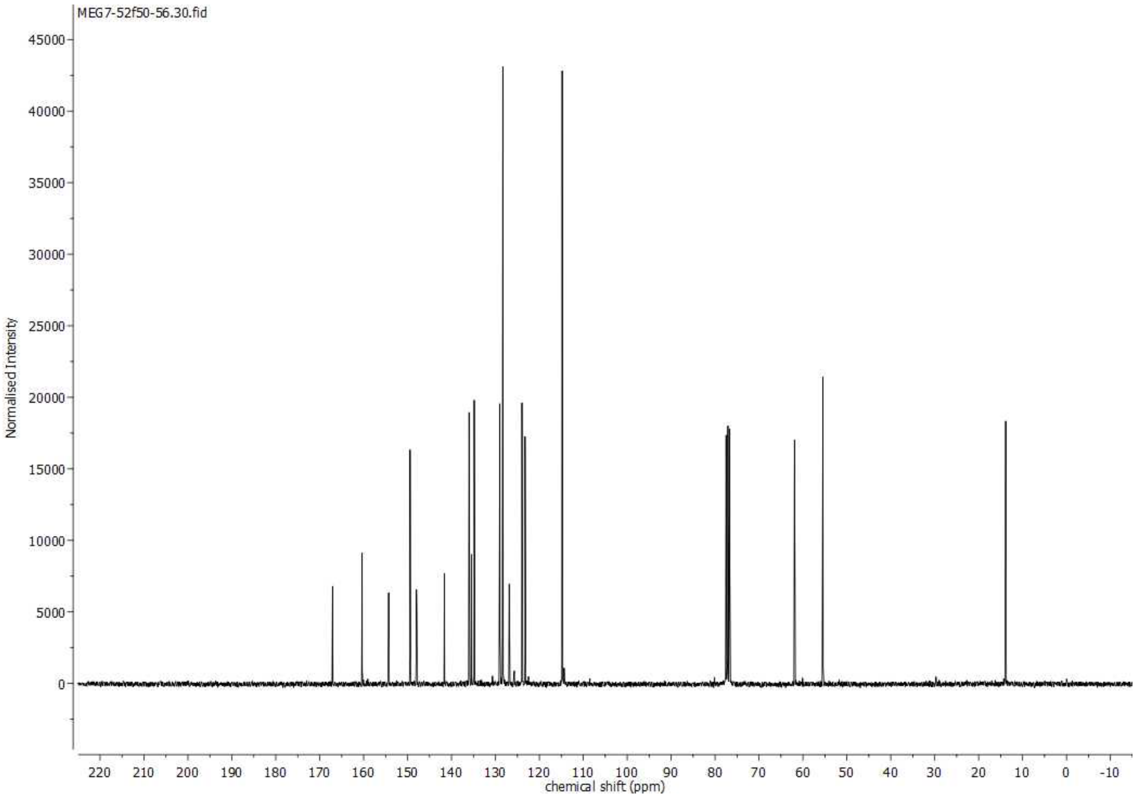

^{1}H NMR of **8m** ($CDCl_3$, 300 MHz)

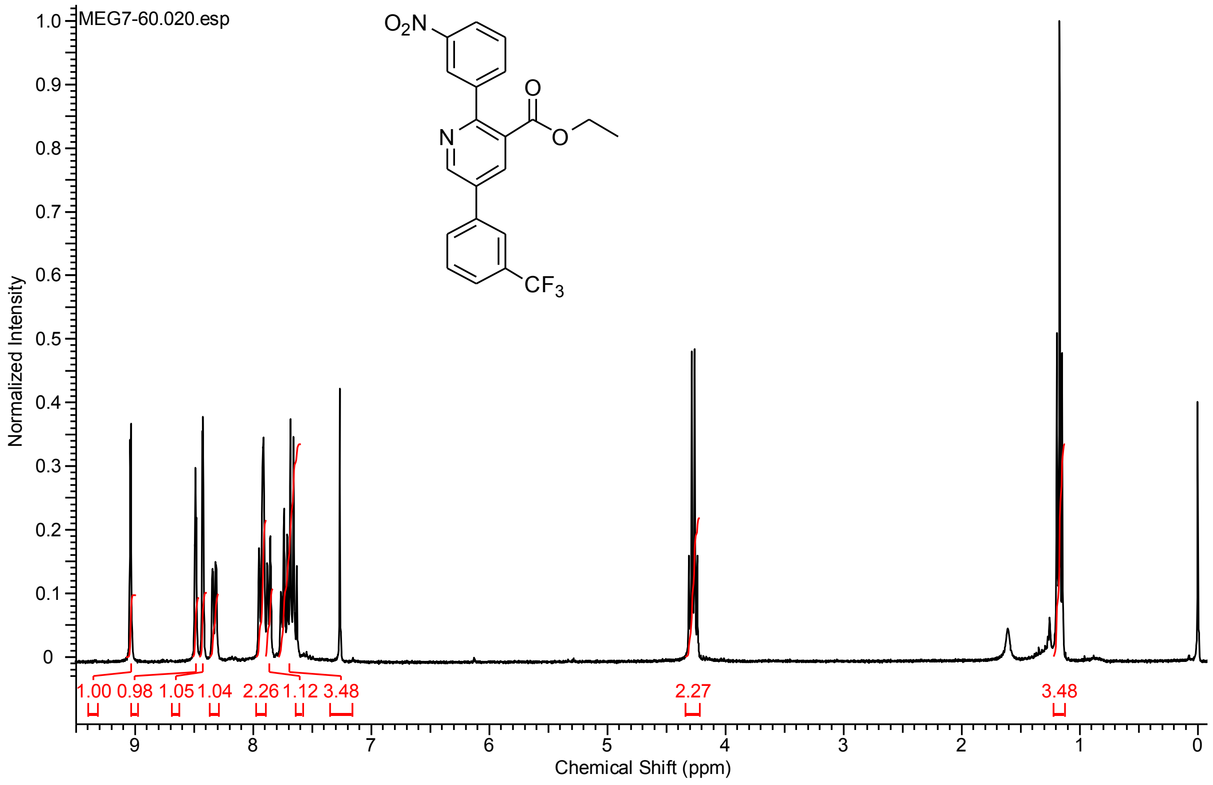

^{13}C NMR of **8m** ($CDCl_3$, 75 MHz)

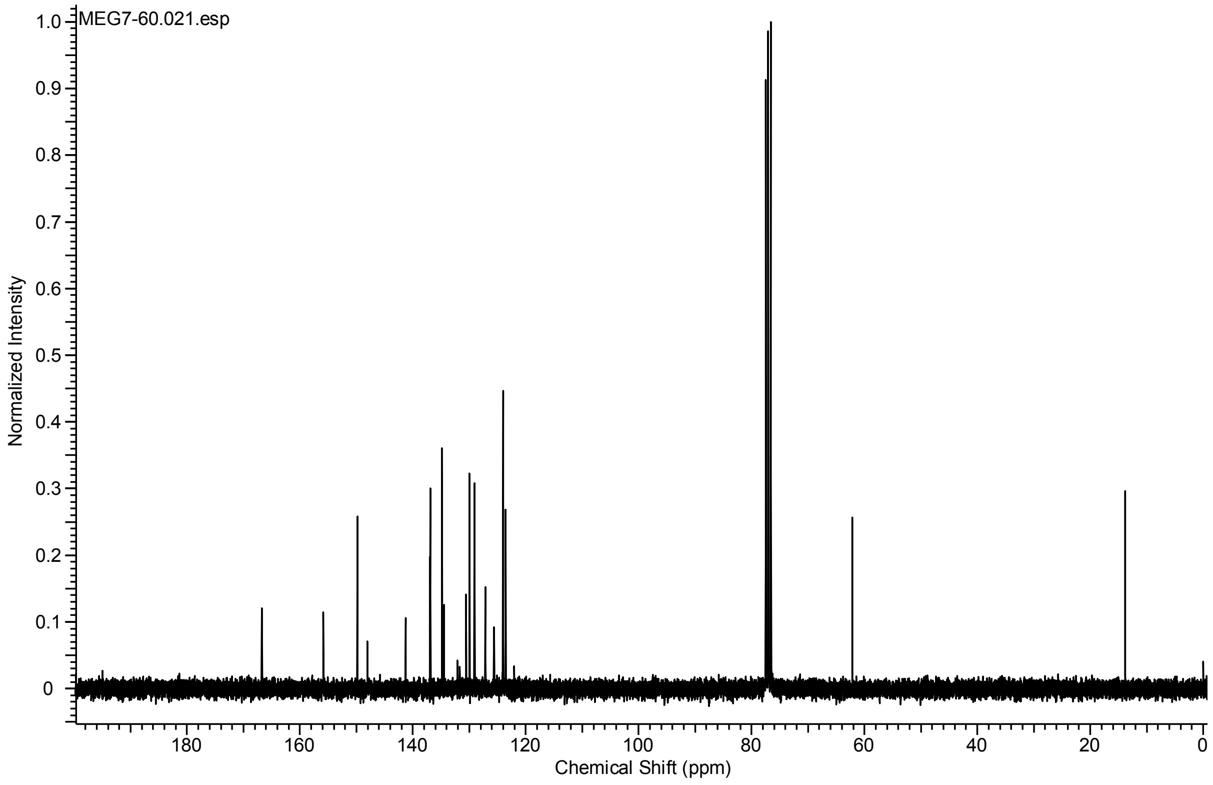

^{1}H NMR of **8n** ($CDCl_3$, 300 MHz)

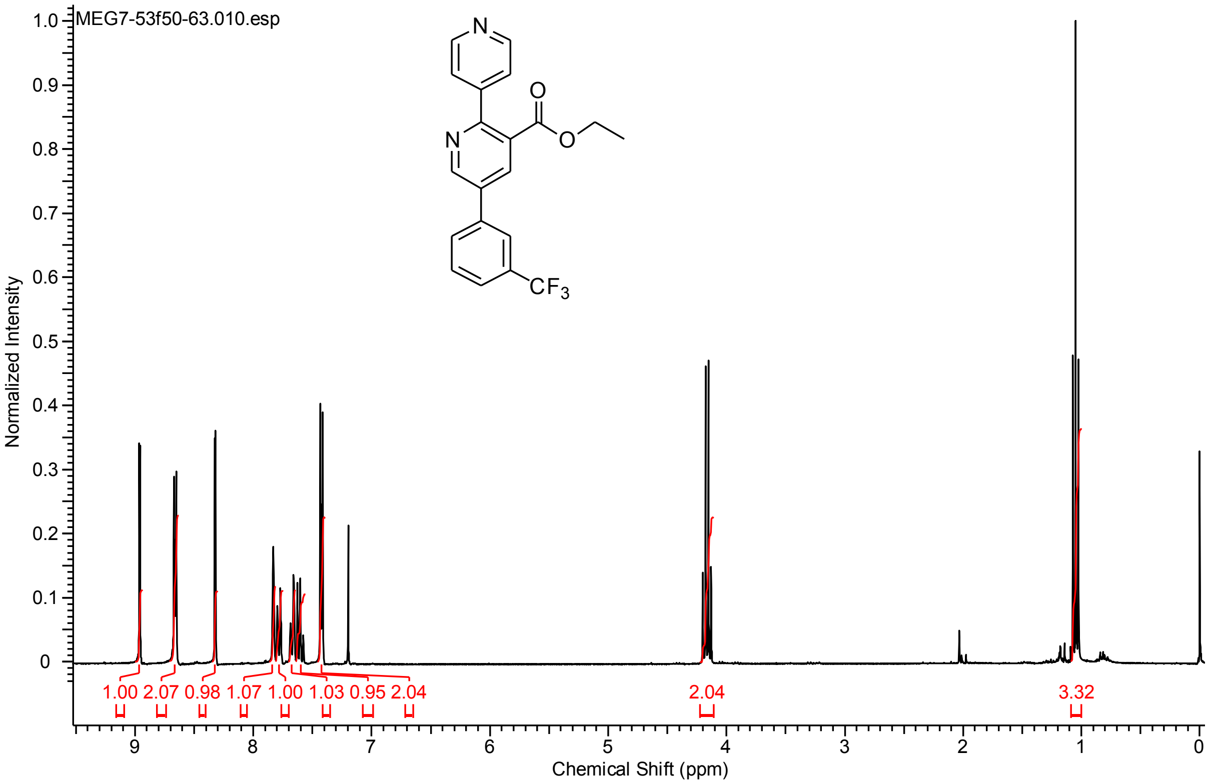

^{13}C NMR of **8n** ($CDCl_3$, 75 MHz)

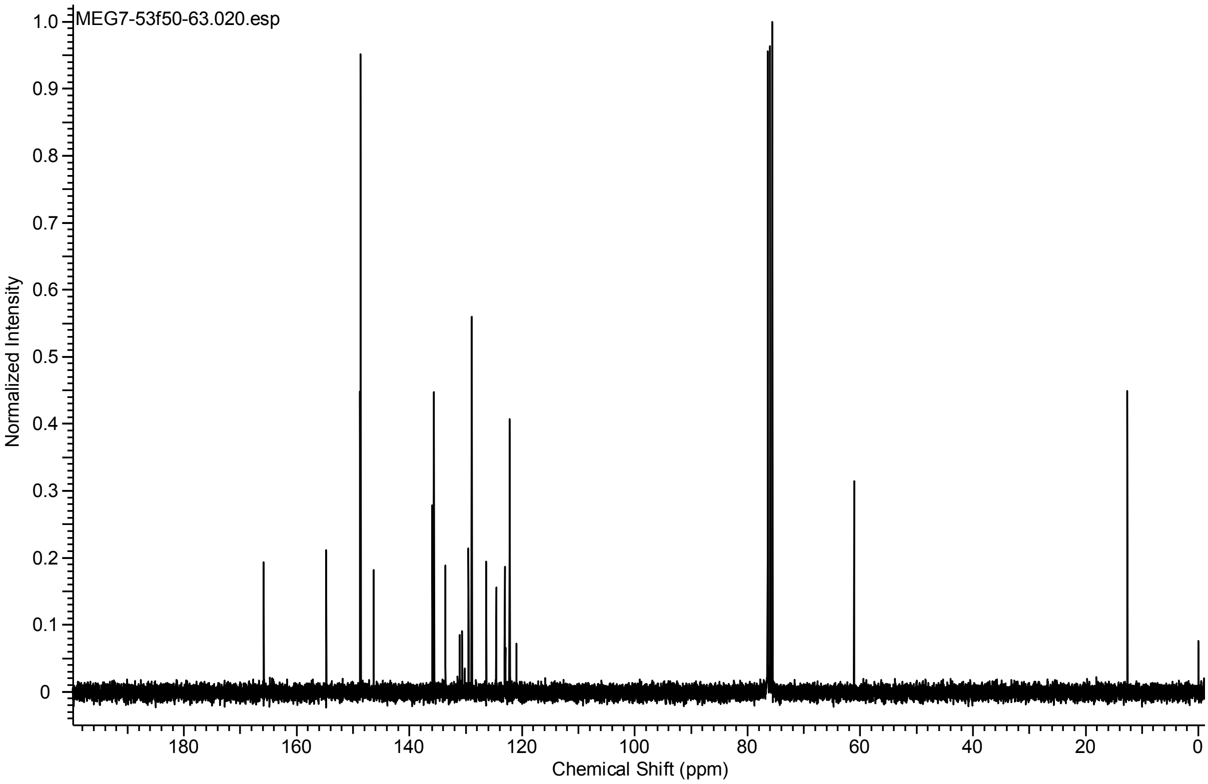

^{1}H NMR of **8o** ($CDCl_3$, 300 MHz)

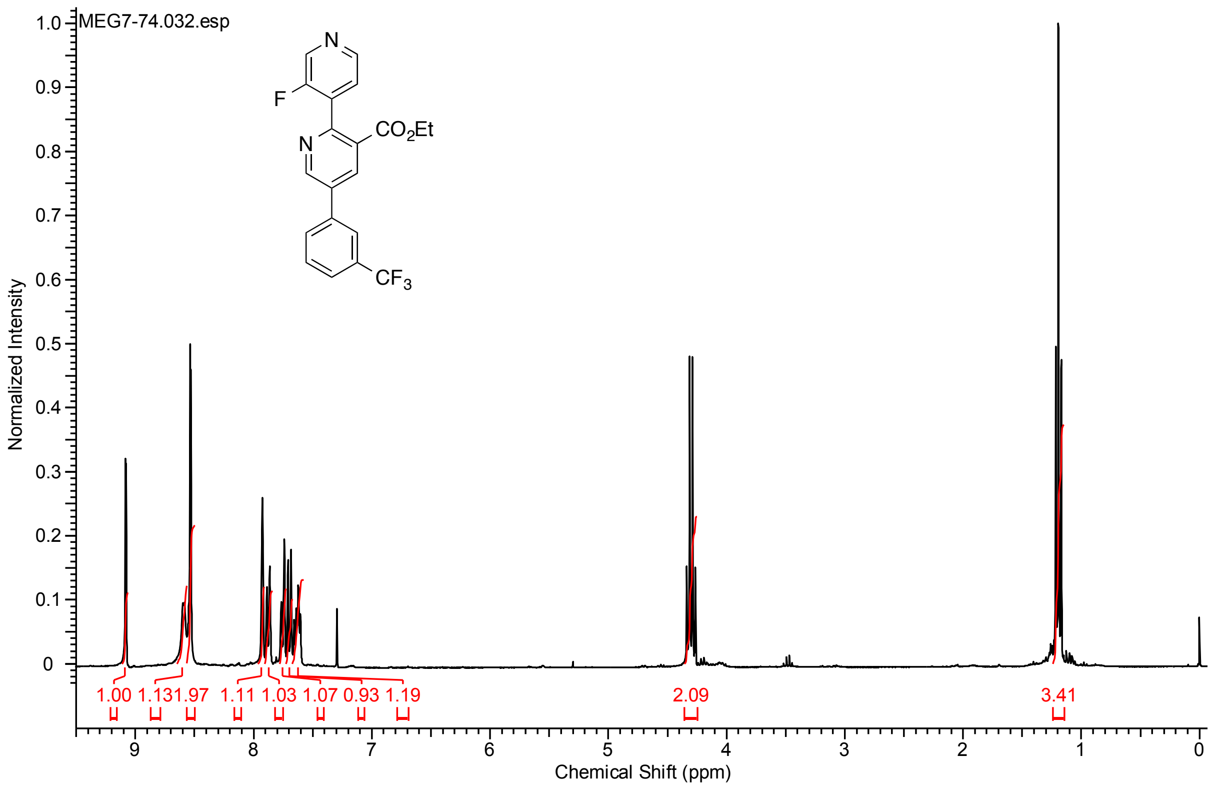

^{13}C NMR of **8o** ($CDCl_3$, 75 MHz)

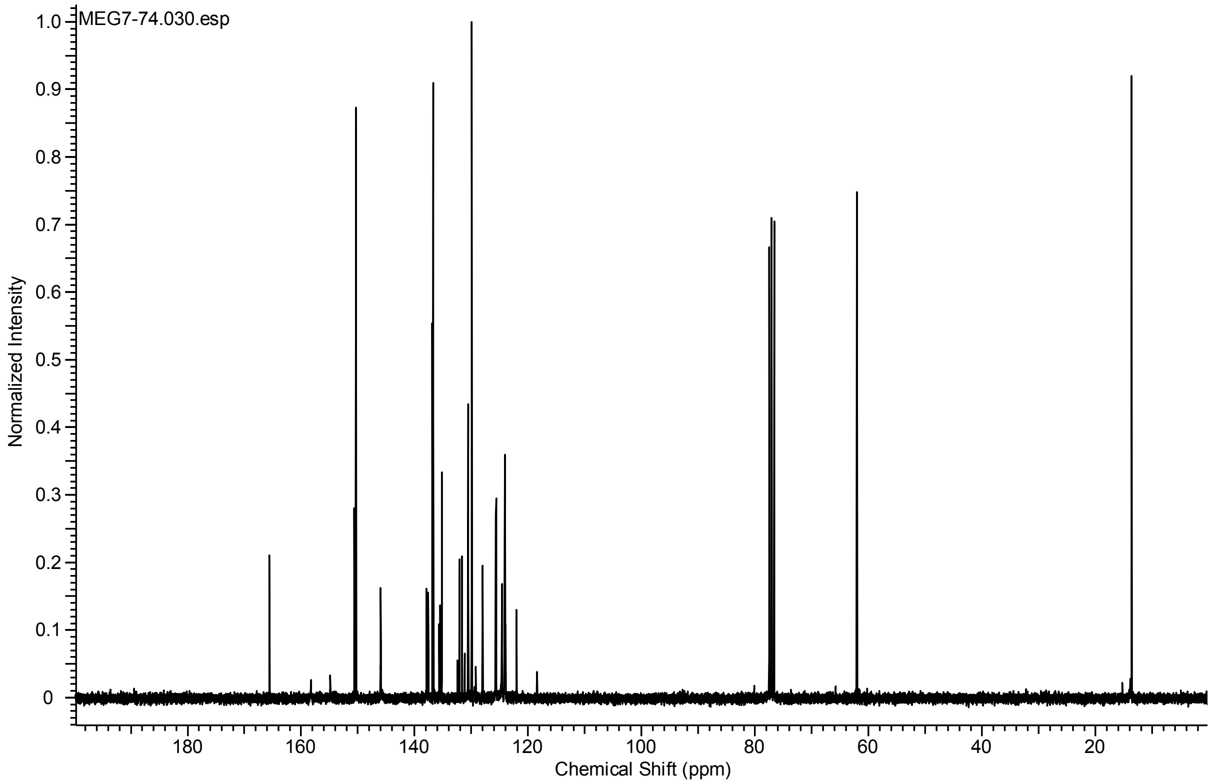

^{1}H NMR of **8p** ($CDCl_3$, 300 MHz)

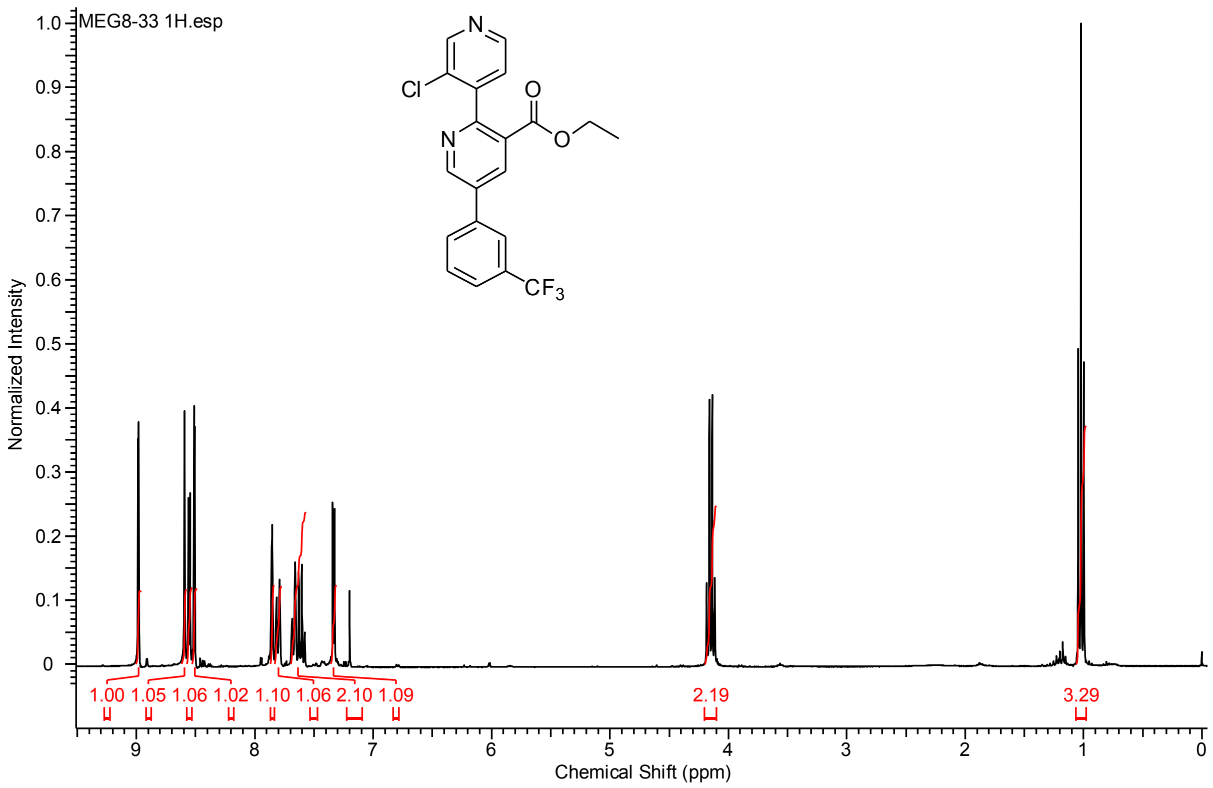

^{13}C NMR of **8p** ($CDCl_3$, 75 MHz)

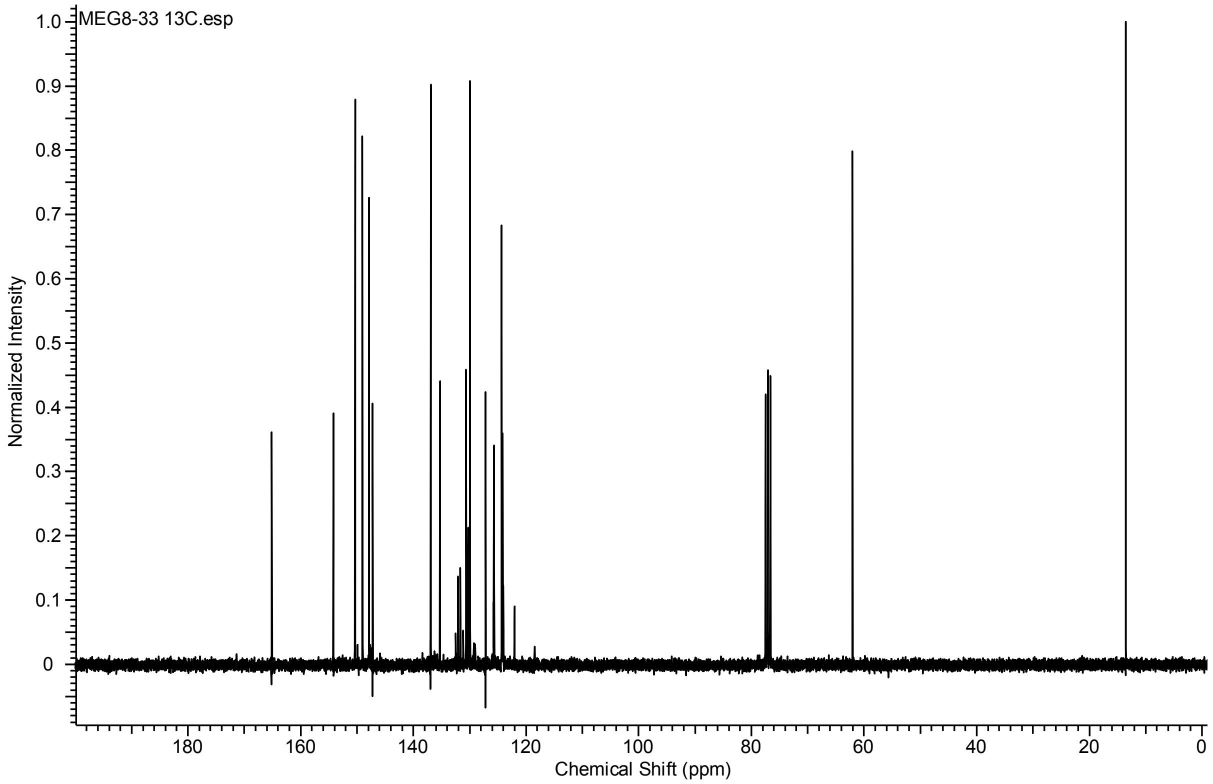

^{1}H NMR of **8q** ($CDCl_3$, 300 MHz)

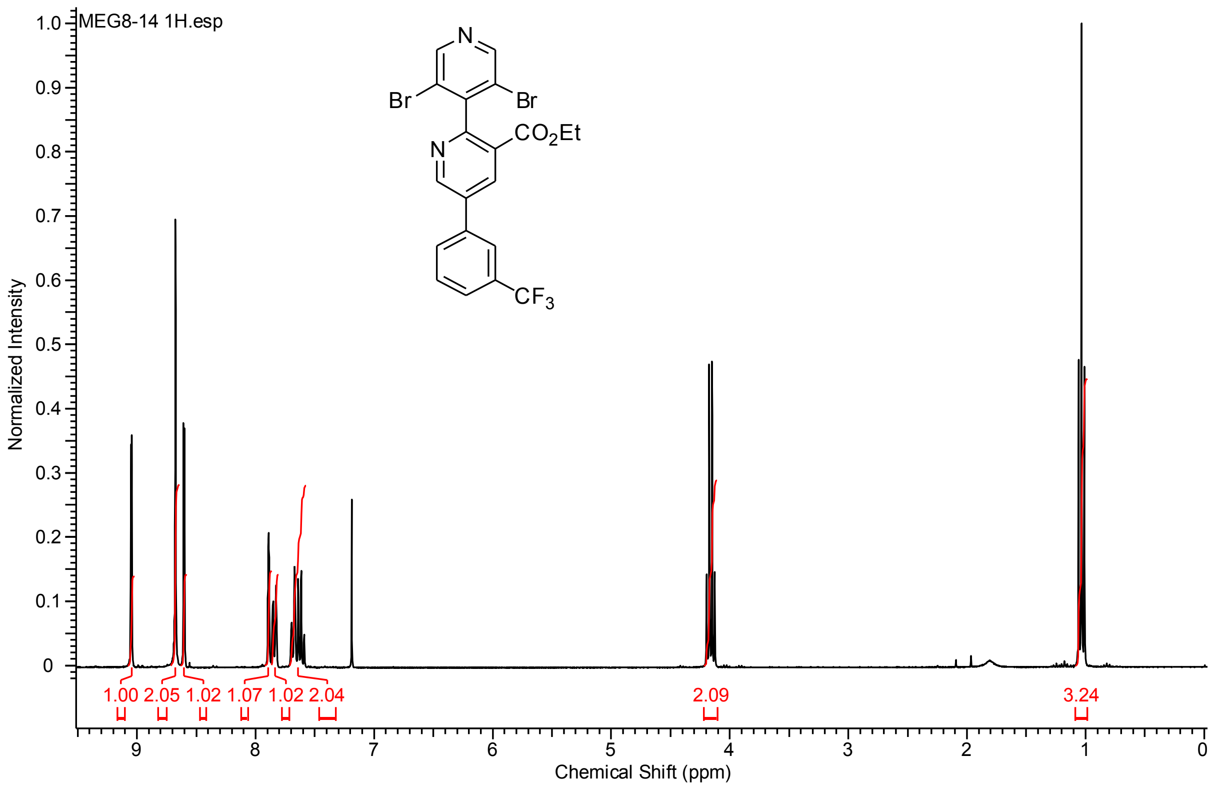

^{13}C NMR of **8q** ($CDCl_3$, 75 MHz)

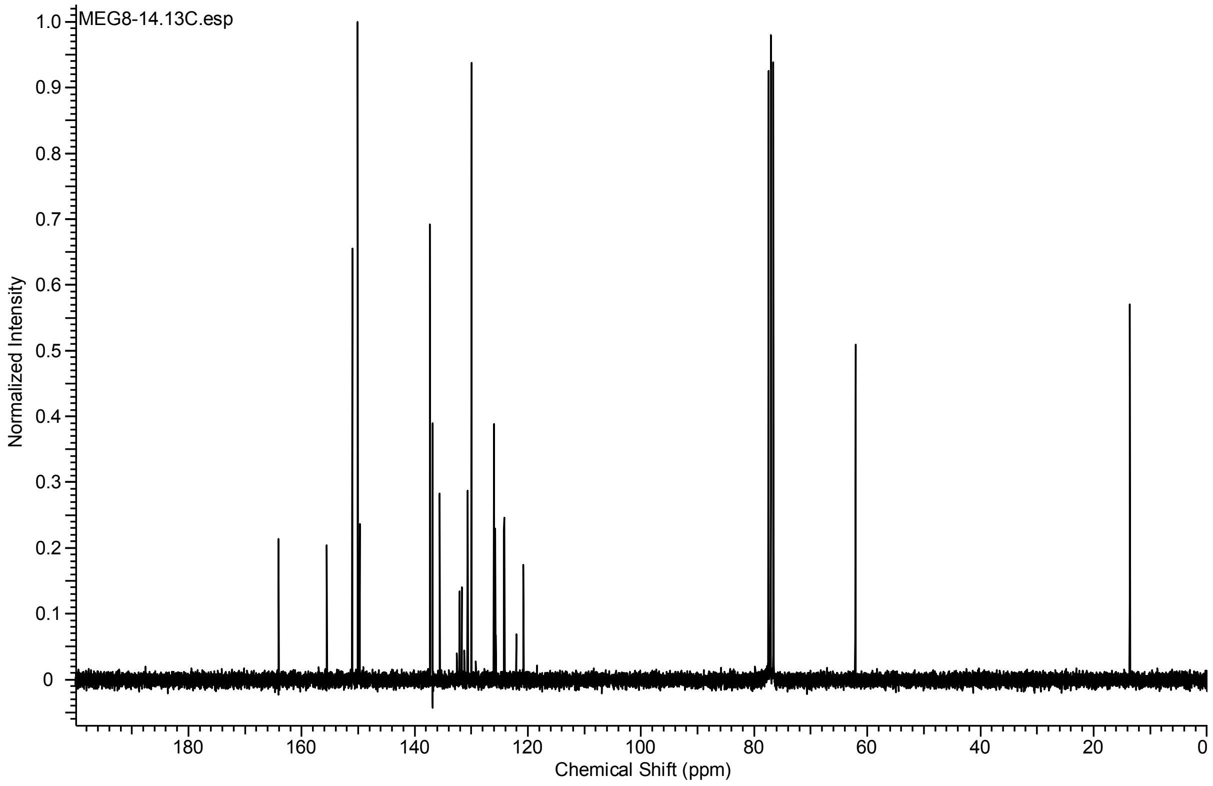

^{1}H NMR of **8r** ($CDCl_3$, 300 MHz)

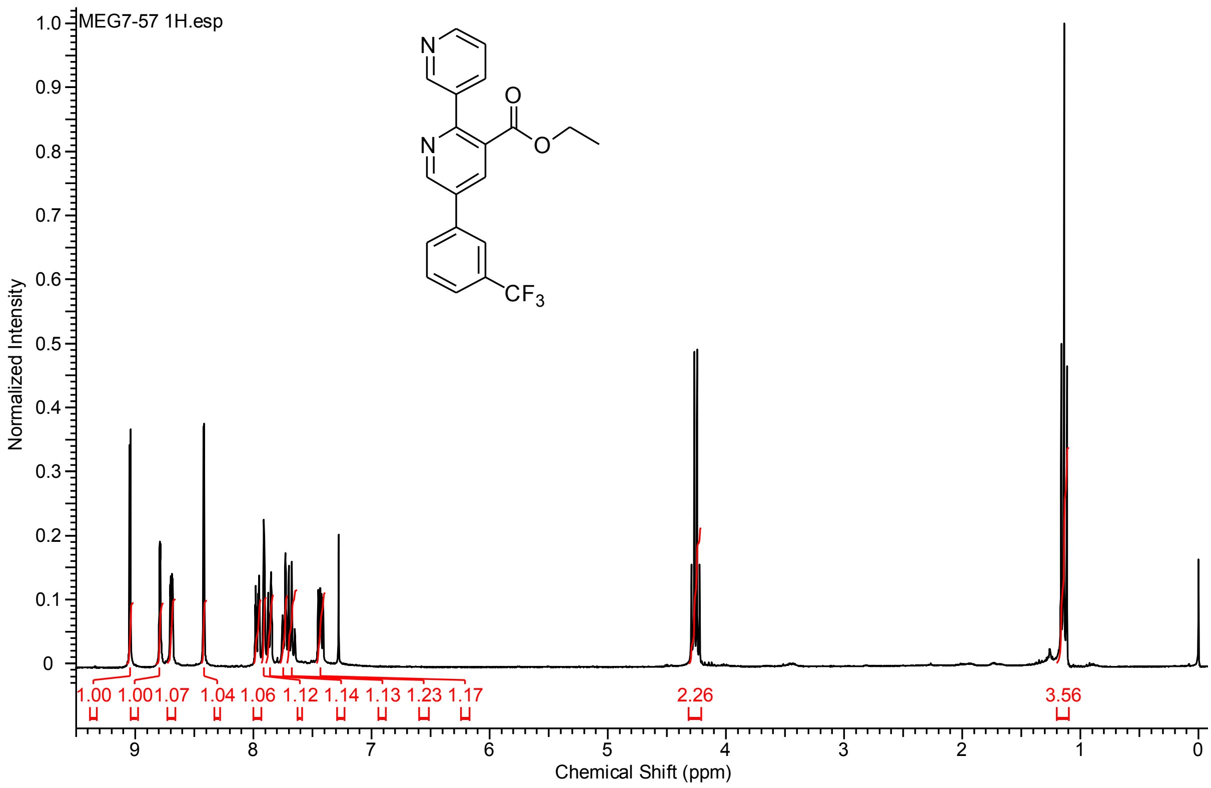

^{13}C NMR of **8r** ($CDCl_3$, 75 MHz)

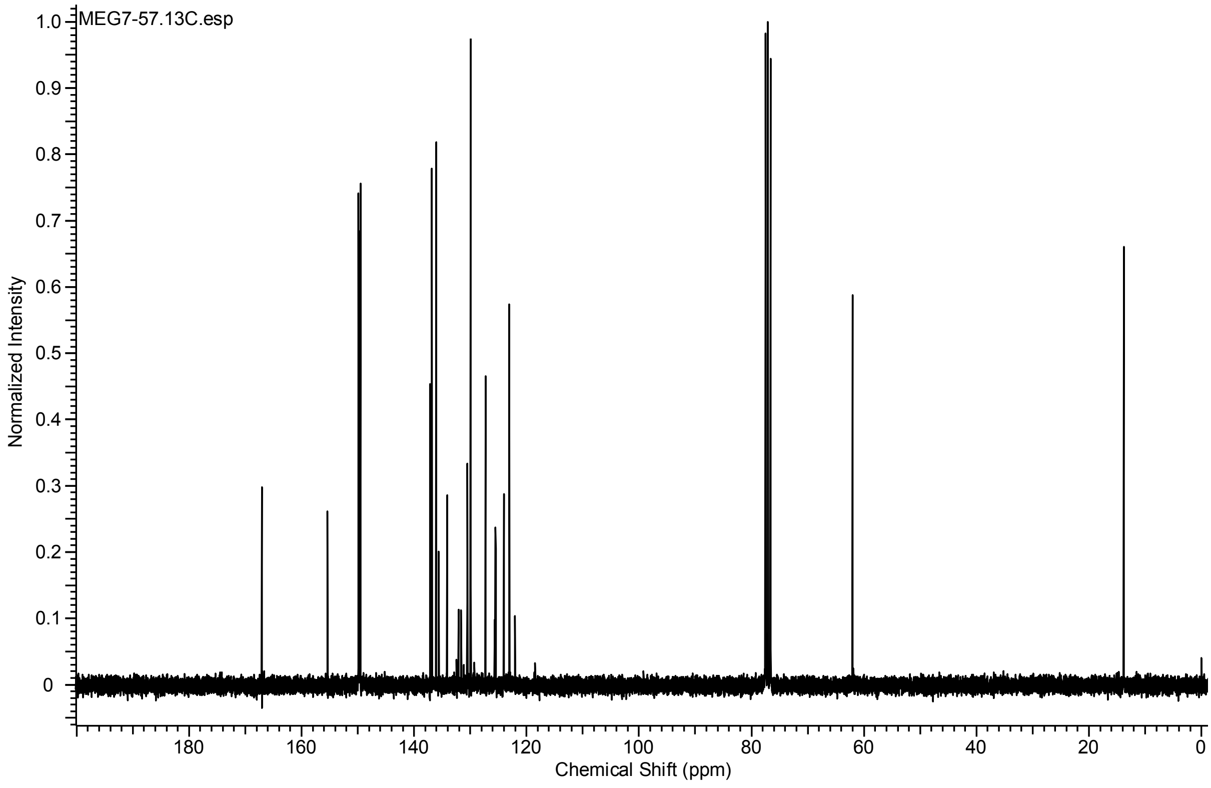

^{1}H NMR of **8t** ($CDCl_3$, 300 MHz)

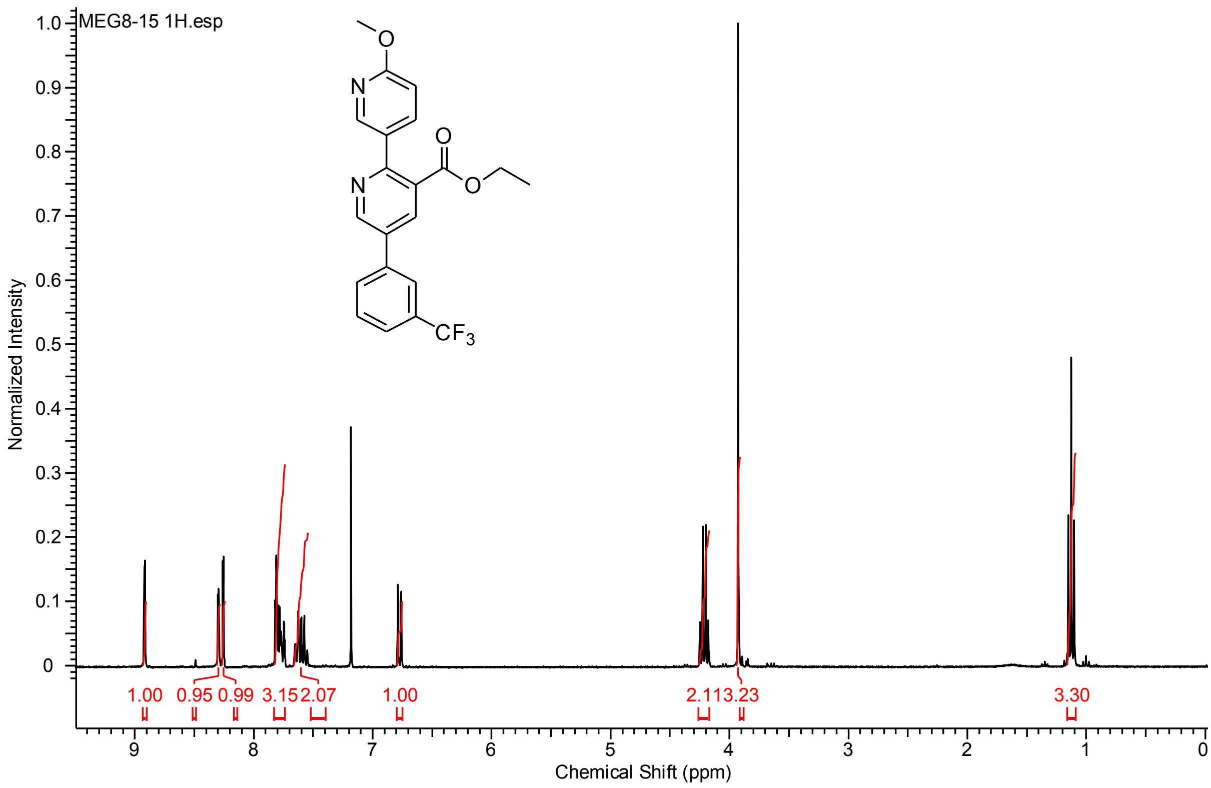

^{13}C NMR of **8t** ($CDCl_3$, 75 MHz)

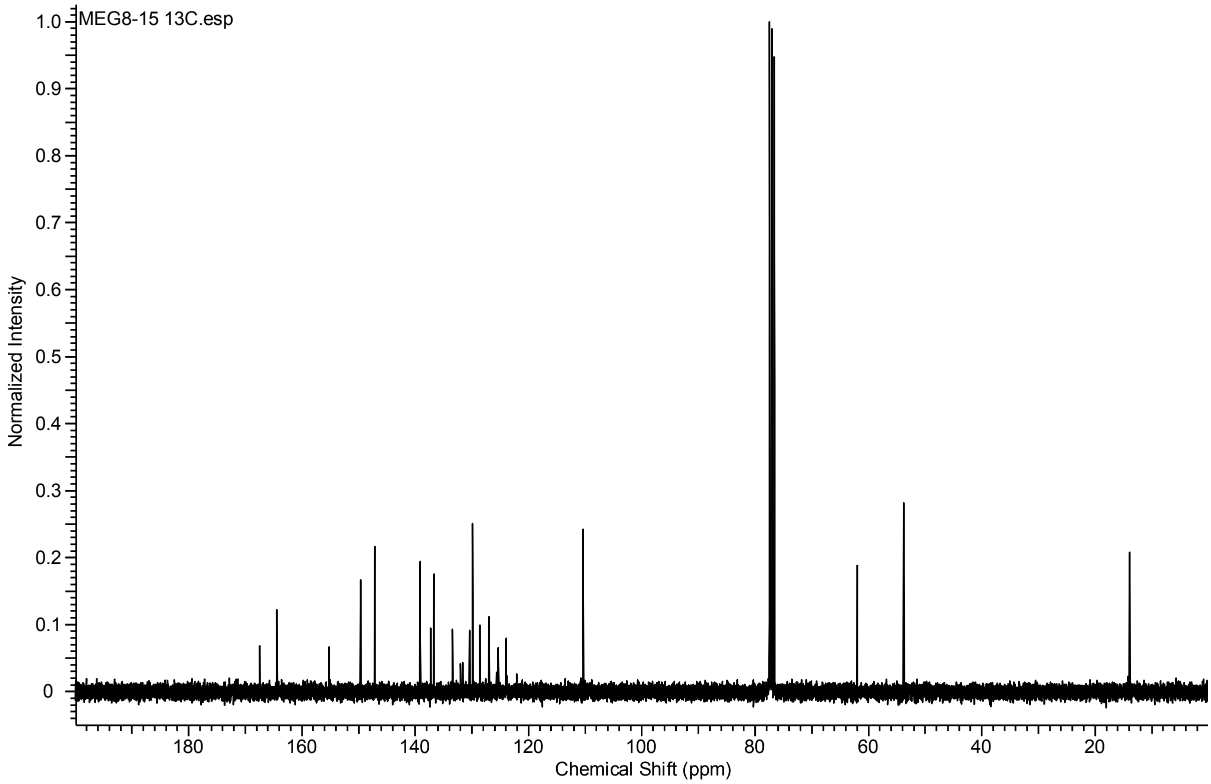

^{1}H NMR of **8u** ($CDCl_3$, 300 MHz)

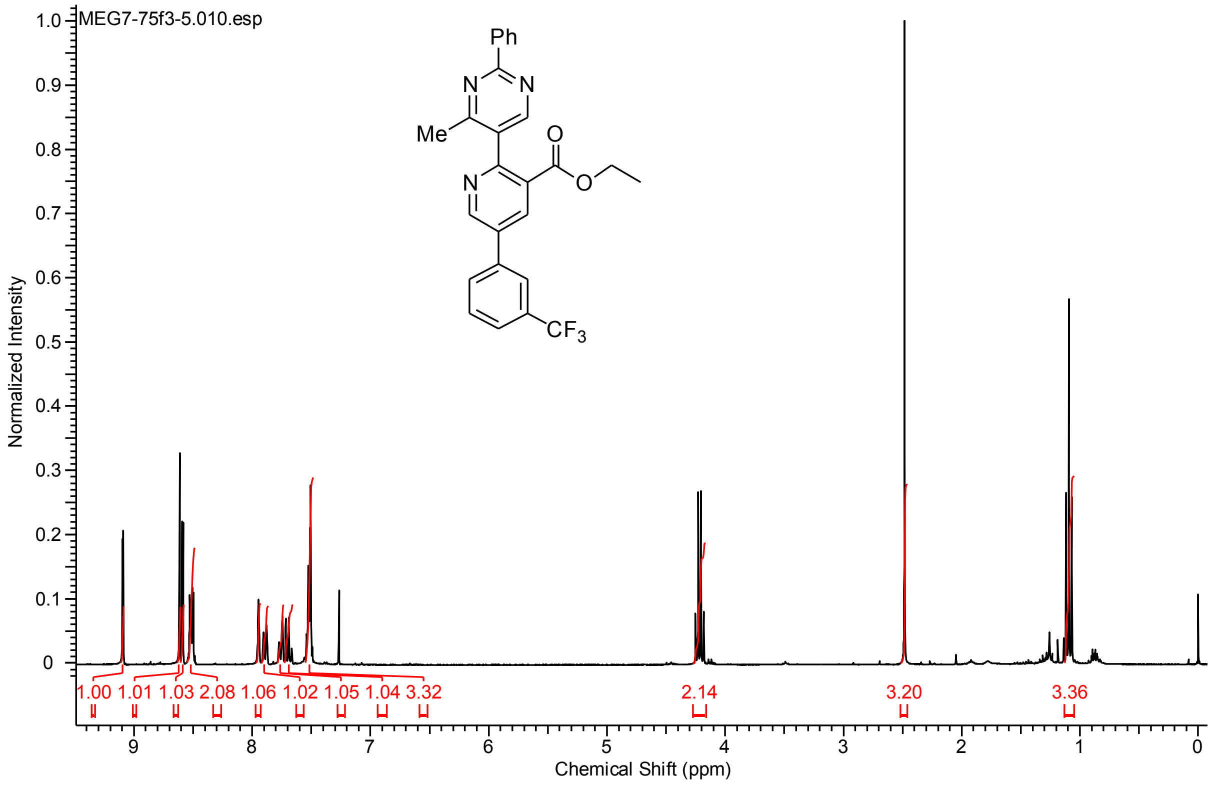

^{13}C NMR of **8u** ($CDCl_3$, 75 MHz)

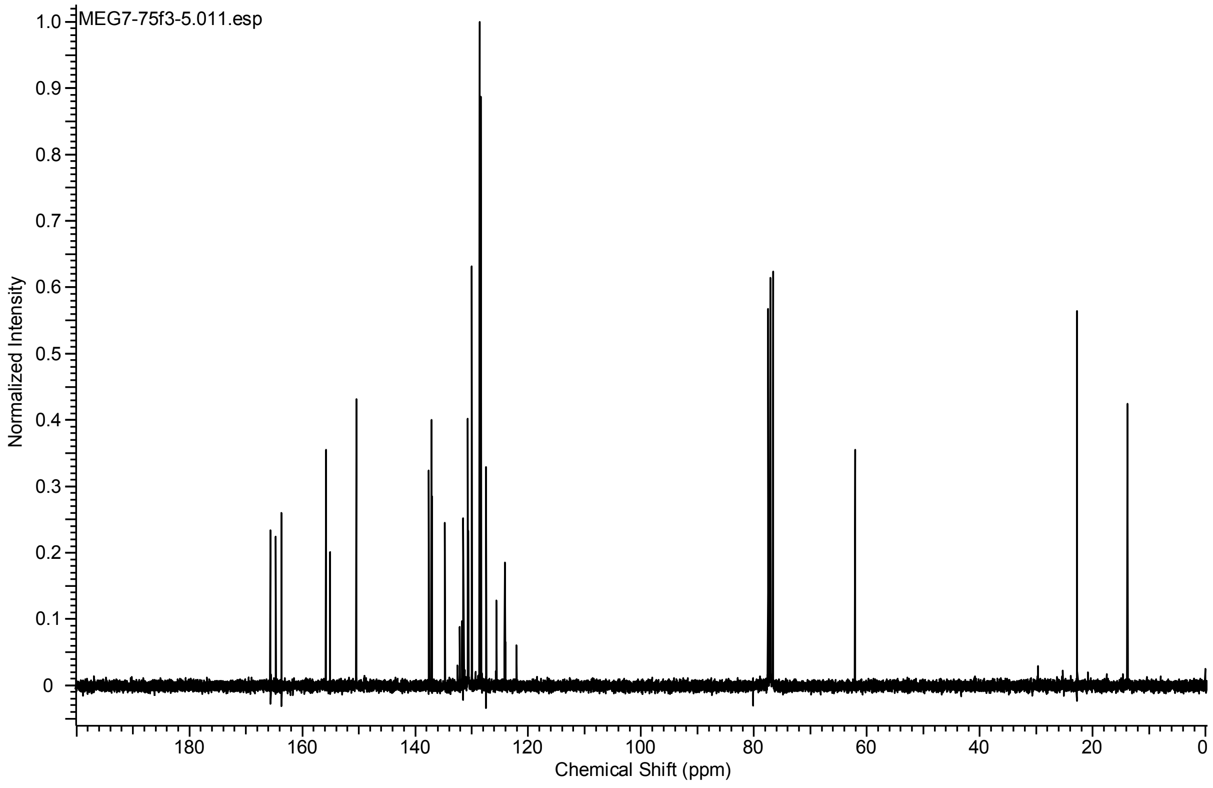

^{1}H NMR of **8v** ($CDCl_3$, 300 MHz)

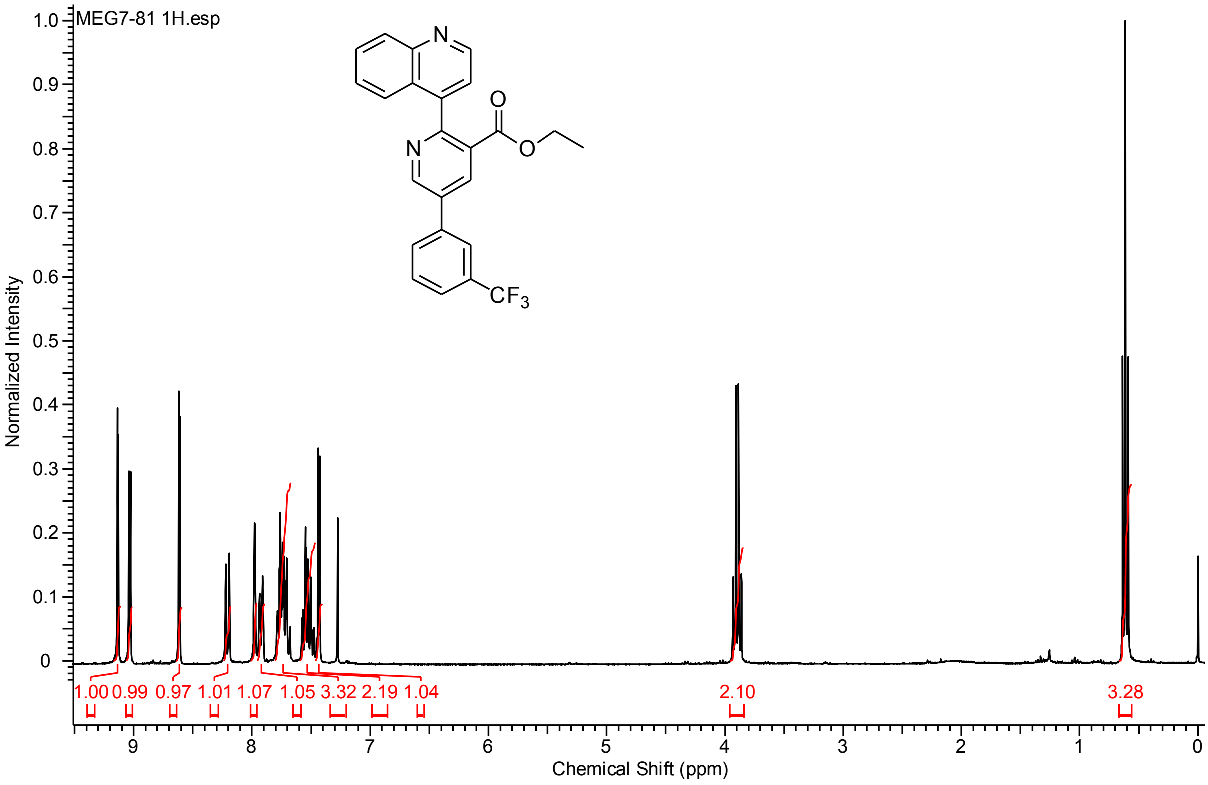

^{1}H NMR of **8v** ($CDCl_3$, 75 MHz)

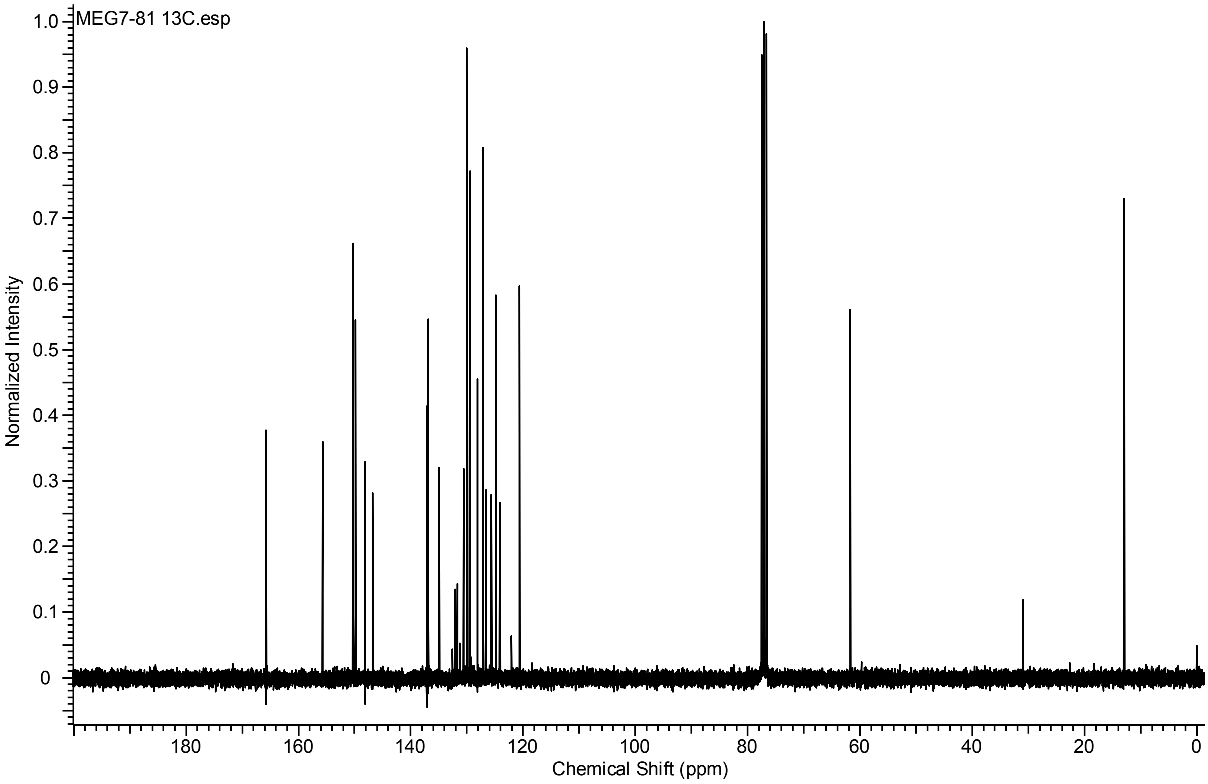

^{1}H NMR of **8w** ($CDCl_3$, 300 MHz)

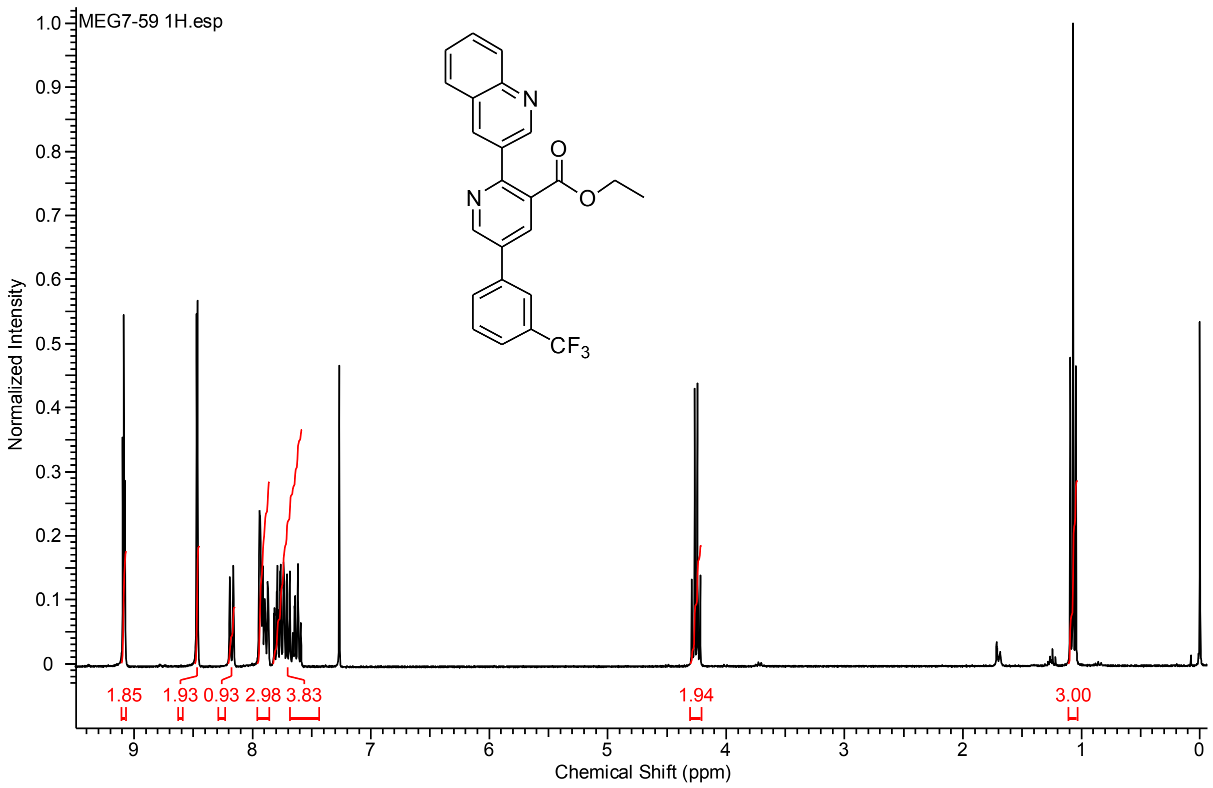

^{13}C NMR of **8w** ($CDCl_3$, 75 MHz)

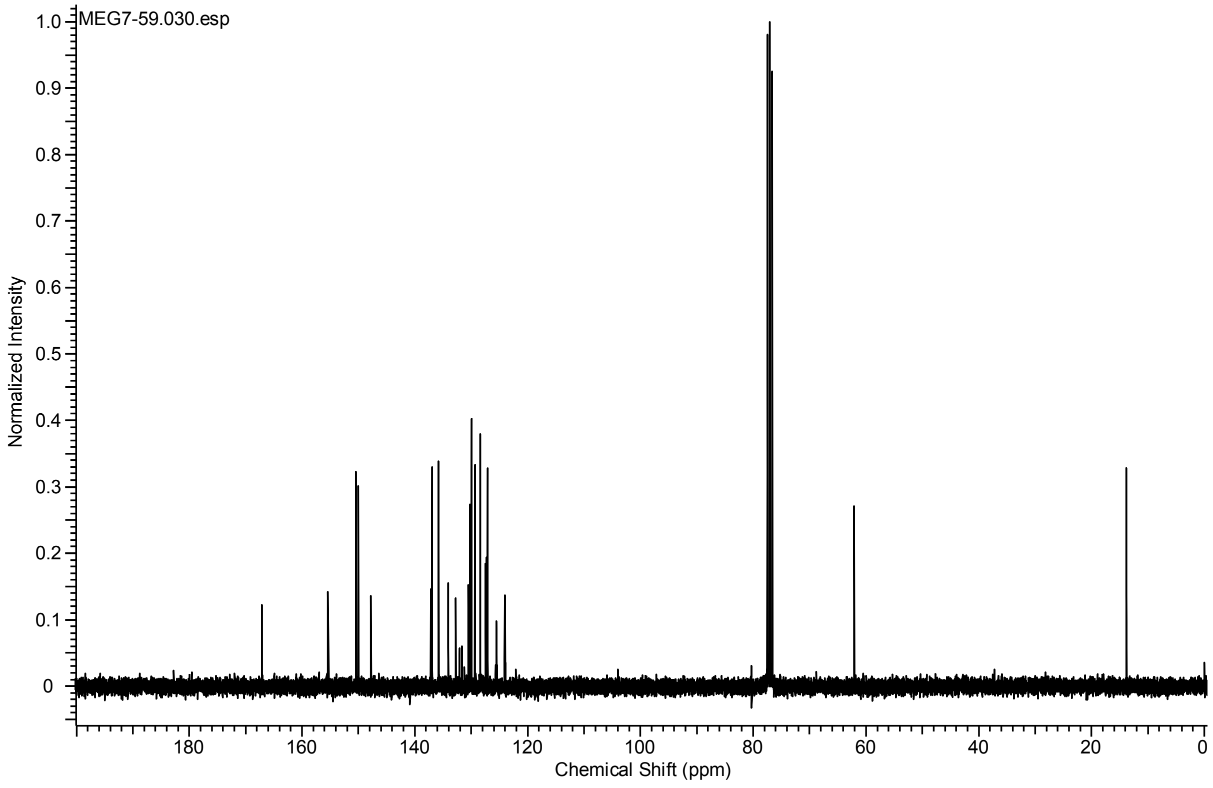

^{1}H NMR of **8x** ($CDCl_3$, 300 MHz)

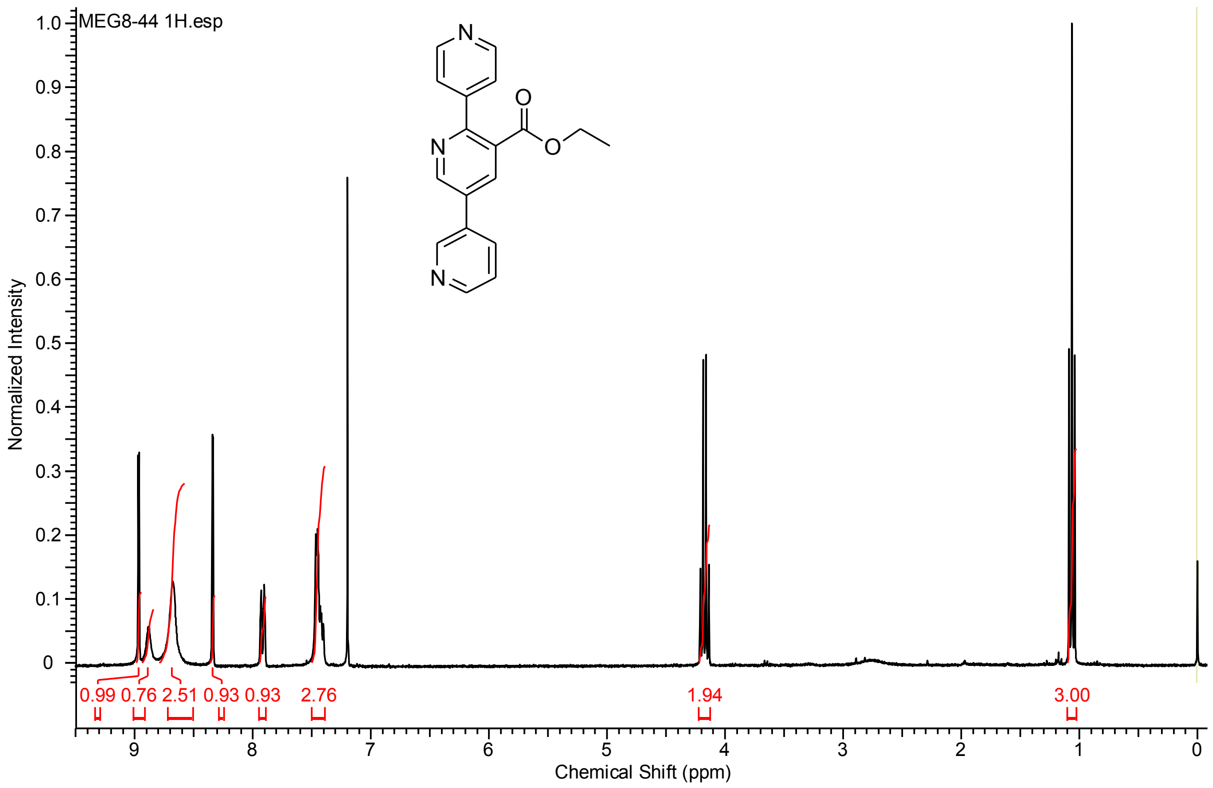

^{13}C NMR of **8x** ($CDCl_3$, 75 MHz)

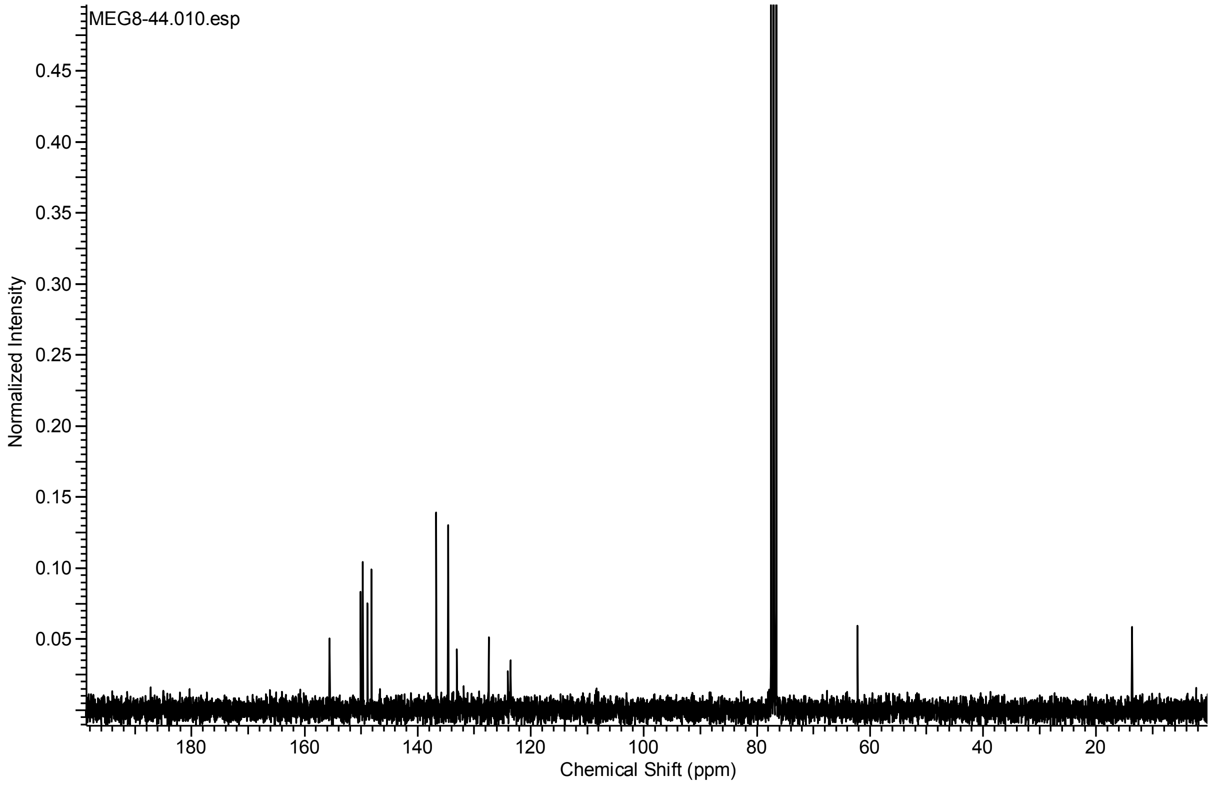

^{1}H NMR of **8y** ($CDCl_3$, 300 MHz)

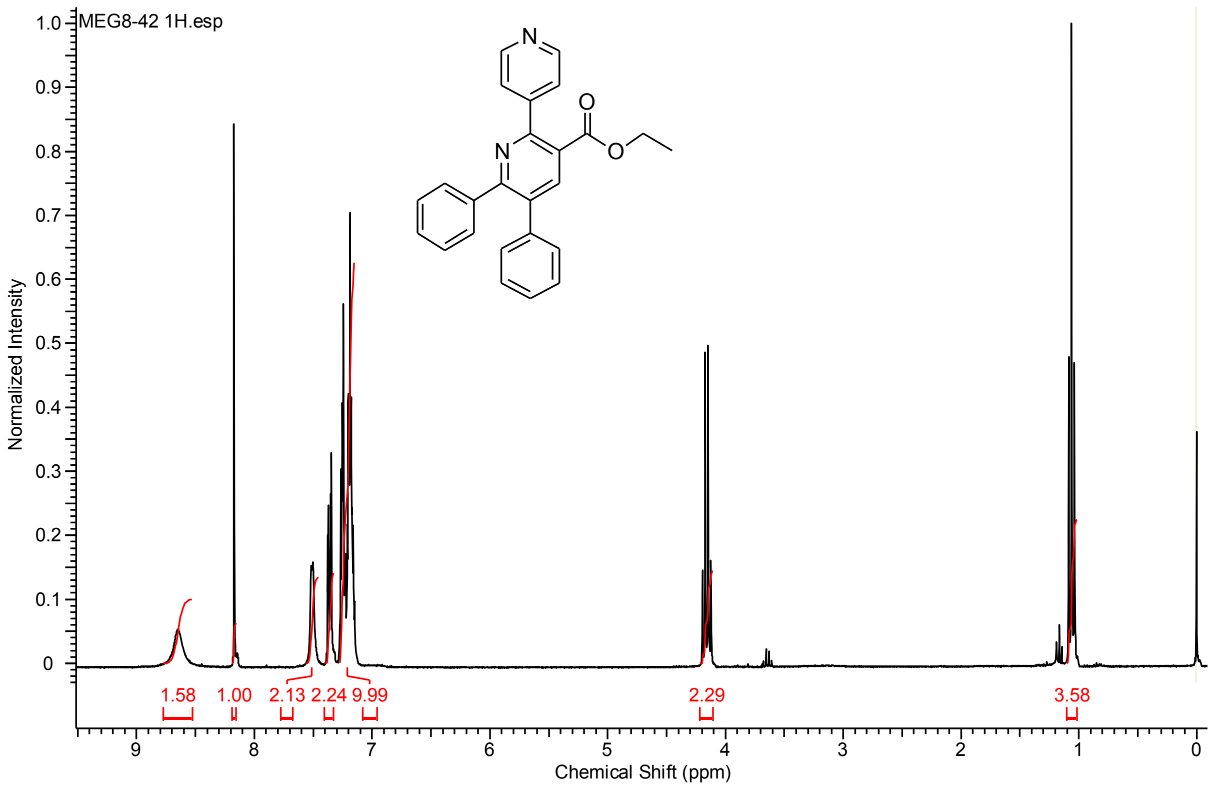

^{13}C NMR of **8y** ($CDCl_3$, 75 MHz)

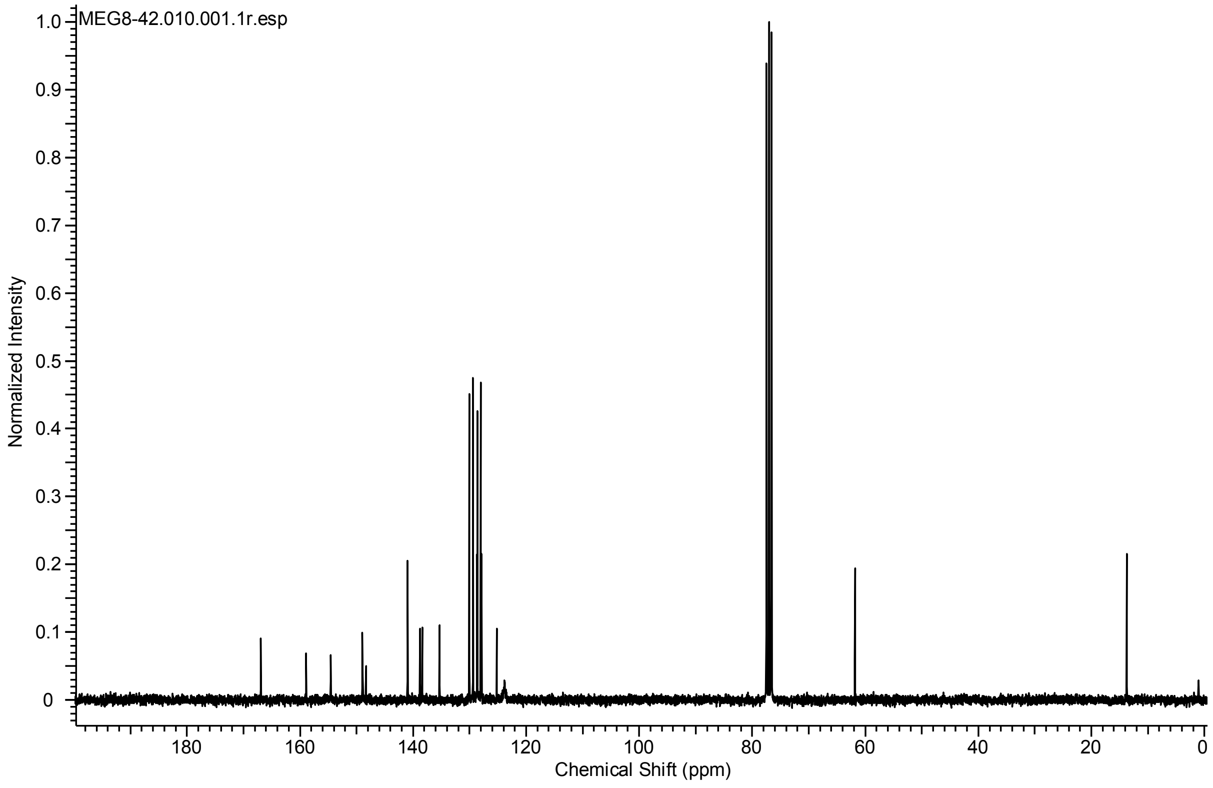

^{1}H NMR of **8z** ($CDCl_3$, 300 MHz)

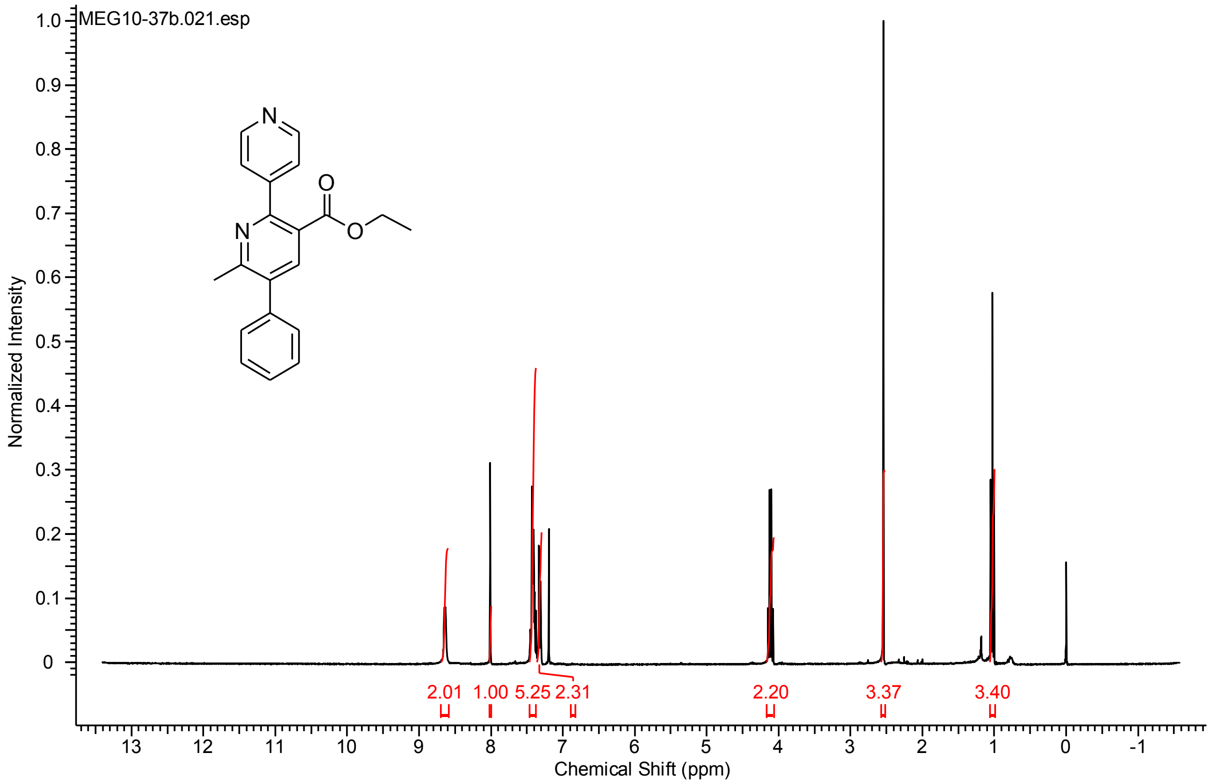

^{13}C NMR of **8z** ($CDCl_3$, 75 MHz)

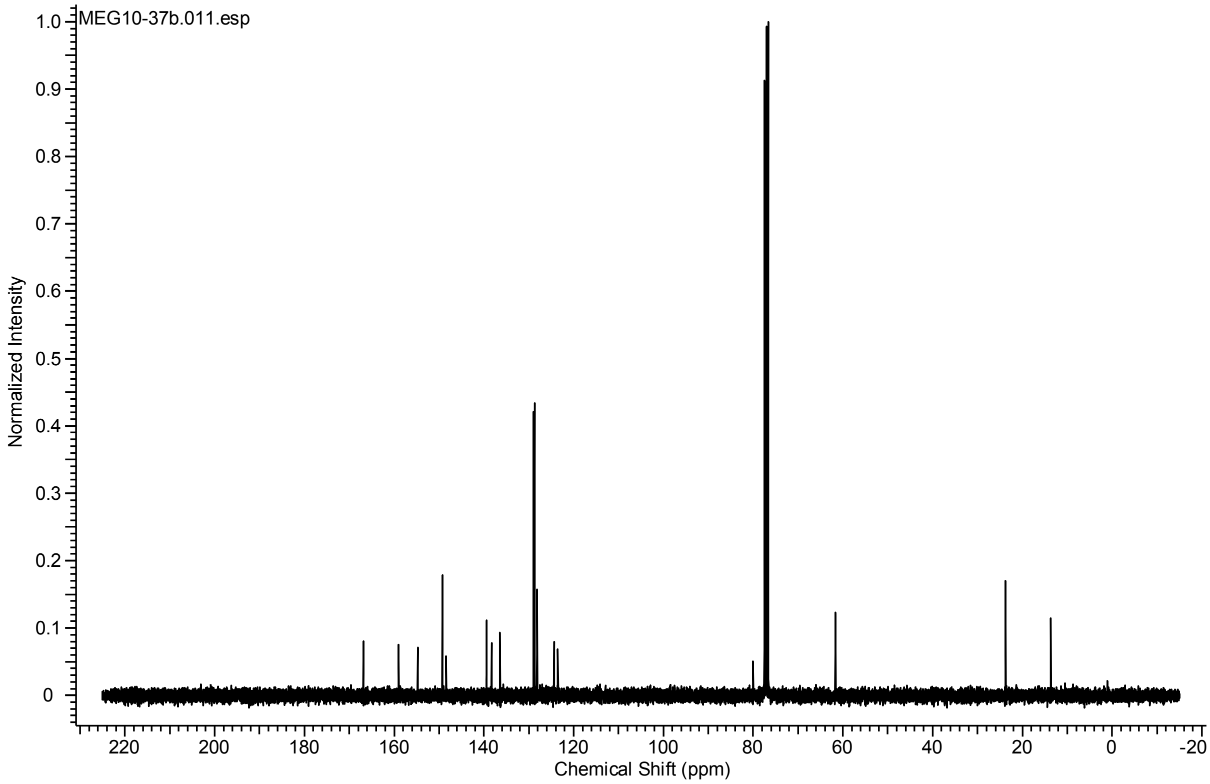

^{1}H NMR of **8aa** ($CDCl_3$, 300 MHz)

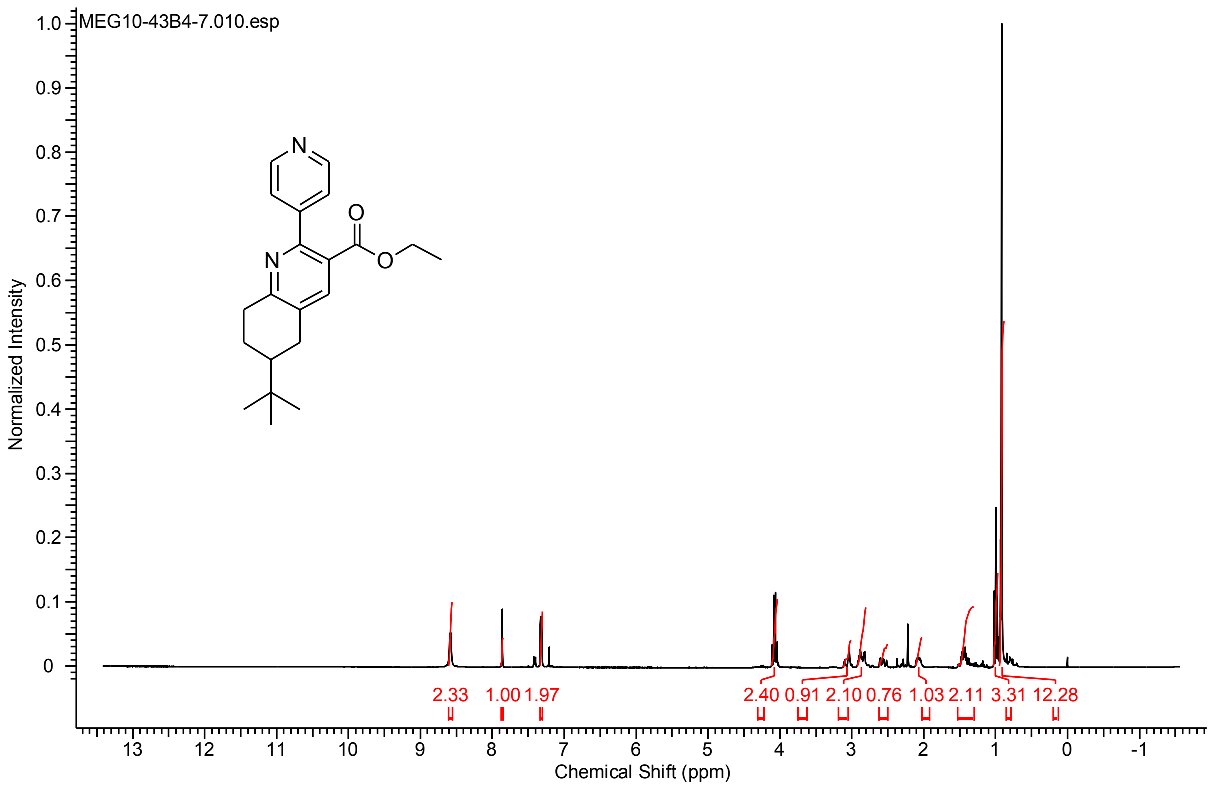

^{13}C NMR of **8aa** ($CDCl_3$, 75 MHz)

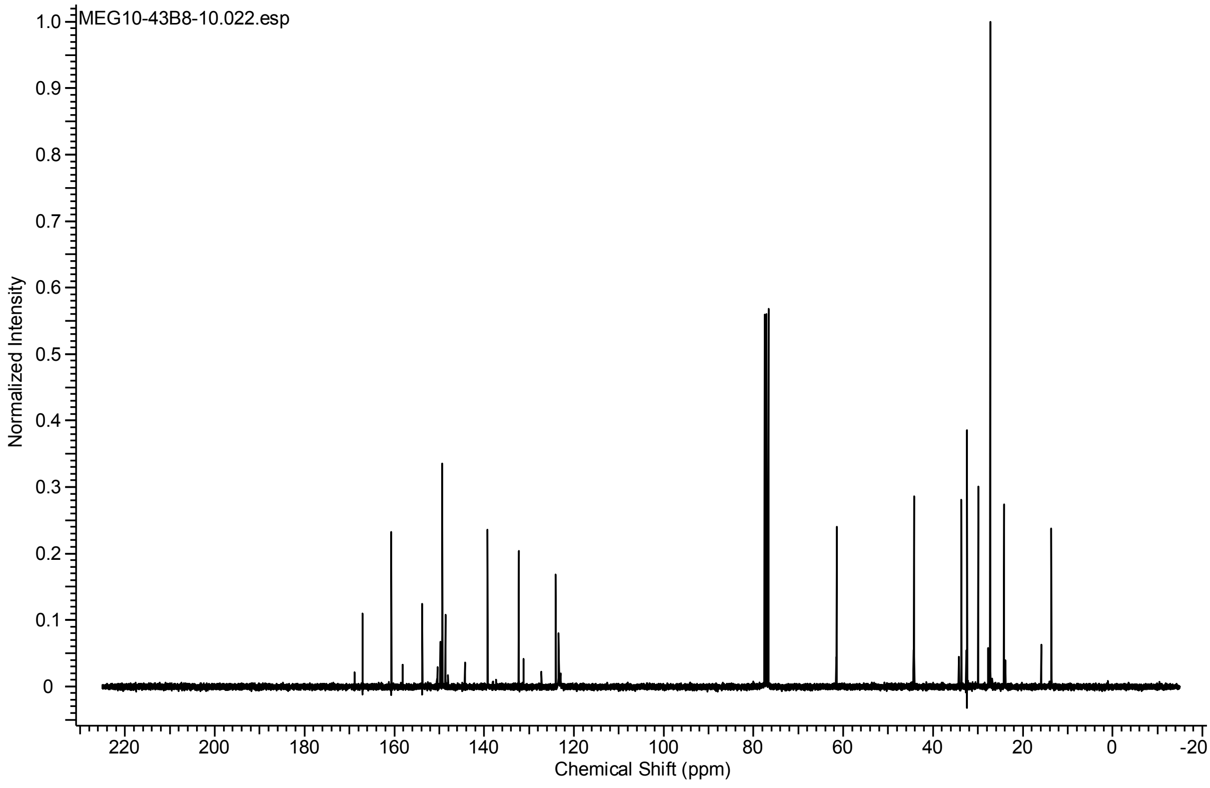

^{1}H NMR of **8ab** ($CDCl_3$, 300 MHz)

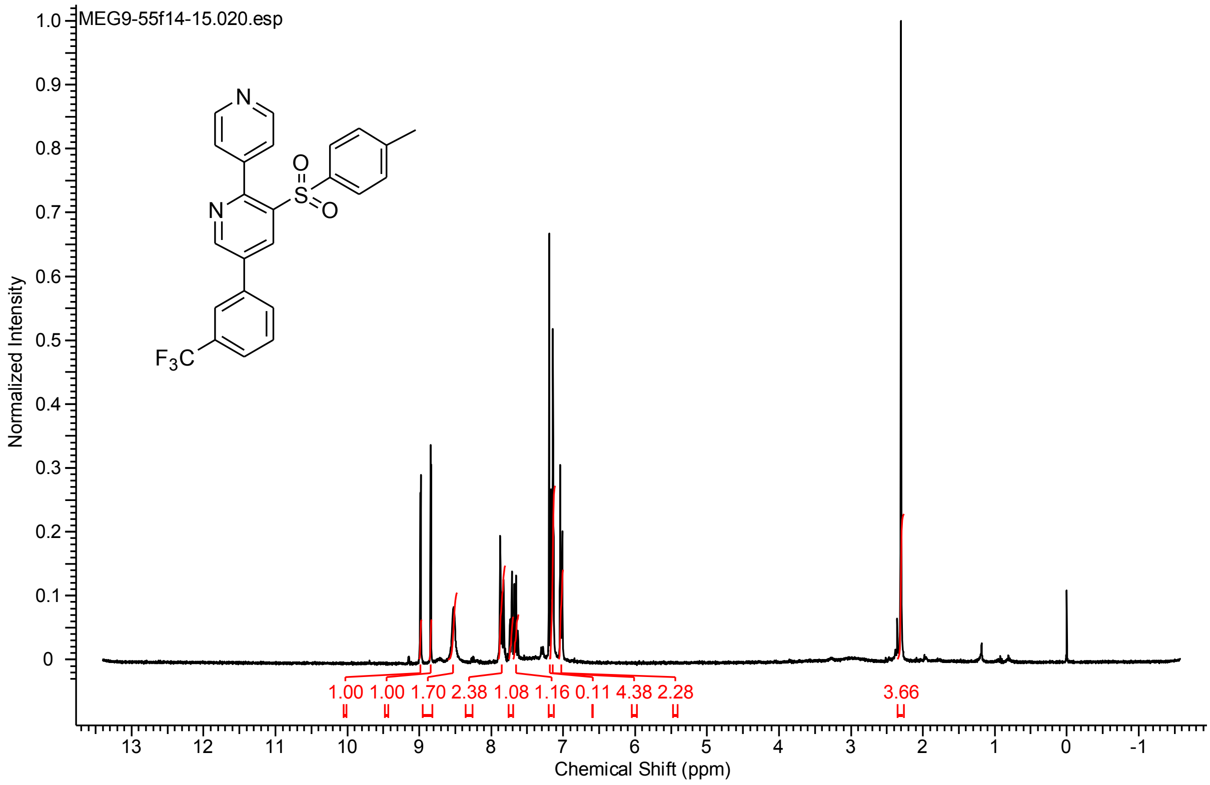

^{13}C NMR of **8ab** ($CDCl_3$, 75 MHz)

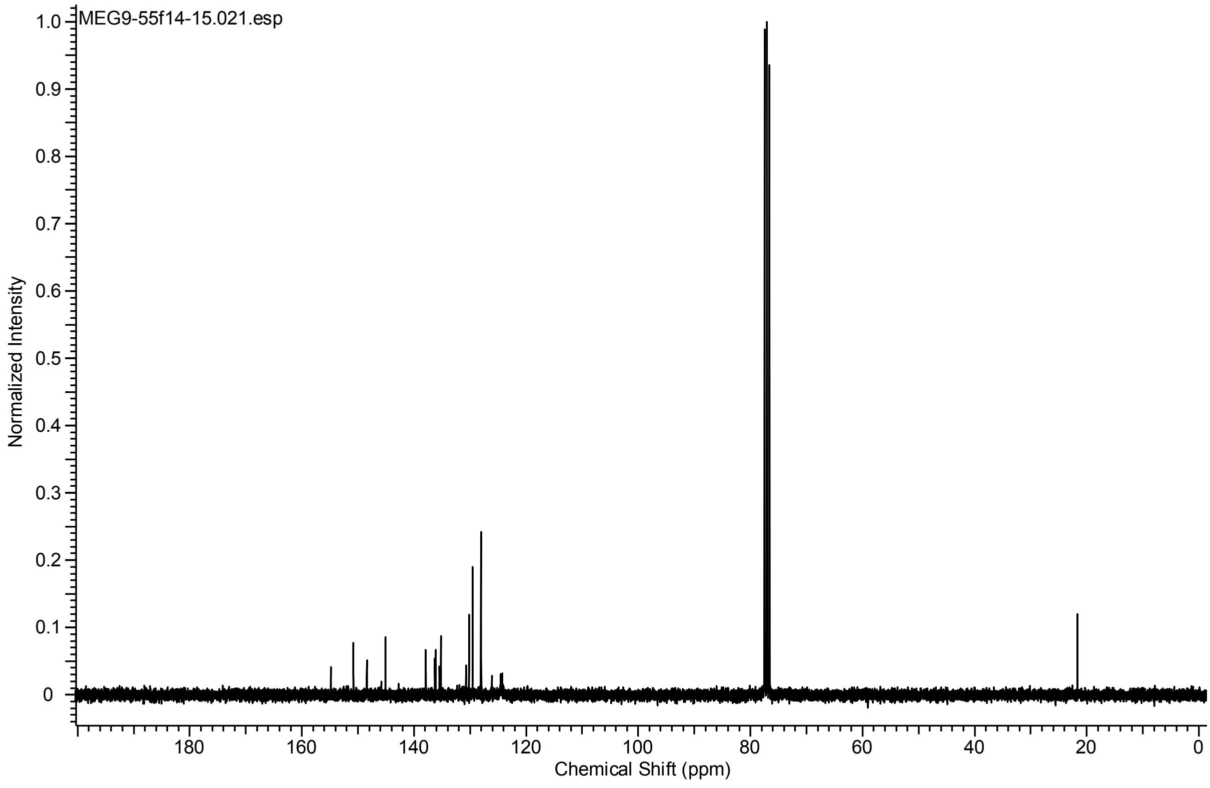

^{1}H NMR of **8ac** ($CDCl_3$, 300 MHz)

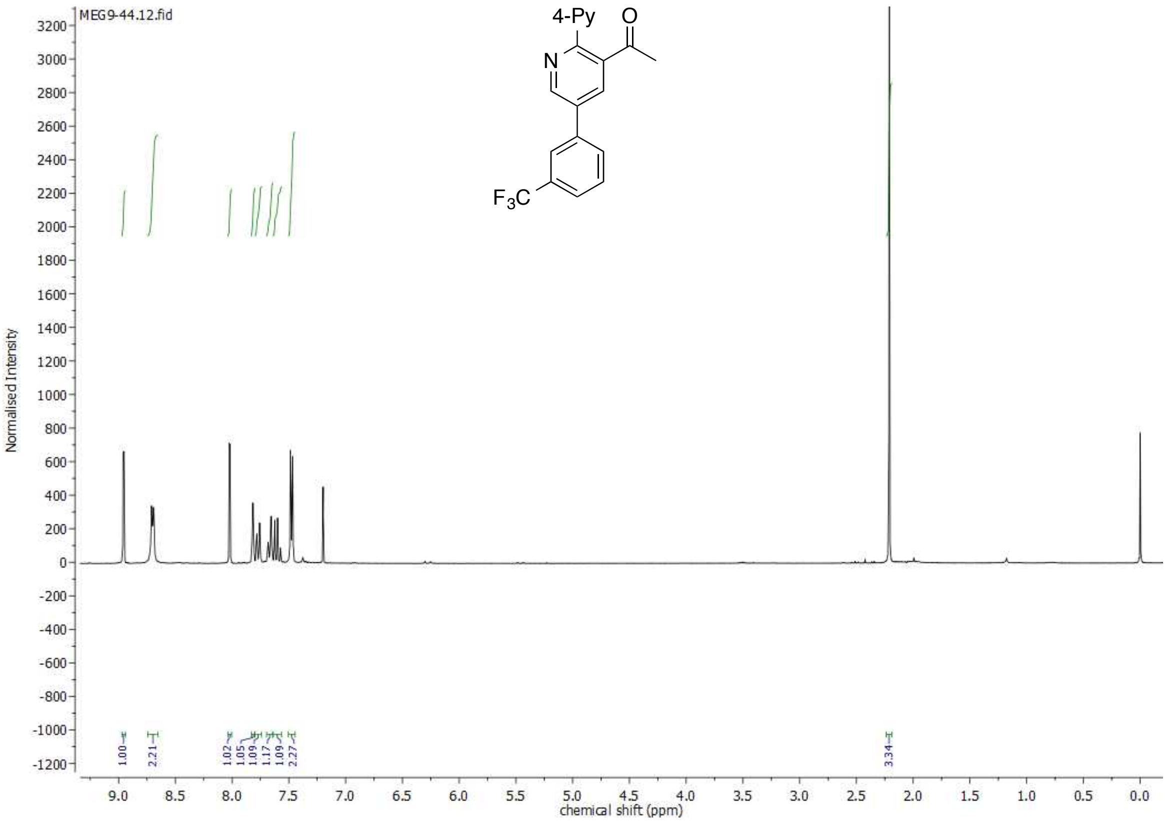

^{13}C NMR of **8ac** ($CDCl_3$, 75 MHz)

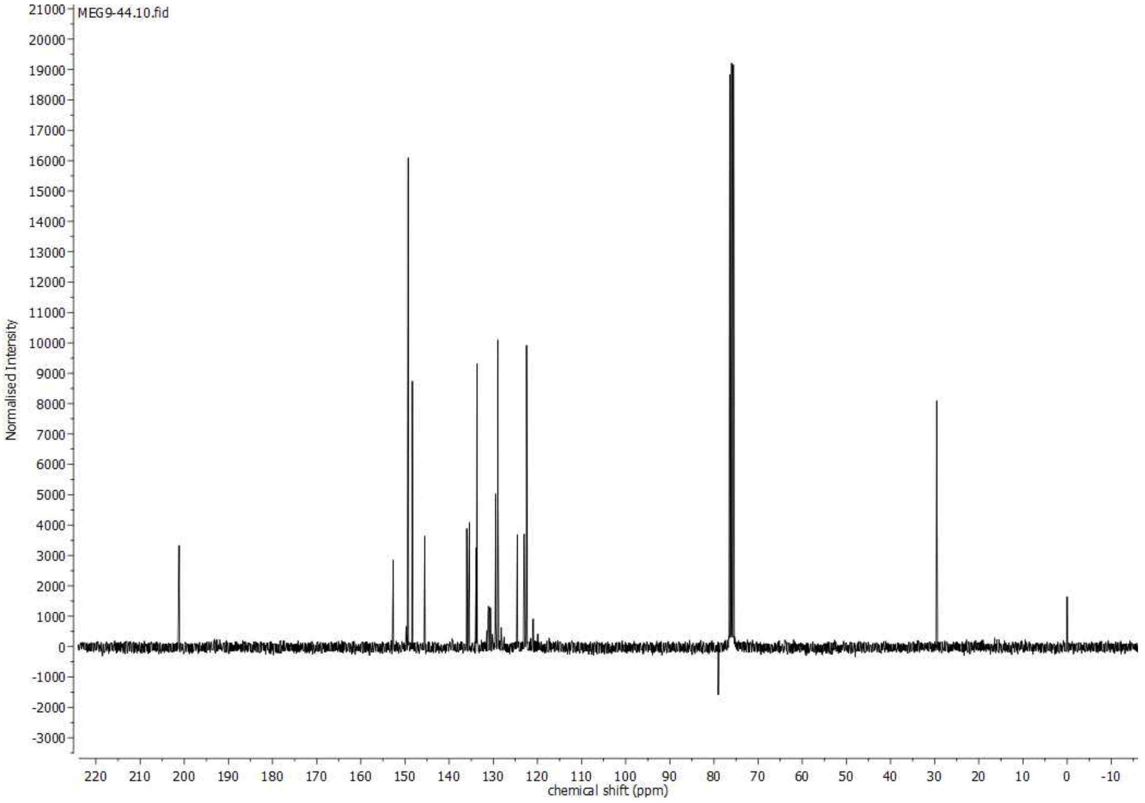

^{1}H NMR of **8ad** and **9b** ($CDCl_3$, 300 MHz)

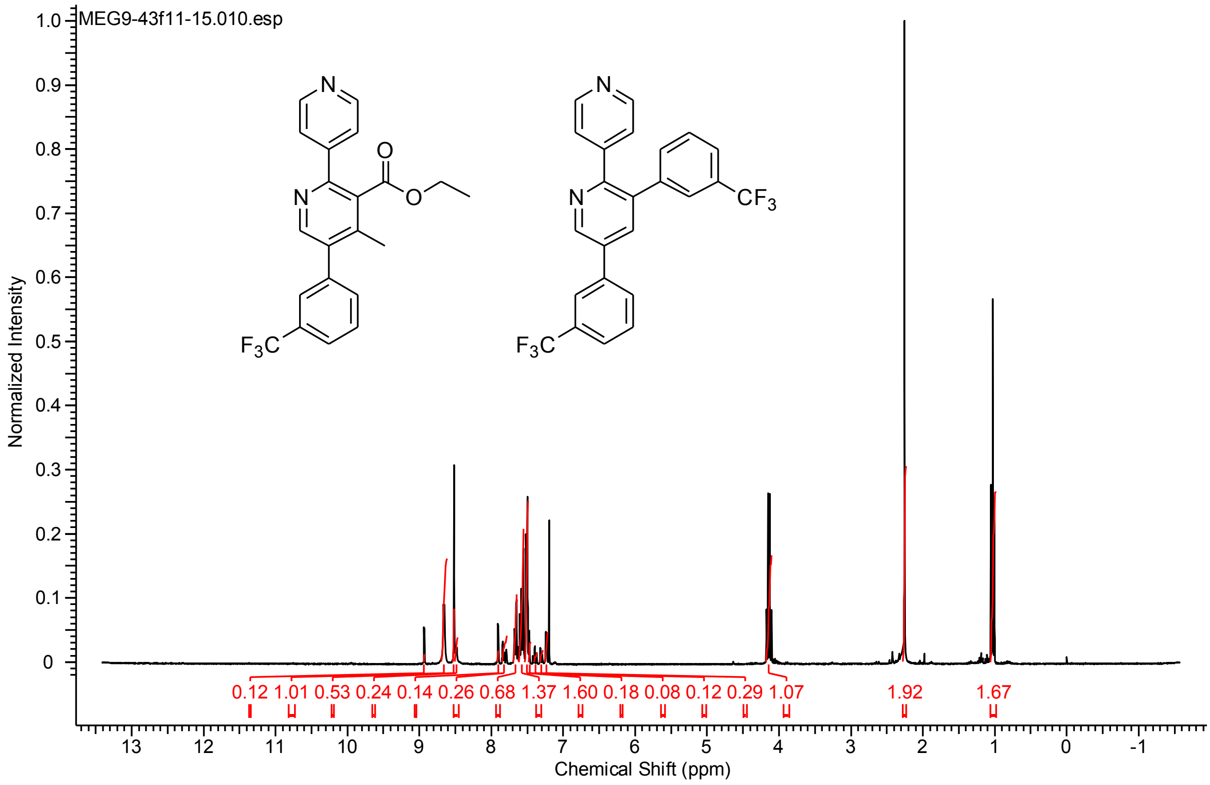

^{13}C NMR of **8ad and 9b** ($CDCl_3$, 75 MHz)

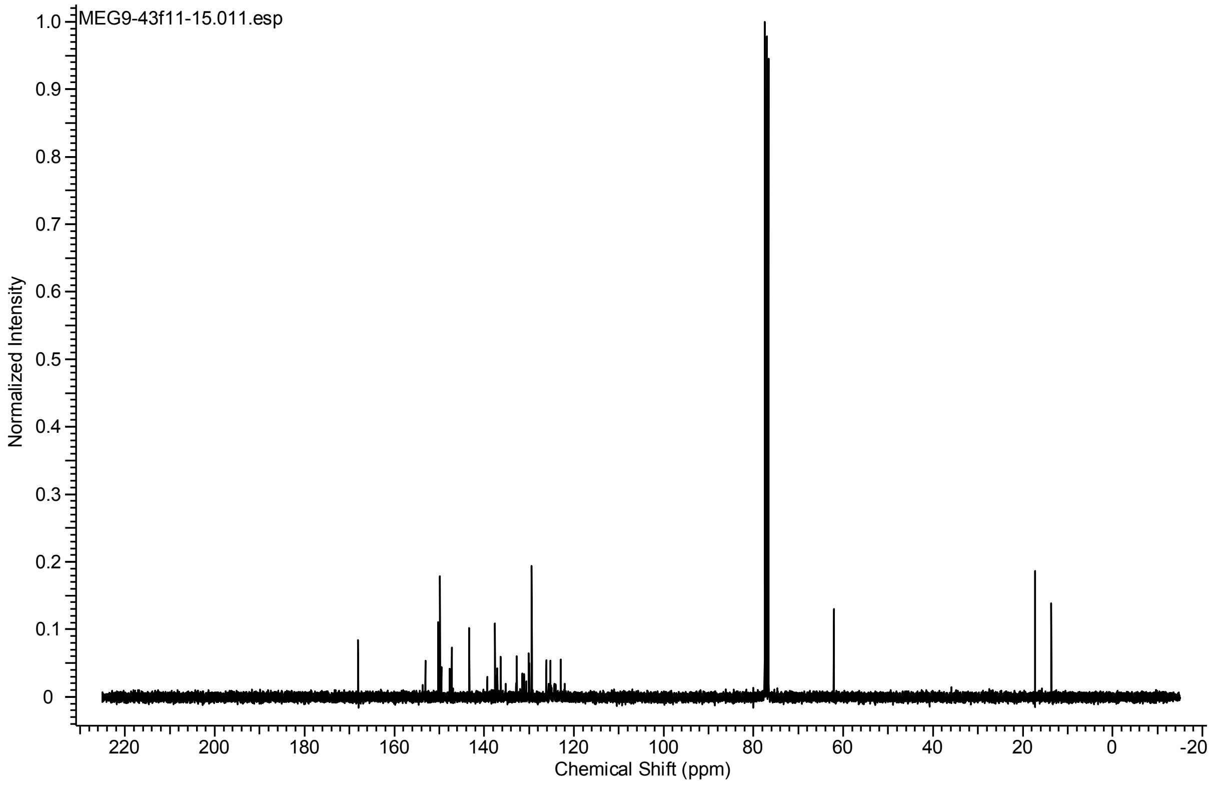

^{1}H NMR of **8ae** ($CDCl_3$, 300 MHz)

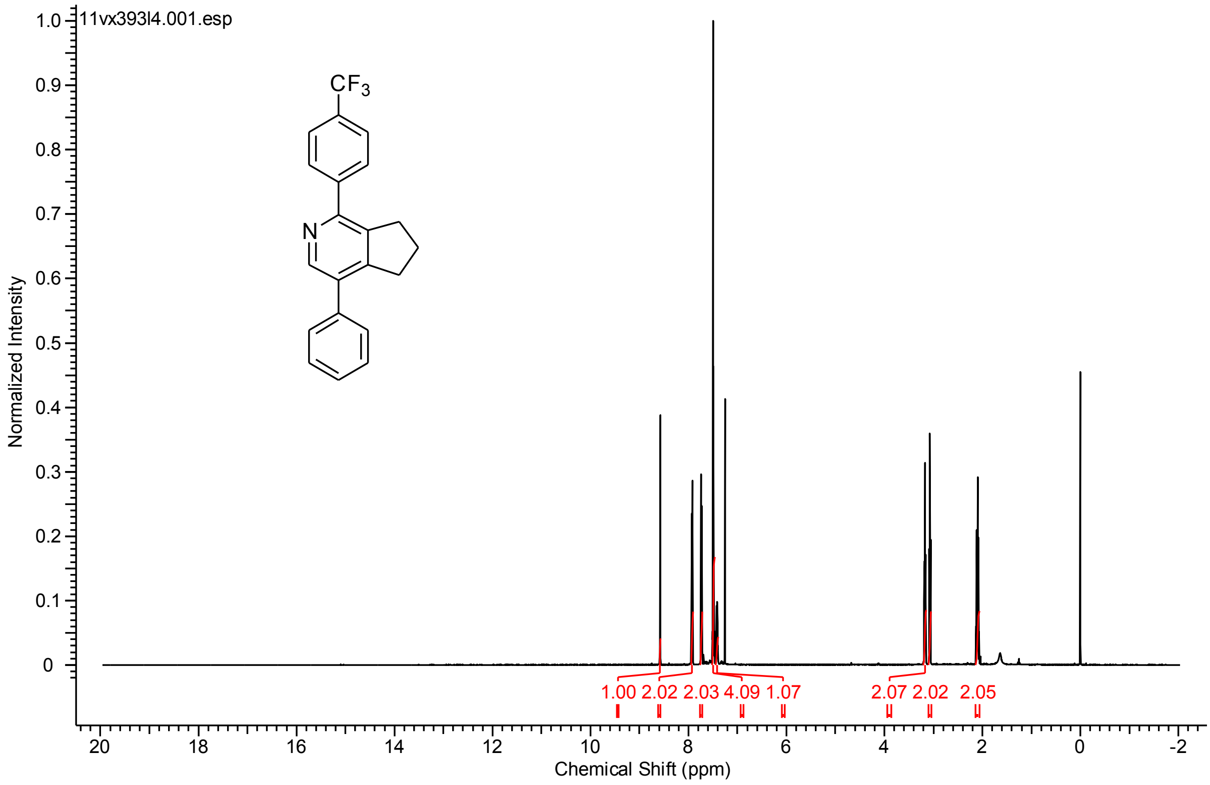

^{13}C NMR of **8ae** ($CDCl_3$, 75 MHz)

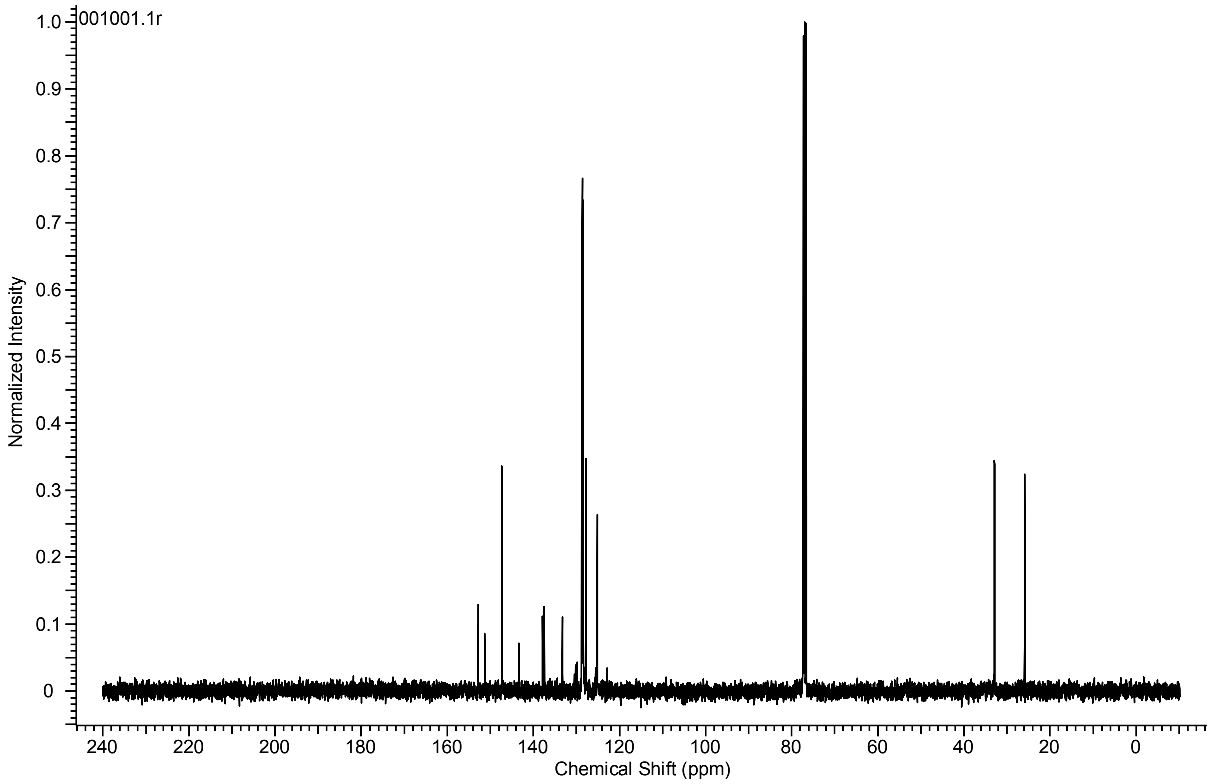

Supplement: RA-012-D2RA05018H-s001 [file RA-012-D2RA05018H-s001.pdf]
